# Quality of Pregnancy Dating and Obstetric Interventions During Labor: Retrospective Database Analysis

**DOI:** 10.2196/14109

**Published:** 2020-04-15

**Authors:** Zilma Silveira Nogueira Reis, Juliano De Souza Gaspar, Gabriela Luiza Nogueira Vitral, Vitor Barbosa Abrantes, Ingrid Michelle Fonseca de-Souza, Maria Tereza Silveira Moreira, Regina Amélia Lopes Pessoa Aguiar

**Affiliations:** 1 Center of Health Informatics Universidade Federal de Minas Gerais Belo Horizonte Brazil; 2 Faculty of Medicine Universidade Federal de Minas Gerais Belo Horizonte Brazil

**Keywords:** gestational age, parturition, ultrasound, pregnancy dating, information systems

## Abstract

**Background:**

The correct dating of pregnancy is critical to support timely decisions and provide obstetric care during birth. The early obstetric ultrasound assessment before 14 weeks is considered the best reference to assist in determining gestational age (GA), with an accuracy of ±5 to 7 days. However, this information is limited in many settings worldwide.

**Objective:**

The aim of this study is to analyze the association between the obstetric interventions during childbirth and the quality of GA determination, according to the first antenatal ultrasound assessment, which assisted the calculation.

**Methods:**

This is a hospital-based cohort study using medical record data of 2113 births at a perinatal referral center. The database was separated into groups and subgroups of analyses based on the reference used by obstetricians to obtain GA at birth. Maternal and neonatal characteristics, mode of delivery, oxytocin augmentation, and forceps delivery were compared between groups of pregnancies with GA determination at different reference points: obstetric ultrasound assessment 14 weeks, 20 weeks, and ≥20 weeks or without antenatal ultrasound (suboptimal dating). Ultrasound-based GA information was associated with outcomes between the interest groups using chi-square tests, odds ratios (OR) with 95% CI, or the Mann-Whitney statistical analysis.

**Results:**

The chance of nonspontaneous delivery was higher in pregnancies with 14 weeks ultrasound-based GA (OR 1.64, 95% CI 1.35-1.98) and 20 weeks ultrasound-based GA (OR 1.58, 95% CI 1.31-1.90) when compared to the pregnancies with ≥20 weeks ultrasound-based GA or without any antenatal ultrasound. The use of oxytocin for labor augmentation was higher for 14 weeks and 20 weeks ultrasound-based GA, OR 1.41 (95% CI 1.09-1.82) and OR 1.34 (95% CI 1.04-1.72), respectively, when compared to those suboptimally dated. Moreover, maternal blood transfusion after birth was more frequent in births with suboptimal ultrasound-based GA determination (20/657, 3.04%) than in the other groups (14 weeks ultrasound-based GA: 17/1163, 1.46%, *P*=.02; 20 weeks ultrasound-based GA: 25/1456, 1.71%, *P*=.048). Cesarean section rates between the suboptimal dating group (244/657, 37.13%) and the other groups (14 weeks: 475/1163, 40.84%, *P*=.12; 20 weeks: 584/1456, 40.10%, *P*=.20) were similar. In addition, forceps delivery rates between the suboptimal dating group (17/657, 2.6%) and the other groups (14 weeks: 42/1163, 3.61%, *P*=.24; 20 weeks: 46/1456, 3.16%, *P*=.47) were similar. Neonatal intensive care unit admission was more frequent in newborns with suboptimal dating (103/570, 18.07%) when compared with the other groups (14 weeks: 133/1004, 13.25%, *P*=.01; 20 weeks: 168/1263, 13.30%, *P*=.01), excluding stillbirths and major fetal malformations.

**Conclusions:**

The present analysis highlighted relevant points of health care to improve obstetric assistance, confirming the importance of early access to technologies for pregnancy dating as an essential component of quality antenatal care.

## Introduction

The correct dating of gestation is trigger information for health professionals to make timely decisions for care. Caregivers should be vigilant with the recording and retrieving of gestational age (GA) during antenatal care and birth [1,2], as well as the availability and quality of clinical data impacting caring [3]. Nonetheless, there are different references to assist in determining the GA, which all have varying accuracy [4]. Current methods to calculate GA have disadvantages due to the high costs of ultrasound assessment, inaccurate dates of the last menstrual period, and the lack of precision in neonatal maturity scores [2,4,5]. GA is oftentimes calculated using the difference between the date of birth and the referential daters from the beginning of gestation, such as the last menstrual period, ultrasound assessment, or markers of pregnancy evolution like fundal height. After birth, neonatal maturity scores are used to assist professionals to face unreliable or unknown dating of pregnancy [4]. Ultrasound for fetal assessment in early pregnancy (>7 weeks but <14 weeks) is considered the best dating method for gestational chronology, with a given error of ±5 to 7 days [1]. Despite a ±10-day margin of error, GA determined by ultrasound assessments ≥14 weeks but <20 weeks is still a reasonable antenatal record to estimate the GA when an early fetal ultrasound is missing [1,6,7]. A pregnancy dating based on ultrasonography performed after 20 weeks is considered suboptimally dated [1].

Accurate GA calculation remains a priority in public health [6]. According to the World Health Organization (WHO), several countries are unable to adequately collect minimum data for each birth [4,8]. The quality of data has an impact on prematurity rates (ranging from 6.2%-17.5%) and small-for-gestational-age rates, and varies according to the methodology of GA estimation [9], country, and the quality of the report [10].

At the end of pregnancy, labor induction, oxytocin augmentation, instrumental vaginal delivery, and cesarean sections are clearly necessary for some high-risk situations. Some preterm births are medically induced, and approximately half of preterm births are idiopathic [11]. Nevertheless, unnecessary obstetric interventions are continually increasing around the world; a situation that is made worse when GA is unreliable or unknown [5,11]. The impact that the quality of antenatal references used to support GA determination has on obstetric decisions and perinatal outcomes has not sufficiently been elucidated. We tested the hypothesis that the credibility of GA information retrieved at birth might be associated with the medical choices during obstetric interventions. This study aims to analyze the association between the obstetric interventions during childbirth and the quality of GA determination according to the first antenatal ultrasound assessment used for the calculation.

## Methods

### Study Design

This hospital-based cohort study retrospectively evaluated the quality of pregnancy dating and obstetric information in a computerized medical database, SISMater, dedicated to registering inpatient birth records [12]. All 2113 medical records from the dataset of live or stillborn infants delivered between October 2016, and September 2017, at the Hospital das Clínicas of the Universidade Federal de Minas Gerais in Brazil were included. The institutional review boards approved the research protocol (number: CAAE 10286913.3.0000.5149), dismissing individual written informed consent.

### Dataset and Data Collection

**Figure 1:**
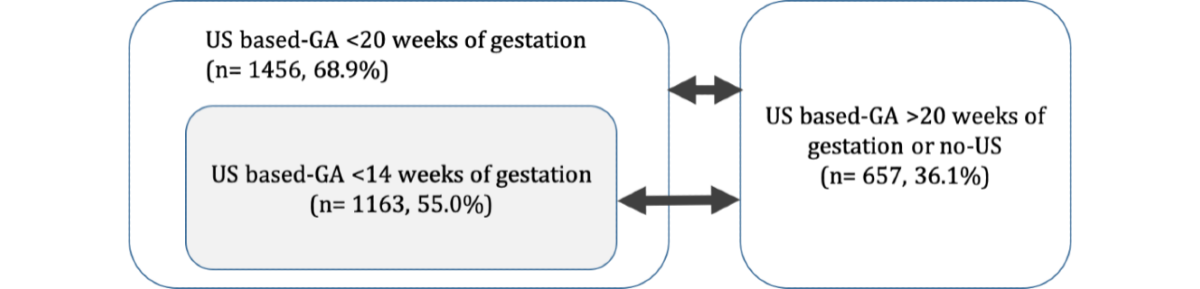
Comparison groups for ultrasound-based gestation age determination (N=2113). GA: gestational age; US: ultrasound.

For the analysis, the database was organized into groups based on ultrasonography reference used by obstetricians to assist in determining GA. The group of childbirths with the first obstetric ultrasound assessment <20 weeks of gestation and their subgroup of the first obstetric ultrasound assessment <14 weeks, were compared to the group of pregnancies suboptimally dated with ultrasound assessment ≥20 weeks or without any ultrasound recorded (Figure 1).

The medical staff collected data on childbirth scenarios and all maternal and neonatal hospital stays using a system with a structured interface format [12]. The authors intend to share the minimum anonymized dataset necessary to replicate study findings (Multimedia Appendix 1). The data can be used under reasonable request to the corresponding author, as the citation of the original study is required.

The electronic medical record consists of the obstetric care and neonatal care reports. In the obstetric care section, the GA was automatically calculated by the system after input of the first trimester ultrasound findings when available. Cases in which ultrasound information that was <14 weeks of gestation was lacking or when the ultrasound was performed at 14 weeks or later, the GA was primarily calculated by the physician who assisted the birth, and the result was recorded in the electronic medical record system. For this, obstetricians used their best judgment to estimate the GA, either based on the last menstrual period or the ultrasound results. The information was considered missing if GA was reported in the electronic medical record as unknown. We had no access to the date of the last menstrual period in this database.

### Perinatal Characteristics

Records on perinatal characteristics were compared between groups of interest to enhance the external validity of primary outcomes. Neonatal resuscitation referred to any of the steps of the recommended actions at birth [13]. The fetal or newborn mortality variable included stillbirths and newborn deaths during the hospital stay. Maternal transfusion after birth considered all derivatives of blood. Fetal and neonatal mortality, neonatal resuscitation, and neonatal intensive care unit (NICU) admission were presented, considering the presence of major malformations. Even in the presence of severe birth defects, elective abortion is not permitted by law in Brazil, except in the case of anencephaly [14].

### Primary Outcomes

The obstetric interventions chosen as primary outcomes included maternal admission for nonspontaneous vaginal delivery, oxytocin augmentation during labor, cesarean section, and forceps delivery. Maternal admissions for the interruption of pregnancy without natural labor contractions and induced labors were considered nonspontaneous vaginal deliveries. University hospital protocols to manage labor rely on the best clinical and obstetric practices geared toward maternal-fetal diagnosis, labor management, neonatal care, and hospital or intensive care admissions.

### Statistics

Descriptive statistics assessed the variables, depending on data distribution. Quantitative variables were presented as means, SDs, medians, and IQRs. Qualitative variables were presented as absolute values and percentages. The first obstetric ultrasound available was shown using the Pareto chart to overview the moment of GA assessment by ultrasonography in this cohort of childbirths. The quality of GA determination, according to the obstetric ultrasound assessment used to assist the calculation, was associated with the records of labor interventions using the chi-square test, odds ratio (OR) with 95% CI, or the Mann-Whitney statistical analysis. The significance level, adjusted for the hypothesis test, was set at 5%. The statistical program SPSS version 22.0 (IBM Corp, Armonk, NY) was used for analysis.

## Results

**Figure 2:**
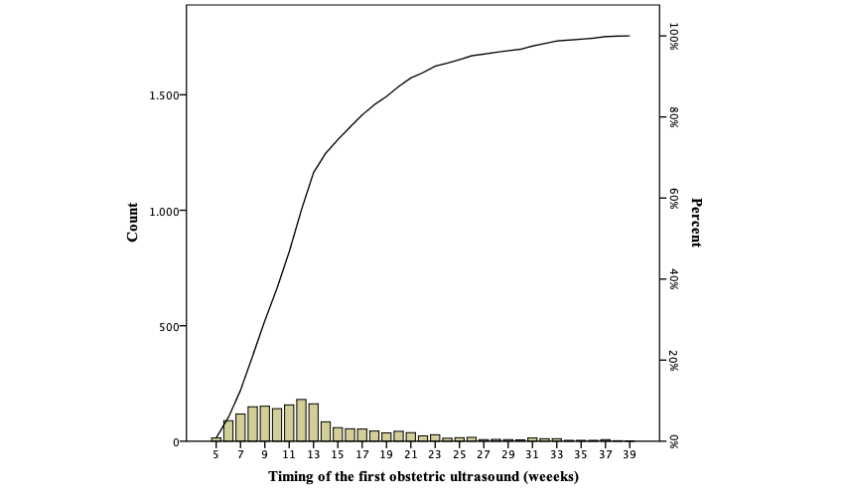
Pareto chart with the distribution of births according to the first obstetric ultrasound retrieved by clinical histories (n=1695).

A substantial proportion of the obstetric histories recorded in the dataset had at least one antenatal ultrasound assessment prior to the hospital admission. The distribution of the first obstetric ultrasound assessment for pregnancy dating is presented in Figure 2.

### Perinatal Characteristics

Table 1 presents the clinical and obstetric characteristics of the cohort, considering valid records. One-third of these newborns (615/2113, 29.11%) were delivered by mothers who received prenatal care at the local unit. The remaining mothers came from city hospitals, referenced to the perinatal center as high-risk pregnancies. Most childbirths were born from a gestation complicated by diseases or by nonspontaneous vaginal delivery. Of the 2113 total samples, there were 2030 (96.07%) live births. In addition, 76 (3.60%) newborns were siblings.

**Table 1:** Perinatal characteristics of the cohort.

Clinical and obstetrics characteristics	Descriptive statistics	95% CI
Antenatal obstetric ultrasound, n/N (%)	1695/2113 (80.22)	81.9-78.6
Gestation age at the first obstetric ultrasound, median (IQR)	12 (6.29)	11.85-12.14
Maternal-fetal diseases, n/N (%)	1217/2113 (57.60)	55.6-59.7
Hypertensive disorders, n/N (%)	345/2113 (16.33)	14.8-17.9
Major malformations, n/N (%)	228/2113 (10.79)	9.4-12.2
Diabetes, n/N (%)	179/2113 (8.47)	7.3-9.6
Antenatal infections (eg, HIV, syphilis, toxoplasmosis), n/N (%)	123/2113 (5.82)	4.9-6.8
Nonspontaneous vaginal delivery, n/N (%)	1091/2113 (51.63)	48.2-55.6
Oxytocin augmentation, n/N (%)	375/2113 (17.75)	16.1-19.5
Delivery, cesarean section, n/N (%)	828/2113 (39.19)	37.1-41.3
Delivery, vaginal with forceps, n/N (%)	63/2113 (2.98)	2.3-3.7
Maternal blood transfusion after birth, n/N (%)	45/2113 (2.13)	1.6-2.8
Intensive care unit maternal admission, n/N (%)	24/2098 (1.14)	0.7-1.6
Sex^a^, male, n/N (%)	1098/2113 (51.96)	50.0-54.1
Live birthweight (g), median (IQR)	3055 (690)	3022.5-3085.0
5-minute Apgar score, median (IQR)	9 (1)	9-9
Neonatal resuscitation^b^, n/N (%)	219/2030 (10.79)	9.5-12.2
NICU^c^ admissions, n/N (%)	382/2030 (18.82)	17.1-20.4
NICU admissions^d^, n/N (%)	271/1885 (14.38)	13.3-16.3
Fetal or newborn mortality^e^, n/N (%)	136/2113 (6.44)	5.4-7.6
Fetal or newborn mortality^d,e^, n/N (%)	71/1885 (3.77)	2.9-4.7

^a^Undetermined sex: 8 (0.4%).

^b^Received at least one step of neonatal resuscitation [13].

^c^NICU: neonatal intensive care unit.

^d^Excluding major malformations.

^e^During hospital stay, before or after birth.

Perinatal characteristic comparisons between groups of interest are summarized in Table 2. The group of pregnancies with GA <14 weeks had more antenatal diabetes diagnoses, fewer occurrences of maternal blood transfusion after birth, and fewer NICU admissions, excluding newborns with major malformations, in comparison with the suboptimally dated pregnancy group. The group of pregnancies with a GA of 20 weeks or less and the group with a GA less than 14 weeks had fewer occurrences of maternal blood transfusion after birth when compared with the suboptimally dated pregnancy group.

**Table 2:** Perinatal characteristics according to the quality of gestational age information at birth.

Characteristics	PD^a^ with US^b^<14 weeks (N=1163)	PD with US<20 weeks (N=1456)	Suboptimal PD^c^ (N=657)	*P* value^d^	*P* value^e^
Maternal-fetal diseases, n (%)	684 (58.81)	843 (57.89)	374 (56.93)	.43^f^	.68^f^
Hypertensive disorders, n (%)	202 (17.38)	243 (16.69)	102 (15.53)	.31^f^	.50^f^
Diabetes, n (%)	116 (9.97)	134 (9.2)	45 (6.85)	.02^f^	.07^f^
Maternal blood transfusion after birth, n (%)	17 (1.46)	25 (1.72)	20 (3.04)	.02^f^	.048^f^
ICU^g^ maternal admission, n (%)	12 (1.03)	14 (0.96)	10 (1.52)	.35^f^	.26^f^
5-minute Apgar score, median (IQR)	9 (1)	9 (1)	9 (1)	.64^h^	.47^h^
Live birth weight (g), median (IQR)	3025 (735)	303 (735)	3030 (755)	.79^h^	.70^h^
Major malformations, n (%)	136 (11.69)	162 (11.13)	66 (10.05)	.28^f^	.46^f^
Neonatal resuscitation^i^, n (%)	133 (11.44)	162 (11.13)	62 (9.44)	.19^f^	.24^f^
Neonatal resuscitation^i,j^, n (%)	100 (9.74)	125 (9.66)	51 (8.63)	.46^f^	.48^f^
NICU^k^ admission, n (%)	204 (18.13)	251 (17.85)	131 (20.99)	.15^f^	.095^f^
NICU admission^j^, n (%)	133 (13.25)	168 (13.30)	103 (18.07)	.01^f^	.008^f^
Fetal or neonatal mortality, n (%)	70 (6.02)	89 (6.11)	47 (7.15)	.34^f^	.37^f^
Fetal or newborn mortality^j^, n (%)	32 (3.12)	43 (3.32)	28 (4.74)	.10^f^	.13^f^

^a^PD: pregnancy dating.

^b^US: ultrasound.

^c^Ultrasound done ≥20 weeks of gestation or not done at all.

^d^Comparison between ultrasound dating <14 weeks and suboptimal dating.

^e^Comparison between ultrasound dating <20 weeks and suboptimal dating.

^f^Chi-square test used.

^g^ICU: intensive care unit.

^h^Mann-Whitney test used.

^i^At least one step of neonatal resuscitation [14].

^j^Excluding major malformations.

^k^NICU: neonatal intensive care unit.

### Primary Outcomes

In Table 3, the association between interventions during parturition and the quality of pregnancy dating retrieved at birth is displayed, considering the reference to assist obstetricians in determining GA. Chances of nonspontaneous vaginal delivery were increased by 64% in pregnancies that had the first obstetric ultrasound assessment dating <14 weeks and increased by 58% for ultrasound assessment dating at <20 weeks when compared to the pregnancies with suboptimal dating. Oxytocin augmentation was 41% higher during labor of pregnancies with the first obstetric ultrasound assessment dating <14 weeks and 34% higher for ultrasound assessment dating at <20 weeks in comparison with pregnancies with suboptimal dating. In spite of these results, cesarean section rates and vaginal births with forceps were similar between groups of comparisons.

**Table 3:** Association between the quality of pregnancy dating retrieved at birth and obstetric interventions in labor.

Obstetric intervention during labor	PD^a^ with US^b^ <14 weeks (N=1163), n (%)	PD with US<20 weeks (N=1456), n (%)	Suboptimal PD^c^ (N=657), n (%)	<14 weeks vs suboptimal dating, OR^d^ (95% CI)	*P* value^e^	<20 weeks vs suboptimal dating, OR (95% CI)	*P* value^e^
Nonspontaneous vaginal delivery	652 (56.06)	803 (55.15)	288 (43.84)	1.64 (1.35-1.98)	<.001	1.58 (1.31-1.90)	<.001
Oxytocin augmentation	230 (19.77)	277 (19.03)	98657 (14.92)	1.41 (1.09-1.82)	.01	1.34 (1.04-1.72)	.02
Cesarean section	475 (40.83)	584 (40.11)	244 (37.14)	1.17 (0.96-1.42)	.12	1.13 (0.94-1.37)	.20
Vaginal birth with forceps	42 (3.61)	46 (3.16)	17 (2.59)	0.71 (0.40-1.26)	.24	1.23 (0.69-2.16)	.47

^a^PD: pregnancy dating.

^b^US: ultrasound.

^c^Ultrasound done ≥20 weeks of gestation or not done at all.

^d^OR: odds ratio.

^e^Chi-square test.

## Discussion

### Main Findings

This study underlines how the quality of GA information is associated with timely obstetric interventions at birth. The main finding was that some aspects in the management of childbirth were significantly distinct according to the available information used to calculate GA at birth. We observed an association between assurances of GA estimates at birth by early ultrasound and nonspontaneous vaginal deliveries, as well as an increased proportion of oxytocin augmentation, without affecting cesarean section rates or the frequency of vaginal delivery with forceps. Higher incidence of neonatal NICU admissions, excluding major fetal malformations, indicated the tendency towards more freedom in NICU admissions when pregnancies were suboptimally dated at birth (Table 2). Moreover, no differences were observed regarding the 5-minute Apgar score or birth weight.

Medical choices and women’s parturition preferences are complex, and caregivers try to combine the best practices and available data to achieve the best estimate of GA possible to support clinical decisions [15]. Nonetheless, early ultrasound-based GA is presumed to reduce inductions for postterm pregnancies [6]. Ultrasound examinations are used to confirm the date of the last menstrual period or to assign the due date of birth. However, obtaining adequate references for pregnancy dating remains a challenge in clinical practice and has immediate impact on pregnancy outcomes and direct influence on the accuracy of worldwide prematurity rates, diagnoses of small-for-gestational-age newborns, and perinatal outcomes [5,7,16,17].

### Comparison With Prior Work

Previous studies have described the relationship between the reliability of GA and obstetric and neonatal outcomes. Higher risk of maternal death in pregnant women with unreliable vs reliable last menstrual periods (OR 2.0, 95% CI 1.5-2.6) and a higher risk of stillbirth (OR 2.7, 95% CI 1.7-4.3) were reported by Nguyen et al (2000) [5]. Although our analysis did not include maternal death, pregnancies suboptimally dated with ultrasound assessments at more than 20 weeks or without any ultrasound presented a higher frequency of maternal blood transfusion after birth, even with more spontaneous labors. We interpret this association based on the assumption that the timing for appropriate discontinuation of pregnancy was lost in this group, and the severe hemorrhage morbidity, requiring a blood transfusion after birth, could be in part due to unplanned interruptions in risk situations. This outcome deserves more attention considering severe maternal morbidity and maternal near miss concepts [18]. These necessary details are not available in our database for such an analysis; however, this hypothesis deserves future prospective evaluation.

Our results were consistent with previous evidence, showing that different moments of access to ultrasound facilities during prenatal care are associated with perinatal effects [15,17]. However, no difference was found in labor interventions for pregnancies for the first obstetric ultrasound assessment at 20 weeks or earlier of gestation and the subgroup of the first obstetric ultrasound assessment less than 14 weeks when compared with pregnancies suboptimally dated. In the present analysis, both were statistically associated with the same outcome variables. This result corroborated recommendations that a single ultrasound in the second trimester can be used to estimate GA with reasonable accuracy [2,19]. The WHO recently reported that ultrasound exams before 24 weeks of gestation is the gold standard for the estimation of chronology [2]. Moreover, inadequate pregnancy dating is related to limited early access to prenatal care facilities [7], explaining some of the worst obstetric outcomes in such scenarios [15].

Timely and effective care at birth is one of the most challenging aspects of health care worldwide. Undoubtedly, inaccurate GA is an essential topic in low- and middle-income countries [4]. Achieving lower worldwide prematurity rates is one of the goals established in sustainable development to ensure healthy lives and reduce infant mortality [2], a target that requires feasible strategies based on credible pregnancy dating. Part of the issue is due to the inequities of health facilities worldwide [19], insufficient professional training [7], and the lack of governmental commitment to investing in health care systems [20]. Complications during pregnancy and childbirth affect healthy women populations and is dependent on the inequities of health care facilities [21]. Efforts have been joined to compensate imprecise or unknown GA at birth, such as mathematical models derived from a combination of neonatal screening values [22], mixes of antenatal clinical measurements with obstetric ultrasound in any trimester [7], and emerging low-cost technologies for the assessment of neonatal skin maturity [23]. Moreover, electronic medical records that support clinical routines can benefit patients with safe and accessible information when necessary for better health care results [24].

### Limitations

Our exploratory study is subject to limitations. The risk of recall bias was low because birth data were collected prospectively. However, outcome variables from antenatal care, such as maternal-fetal diseases, were retrieved from a computerized medical database at birth. Therefore, the precise criteria for diagnoses, such as the frequency of gestational diabetes, recorded from the clinical database at birth in the suboptimally dated pregnancies were unable to be met. The diagnosis of diabetes, mainly when occurring for the first time during pregnancy, depends on the interpretation of the screening test when pregnancy, maternal comorbidities, and differences among detection protocols [25].

Another key point in this aspect is the quality of the ultrasonography. Ultrasound offers clinicians a method to estimate GA with high accuracy and precision. Furthermore, effects of ultrasound pregnancy dating on neonatal morbidity, analyzed by Kullinger et al (2016) [15], shows that early differences in fetal growth do in fact exist, as do differences in gender, showing clinical importance when the gestational length is estimated at birth. At our referral health care unit, 1122/2113 (53.1%) of all recorded births received outpatient antenatal care, calling for public and private attention from both our city or neighboring small towns. We believe that future analyses are still warranted to provide a complete and accurate picture of the impact on labor management.

Finally, this study is based on a referral hospital; therefore, the results may not be generalizable in lower complexity hospitals. One prior Brazilian nationwide sample pointed out a 61.3% coverage of pregnant women by prenatal ultrasound [9]. This reality is even worse in other developing countries [4,7]. Therefore, the comparability of our results is limited by the standards of antenatal obstetric care that were similar to our scenario of study.

### Conclusions

A GA of high quality available at birth, assisted by early obstetric ultrasound, was associated with a higher rate of labor interventions for pregnancy interruption, however with lesser maternal blood transfusion and NICU admissions. This study’s analysis highlighted relevant points of health care to improve obstetric care and achieve lower maternal and neonatal morbidity at birth, which confirms the importance of early access to technologies for pregnancy dating as an essential component of quality antenatal care.

## Appendix

Multimedia Appendix 1 Database.

## Abbreviations

GA: gestational age
IQR: interquartile range
NICU: neonatal intensive care unit
OR: odds ratio
WHO: World Health Organization
