# Supplementary material for: Quality of Pregnancy Dating and Obstetric Interventions During Labor: Retrospective Database Analysis
Source: JMIR Pediatr Parent. 2020 Apr 15;3(1):e14109. doi: 10.2196/14109 (PMC7191349; doi:10.2196/14109)
Supplement: Multimedia Appendix 1 [file pediatrics_v3i1e14109_app1.pdf]

C-SECTION	YES	NO	MALE	8	9	3040	NO	NO	NO	NO	YES	NO	NO	NO	NO	NO	14,00	NO	NO	NO	
C-SECTION	YES	NO	MALE	9	9	3050	NO	NO	YES	NO	YES	NO	NO	NO	NO	NO	14,00	NO	NO	NO	
C-SECTION	NO	NO	FEMALE	9	10	3455	NO	NO	NO	NO	YES	NO	NO	NO	NO	NO	14,00	NO	NO	NO	
C-SECTION	YES	NO	MALE	9	10	2975	NO	YES	NO	NO	YES	NO	NO	NO	NO	NO	14,00	NO	NO	NO	
VAGINAL BIRTH		YES	NO	FEMALE	9	10	3555	NO	NO	NO	NO	YES	NO	NO	NO	NO	NO	14,00	NO	NO	YES
VAGINAL BIRTH		YES	NO	MALE	7	9	2340	NO	YES	NO	NO	YES	NO	NO	NO	NO	NO	14,14	NO	NO	NO
C-SECTION	YES	NO	MALE	8	10	3680	NO	NO	NO	NO	YES	NO	NO	NO	NO	NO	14,14	NO	YES	NO	
VAGINAL BIRTH		NO	NO	FEMALE	8	10	3610	NO	NO	NO	NO	YES	NO	NO	NO	NO	NO	14,14	NO	NO	NO
C-SECTION	NO	NO	FEMALE	8	9	4400	NO	NO	NO	NO	YES			NO	NO	NO	14,14	NO	NO	NO	
VAGINAL BIRTH		YES	NO	FEMALE	9	10	2905	NO	NO	NO	NO	YES	NO	NO	NO	YES	NO	14,14	NO	NO	NO
VAGINAL BIRTH		YES	NO	MALE	8	9	2690	NO	NO	NO	NO	YES	NO	NO	NO	NO	NO	14,14	NO	NO	YES
VAGINAL BIRTH		NO	NO	FEMALE	9	9	3300	NO	NO	NO	NO	YES	NO	NO	NO	NO	NO	14,14	NO	NO	YES
VAGINAL BIRTH		YES	NO	FEMALE	9	9	2070	NO	YES	NO	NO	YES	NO	YES	NO	NO	NO	14,29	NO	NO	NO
VAGINAL BIRTH		NO	NO	FEMALE	9	9	3720	NO	NO	NO	NO	YES	NO	NO	NO	NO	NO	14,29	NO	YES	NO
VAGINAL BIRTH		NO	NO	MALE	7	8	2935	NO	NO	NO	NO	YES	NO	NO	NO	NO	NO	14,29	NO	NO	NO
C-SECTION	NO	NO	MALE	8	9	3365	NO	NO	NO	NO	YES	NO	NO	NO	NO	NO	14,29	NO	NO	NO	
VAGINAL BIRTH		NO	NO	FEMALE	9	10	3520	NO	NO	NO	NO	YES	NO	NO	NO	NO	NO	14,29	NO	YES	YES
VAGINAL BIRTH		NO	NO	MALE	9	10	3115	NO	NO	NO	NO	YES	NO	NO	NO	NO	NO	14,29	NO	YES	YES
VAGINAL BIRTH		NO	NO	FEMALE	9	9	2475	NO	NO	NO	NO	YES	NO	NO	NO	YES	NO	14,43	NO	NO	NO
VAGINAL BIRTH		YES	NO	MALE	8	9	4255	NO	NO	NO	NO	YES	NO	NO	NO	NO	NO	14,43	NO	YES	NO
VAGINAL BIRTH		YES	NO	FEMALE	9	10	2945	NO	NO	NO	NO	YES	NO	NO	NO	NO	NO	14,43	NO	NO	NO
VAGINAL BIRTH		YES	NO	MALE	9	10	2915	NO	YES	NO	NO	YES	NO	NO	NO	NO	NO	14,43	NO	NO	NO
C-SECTION	YES	NO	FEMALE	9	9	1635	YES	YES	NO	NO	YES	NO	NO	NO	NO	NO	14,43	NO	NO	NO	
C-SECTION	NO	NO	FEMALE	8	10	2925	NO	NO	NO	NO	YES	NO	NO	NO	NO	NO	14,43	NO	NO	NO	
C-SECTION	YES	NO	MALE			280		NO	NO	YES	YES	NO	NO	YES	NO	NO	14,43	NO	NO	NO	
C-SECTION	YES	NO	MALE	9	10	2220	NO	NO	NO	YES	YES	NO	NO	YES	NO	NO	14,43	NO	NO	NO	
C-SECTION	YES	NO	MALE	6	8	2700	YES	NO	YES	NO	YES	NO	NO	NO	NO	YES	14,43	NO	NO	NO	
VAGINAL BIRTH		YES	NO	MALE	4	6	3815	YES	YES	NO	NO	YES	NO	YES	NO	NO	YES	14,43	NO	YES	YES
VAGINAL BIRTH		YES	NO	MALE	8	9	2190	NO	NO	NO	YES	YES	NO	NO	NO	NO	NO	14,43	NO	NO	YES
VAGINAL BIRTH		YES	NO	MALE	8	9	2285	NO	NO	NO	YES	YES	NO	NO	NO	NO	NO	14,43	NO	NO	YES
VAGINAL BIRTH		NO	NO	MALE	9	10	2725	NO	NO	NO	NO	YES	NO	NO	NO	NO	NO	14,43	NO	NO	YES
VAGINAL BIRTH		NO	NO	MALE	9	9	2880	NO	NO	NO	NO	YES	NO	NO	NO	NO	NO	14,43	NO	NO	YES
VAGINAL BIRTH		NO	NO	FEMALE	9	10	3035	NO	NO	NO	NO	YES	NO	NO	NO	YES	NO	14,43	NO	NO	YES
VAGINAL BIRTH		NO	NO	MALE	9	10	3680	NO	NO	NO	NO	YES	NO	NO	NO	NO	NO	14,43	NO	YES	YES
VAGINAL BIRTH		NO	NO	MALE	8	9	3260	NO	NO	NO	NO	YES	NO	NO	NO	NO	NO	14,43	NO	NO	YES
VAGINAL BIRTH		YES	NO	FEMALE	9	10	2585	NO	YES	YES	NO	YES	NO	NO	NO	NO	NO	14,43	NO	NO	YES
VAGINAL BIRTH		YES	NO	FEMALE	8	9	2710	NO	NO	NO	NO	YES	NO	NO	NO	YES	NO	14,57	NO	NO	NO
VAGINAL BIRTH		NO	NO	FEMALE	8	9	3775	NO	NO	NO	NO	YES	NO	NO	NO	NO	NO	14,57	NO	NO	NO
VAGINAL BIRTH		NO	NO	MALE	8	8	3775	NO	NO	NO	NO	YES	NO	NO	NO	NO	NO	14,57	NO	YES	NO
VAGINAL BIRTH		YES	NO	FEMALE			1035		YES	NO	NO	YES	NO	NO	YES	NO	NO	14,57	NO	NO	NO
C-SECTION	YES	NO	FEMALE	2	9	2780	NO	NO	NO	NO	YES	NO	NO	NO	NO	YES	14,57	NO	NO	NO	
C-SECTION	YES	NO	FEMALE	9	9	3095	NO	NO	NO	YES	YES	NO	NO	NO	NO	NO	14,57	NO	NO	NO	
C-SECTION	YES	NO	FEMALE	8	9	2890	NO	NO	NO	YES	YES	NO	NO	NO	NO	NO	14,57	NO	NO	NO	
C-SECTION	YES	NO	FEMALE	8	9	1530	YES	NO	NO	NO	YES	NO	NO	NO	NO	NO	14,57	NO	NO	YES	
VAGINAL BIRTH		NO	NO	FEMALE	9	10	3425	NO	NO	NO	NO	YES	NO	NO	NO	NO	NO	14,57	NO	YES	YES
VAGINAL BIRTH		YES	NO	MALE	6	8	3240	YES	NO	YES	NO	YES	NO	NO	NO	NO	YES	14,57	NO	NO	YES
C-SECTION	NO	NO	FEMALE	9	9	3685	NO	NO	NO	NO	YES	NO	NO	NO	NO	NO	14,71	NO	NO	NO	
C-SECTION	NO	NO	FEMALE	8	9	3285	NO	NO	NO	NO	YES	NO	NO	NO	NO	NO	14,71	NO	NO	NO	
C-SECTION	NO	NO	MALE	8	9	3325	NO	NO	NO	NO	YES	NO	NO	NO	NO	NO	14,71	NO	NO	NO	
C-SECTION	YES	NO	FEMALE	8	9	2045	NO	YES	NO	YES	YES	NO	NO	NO	NO	NO	14,71	NO	NO	NO	
C-SECTION	YES	NO	FEMALE	9	9	2290	NO	YES	NO	YES	YES	NO	NO	NO	NO	NO	14,71	NO	NO	NO	
VAGINAL BIRTH		YES	NO	MALE	10	10	3370	NO	NO	NO	NO	YES	NO	NO	NO	YES	NO	14,71	NO	NO	YES
VAGINAL BIRTH		YES	NO	FEMALE	9	10	2890	NO	NO	NO	NO	YES	NO	NO	NO	NO	NO	14,71	NO	NO	YES

VAGINAL BIRTH	NO	NO	FEMALE	9	10	3435	NO	NO	NO	NO	YES	NO	NO	NO	NO	NO	14,71	NO	YES	YES
VAGINAL BIRTH	NO	NO	FEMALE	8	10	2880	NO	NO	NO	NO	YES	NO	NO	NO	NO	NO	14,71	NO	NO	YES
VAGINAL BIRTH	NO	NO	MALE	9	9	3390	NO	NO	NO	NO	YES	NO	NO	NO	NO	NO	14,71	NO	NO	YES
VAGINAL BIRTH	NO	NO	MALE	9	10	3600	NO	NO	NO	NO	YES	NO	NO	NO	YES	NO	14,71	NO	NO	YES
C-SECTIONYES	NO	FEMALE	9	9	3605	NO	YES	NO	NO	YES	NO	NO	NO	NO	NO	14,86	NO	NO	NO	
C-SECTIONNO	NO	MALE	9	10	3400	NO	NO	NO	NO	YES	NO	NO	NO	NO	NO	14,86	NO	NO	NO	
C-SECTIONNO	NO	MALE	8	9	3995	YES	NO	NO	NO	YES	NO	NO	NO	NO	NO	14,86	NO	NO	NO	
VAGINAL BIRTH	NO	NO	FEMALE	9	9	3050	NO	NO	NO	NO	YES	NO	NO	NO	NO	NO	14,86	NO	NO	YES
VAGINAL BIRTH	NO	NO	FEMALE	9	10	2890	NO	NO	NO	NO	YES	NO	NO	NO	NO	NO	14,86	NO	NO	YES
VAGINAL BIRTH	NO	NO	FEMALE	10	10	2765	NO	NO	NO	NO	YES	NO	NO	NO	NO	NO	14,86	NO	NO	YES
VAGINAL BIRTH	NO	NO	MALE	8	9	3451	NO	NO	NO	NO	YES	NO	NO	NO	NO	NO	14,86	NO	NO	YES
VAGINAL BIRTH	YES	NO	MALE	8	9	2065	YES	NO	NO	YES	YES	NO	NO	NO	NO	NO	14,86	NO	NO	YES
VAGINAL BIRTH	YES	NO	MALE	8	9	2325	NO	NO	NO	YES	YES	NO	NO	NO	NO	NO	14,86	NO	NO	YES
C-SECTIONYES	NO	MALE	8	9	2750	NO	YES	NO	NO	YES	NO	NO	NO	NO	NO	15,00	NO	NO	NO	
C-SECTIONYES	NO	MALE	1	7	3350	YES	YES	YES	NO	YES	NO	NO	NO	NO	YES	15,00	NO	NO	NO	
C-SECTIONNO	NO	MALE	3	6	2925	YES	NO	NO	NO	YES	NO	NO	NO	NO	YES	15,00	NO	NO	YES	
VAGINAL BIRTH	NO	NO	MALE	10	10	3345	NO	NO	NO	NO	YES	NO	NO	NO	NO	NO	15,00	NO	NO	YES
VAGINAL BIRTH	NO	NO	FEMALE	9	10	2755	YES	NO	NO	NO	YES	NO	NO	NO	NO	NO	15,00	NO	YES	YES
VAGINAL BIRTH	NO	YES	MALE	8	9	2905	NO	NO	NO	NO	YES	NO	NO	NO	NO	NO	15,00	NO	NO	YES
VAGINAL BIRTH	NO	NO	FEMALE	9	9	3160	NO	NO	NO	NO	YES	NO	NO	NO	NO	NO	15,00	NO	NO	YES
VAGINAL BIRTH	NO	NO	MALE	0	0	3420		NO	NO	NO	YES	NO	NO	YES	NO	NO	15,14	NO	YES	NO
C-SECTIONYES	NO	FEMALE	9	9	2650	NO	NO	NO	NO	YES	NO	NO	NO	NO	NO	15,14	NO	NO	NO	
C-SECTIONYES	NO	FEMALE	8	9	2985	NO	NO	NO	NO	YES	NO	NO	NO	YES	NO	15,14	NO	NO	NO	
VAGINAL BIRTH	YES	NO	FEMALE	9	10	3050	NO	NO	NO	NO	YES	NO	NO	NO	YES	NO	15,14	NO	NO	YES
VAGINAL BIRTH	NO	NO	FEMALE	8	9	3165	NO	NO	NO	NO	YES	NO	NO	NO	NO	NO	15,14	NO	NO	YES
VAGINAL BIRTH	NO	NO	FEMALE	8	9	3200	NO	NO	NO	NO	YES	NO	NO	NO	NO	NO	15,14	NO	NO	YES
C-SECTIONYES	NO	MALE	3	7	960	YES	YES	NO	NO	YES	NO	NO	NO	NO	YES	15,29	NO	NO	NO	
VAGINAL BIRTH	NO	NO	MALE	9	10	3615	NO	NO	NO	NO	YES	NO	NO	NO	NO	NO	15,29	NO	NO	YES
VAGINAL BIRTH	NO	NO	MALE	9	10	3295	NO	NO	NO	NO	YES	NO	NO	NO	NO	NO	15,29	NO	NO	YES
VAGINAL BIRTH	NO	NO	FEMALE	9	10	3800	NO	NO	NO	NO	YES	NO	NO	NO	NO	NO	15,29	NO	YES	YES
VAGINAL BIRTH	YES	NO	MALE	5	9	3325	NO	NO	YES	NO	YES	NO	NO	NO	NO	YES	15,43	NO	NO	NO
VAGINAL BIRTH	NO	NO	FEMALE	8	9	3265	NO	NO	NO	NO	YES	NO	NO	NO	NO	NO	15,43	NO	NO	NO
C-SECTIONYES	NO	MALE	9	9	2230	NO	YES	NO	NO	YES	NO	NO	NO	NO	NO	15,43	NO	NO	NO	
VAGINAL BIRTH	YES	NO	MALE	5	8	3250	NO	NO	NO	NO	YES	NO	NO	NO	YES	YES	15,43	NO	YES	NO
VAGINAL BIRTH	NO	NO	FEMALE	9	9	3305	NO	NO	NO	NO	YES	NO	NO	NO	NO	NO	15,43	NO	NO	YES
VAGINAL BIRTH	YES	NO	MALE	9	10	2880	NO	NO	NO	NO	YES	NO	NO	NO	NO	NO	15,43	NO	NO	YES
VAGINAL BIRTH	NO	NO	FEMALE	9	10	2945	NO	NO	NO	NO	YES	NO	NO	NO	NO	NO	15,43	NO	NO	YES
C-SECTIONYES	NO	MALE	9	10	2078	NO	NO	YES	NO	YES	NO	NO	NO	NO	NO	15,57	NO	NO	NO	
VAGINAL BIRTH	YES	NO	MALE	9	9	3125	NO	YES	NO	NO	YES	NO	NO	NO	NO	NO	15,57	NO	NO	NO
VAGINAL BIRTH	YES	NO		0	0			NO	YES	NO	YES	NO	NO	NO	YES	NO	15,57	NO	YES	NO
VAGINAL BIRTH	YES	NO	MALE	9	10	2720	NO	YES	NO	NO	YES	NO	NO	NO	NO	NO	15,57	NO	NO	NO
C-SECTIONYES	NO	MALE	8	8	3590	NO	YES	NO	NO	YES	NO	NO	NO	NO	NO	15,57	NO	NO	NO	
VAGINAL BIRTH	YES	NO	MALE	7	10	1755	YES	NO	NO	NO	YES	NO	NO	NO	YES	NO	15,57	NO	NO	YES
VAGINAL BIRTH	NO	NO	FEMALE	9	10	2910	NO	NO	NO	NO	YES	NO	NO	NO	NO	NO	15,57	NO	NO	YES
VAGINAL BIRTH	NO	NO	FEMALE	9	10	2830	NO	NO	NO	NO	YES	NO	NO	NO	NO	NO	15,57	NO	NO	YES
C-SECTIONNO	NO	MALE	8	9	3465	NO	NO	NO	NO	YES	NO	NO	NO	NO	NO	15,57	NO	NO	YES	
VAGINAL BIRTH	YES	NO	MALE	8	9	3520	NO	NO	NO	NO	YES	NO	NO	NO	NO	NO	15,71	NO	NO	NO
VAGINAL BIRTH	NO	NO	MALE	9	9	3285	NO	NO	NO	NO	YES	NO	NO	NO	NO	NO	15,71	NO	NO	NO
C-SECTIONYES	NO	MALE	5	9	3585	YES	YES	NO	NO	YES	NO	NO	NO	NO	YES	15,71	NO	YES	NO	
C-SECTIONYES	NO	MALE	8	8	2775	NO	NO	NO	NO	YES	NO	NO	NO	NO	NO	15,71	NO	NO	NO	
C-SECTIONNO	NO	FEMALE	9	10	3250	NO	NO	NO	NO	YES	NO	NO	NO	NO	NO	15,71	NO	NO	NO	
C-SECTIONYES	NO	MALE	9	9	3265	NO	NO	YES	NO	YES	NO	NO	NO	NO	NO	15,71	NO	NO	NO	
VAGINAL BIRTH	NO	NO	MALE	8	10	3585	NO	NO	NO	NO	YES	NO	NO	NO	NO	NO	15,71	NO	NO	YES

VAGINAL BIRTH	NO	NO	FEMALE	9	9	3140	NO	NO	NO	NO	YES	NO	NO	NO	NO	NO	15,71	NO	NO	YES
VAGINAL BIRTH	YES	NO	FEMALE	8	9	3175	NO	NO	NO	NO	YES	NO	NO	NO	NO	NO	15,86	NO	YES	NO
C-SECTIONNO	NO	MALE	9	10	3325	NO	NO	NO	NO	YES	NO	NO	NO	NO	NO	15,86	NO	NO	NO	
C-SECTIONYES	NO	FEMALE	8	9	3310	NO	NO	NO	NO	YES	NO	NO	NO	YES	NO	15,86	NO	NO	NO	
C-SECTIONNO	NO	FEMALE	8	9	3040	NO	NO	NO	NO	YES	NO	NO	NO	NO	NO	15,86	NO	NO	NO	
C-SECTIONNO	NO	MALE	9	10	2590	NO	NO	NO	NO	YES	NO	NO	NO	NO	NO	15,86	NO	NO	NO	
VAGINAL BIRTH	YES	NO	MALE	8	9	1910	YES	NO	NO	NO	YES	NO	NO	NO	NO	NO	15,86	NO	NO	YES
VAGINAL BIRTH	NO	NO	MALE	7	9	3470	NO	NO	NO	NO	YES	NO	NO	NO	NO	NO	15,86	NO	NO	YES
VAGINAL BIRTH	YES	NO	FEMALE	9	9	2930	NO	YES	NO	NO	YES	NO	NO	NO	NO	NO	16,00	NO	NO	NO
C-SECTIONNO	NO	MALE	7	9	3665	YES	NO	NO	NO	YES	NO	NO	NO	NO	NO	16,00	NO	NO	NO	
VAGINAL BIRTH	YES	NO	FEMALE	9	9	2935	NO	YES	NO	NO	YES	NO	NO	NO	NO	NO	16,00	NO	NO	NO
VAGINAL BIRTH	NO	NO	MALE	8	10	3665	NO	NO	NO	NO	YES	NO	NO	NO	NO	NO	16,00	NO	NO	YES
VAGINAL BIRTH	NO	NO	MALE	9	9	3560	NO	NO	NO	NO	YES	NO	NO	NO	NO	NO	16,00	NO	NO	YES
VAGINAL BIRTH	YES	NO	FEMALE	9	10	3080	NO	YES	NO	NO	YES	NO	NO	NO	NO	NO	16,14	NO	NO	NO
C-SECTIONYES	NO	FEMALE	9	10	3750	NO	NO	YES	NO	YES	NO	NO	NO	NO	NO	16,14	NO	NO	NO	
VAGINAL BIRTH	YES	NO	FEMALE	8	9	1565	YES	NO	NO	NO	YES	NO	NO	NO	NO	NO	16,14	NO	NO	YES
VAGINAL BIRTH	YES	NO	FEMALE	9	10	2770	NO	NO	NO	YES	YES	NO	NO	NO	NO	NO	16,14	NO	YES	YES
VAGINAL BIRTH	NO	NO	FEMALE	9	10	2935	NO	NO	NO	NO	YES	NO	NO	NO	NO	NO	16,14	NO	NO	YES
VAGINAL BIRTH	YES	NO	MALE			450		NO	NO	NO	YES	NO	NO	YES	NO	NO	16,14	NO	NO	YES
VAGINAL BIRTH	NO	NO	FEMALE	9	10	3525	NO	NO	NO	NO	YES	NO	NO	NO	NO	NO	16,14	NO	NO	YES
C-SECTIONYES	NO	MALE	8	9	3305	NO	NO	NO	NO	YES	NO	NO	NO	YES	NO	16,14	NO	NO	YES	
VAGINAL BIRTH	YES	NO	FEMALE	9	10	3310	NO	NO	YES	NO	YES	NO	NO	NO	NO	NO	16,29	NO	YES	NO
VAGINAL BIRTH	YES	NO	MALE	8	9	3325	NO	NO	YES	NO	YES	NO	NO	NO	NO	NO	16,29	NO	NO	NO
VAGINAL BIRTH	YES	NO	FEMALE	7	8	2960	NO	NO	YES	NO	YES	NO	NO	NO	NO	NO	16,29	NO	YES	NO
VAGINAL BIRTH	NO	NO	FEMALE	8	8	2665	NO	NO	NO	NO	YES	NO	NO	NO	NO	NO	16,29	NO	YES	NO
C-SECTIONYES	NO	FEMALE	8	9	3310	NO	NO	NO	NO	YES	NO	NO	NO	NO	NO	16,29	NO	NO	NO	
C-SECTIONYES	NO	MALE	9	9	3505	NO	NO	NO	NO	YES	NO	NO	NO	NO	NO	16,29	NO	NO	NO	
VAGINAL BIRTH	YES	NO	FEMALE	8	9	1775	YES	NO	NO	NO	YES	NO	NO	NO	NO	NO	16,29	NO	YES	YES
VAGINAL BIRTH	YES	NO	FEMALE	9	10	3400	NO	YES	NO	NO	YES	NO	NO	NO	NO	NO	16,29	NO	NO	YES
VAGINAL BIRTH	NO	NO	MALE	9	10	2820	NO	NO	NO	NO	YES	NO	NO	NO	NO	NO	16,43	NO	NO	NO
VAGINAL BIRTH	YES	NO	FEMALE	9	9	3090	NO	YES	NO	NO	YES	NO	NO	NO	NO	NO	16,43	NO	NO	NO
C-SECTIONYES	NO	MALE	4	8	3000	YES	YES	NO	NO	YES	NO	NO	NO	NO	YES	16,43	NO	NO	NO	
C-SECTIONNO	NO	FEMALE	9	9	3070	NO	NO	NO	NO	YES	NO	NO	NO	NO	NO	16,43	NO	NO	NO	
VAGINAL BIRTH	YES	NO	MALE	9	9	3575	NO	NO	NO	NO	YES	NO	NO	NO	NO	NO	16,43	NO	NO	YES
VAGINAL BIRTH	NO	NO	FEMALE	8	9	3070	NO	NO	NO	NO	YES	NO	NO	NO	NO	NO	16,43	NO	NO	YES
VAGINAL BIRTH	NO	NO	FEMALE	9	10	3900	NO	NO	NO	NO	YES	NO	NO	NO	NO	NO	16,43	NO	NO	YES
VAGINAL BIRTH	NO	NO	MALE	8	9	3195	NO	NO	NO	NO	YES	NO	NO	NO	NO	NO	16,43	NO	NO	YES
C-SECTIONNO	NO	MALE	4	9	3590	NO	NO	NO	NO	YES	NO	NO	NO	NO	YES	16,57	NO	NO	NO	
VAGINAL BIRTH	NO	NO	FEMALE	9	10	3390	NO	NO	NO	NO	YES	NO	NO	NO	NO	NO	16,57	NO	YES	YES
VAGINAL BIRTH	NO	NO	FEMALE	9	10	3690	NO	NO	NO	NO	YES	NO	NO	NO	NO	NO	16,57	NO	NO	YES
VAGINAL BIRTH	NO	NO	MALE	9	10	3020	NO	NO	NO	NO	YES	NO	NO	NO	NO	NO	16,57	NO	NO	YES
VAGINAL BIRTH	NO	NO	FEMALE	9	9	2855	NO	NO	NO	NO	YES	NO	NO	NO	NO	NO	16,57	NO	NO	YES
C-SECTIONNO	NO	FEMALE	3	7	3445	NO	NO	NO	NO	YES	NO	NO	NO	NO	YES	16,71	NO	NO	NO	
C-SECTIONNO	NO	MALE	7	9	3225	NO	NO	NO	NO	YES	NO	NO	NO	NO	NO	16,71	NO	NO	NO	
C-SECTIONNO	NO	FEMALE	9	9	3125	NO	NO	NO	NO	YES	NO	NO	NO	NO	NO	16,71	NO	NO	NO	
VAGINAL BIRTH	YES	NO	FEMALE	9	10	2555	NO	NO	NO	NO	YES	NO	NO	NO	NO	NO	16,71	NO	YES	YES
VAGINAL BIRTH	YES	NO	MALE	0	5	2275	YES	NO	NO	NO	YES	NO	NO	NO	NO	YES	16,71	NO	NO	YES
VAGINAL BIRTH	NO	NO	MALE	9	9	3715	NO	NO	NO	NO	YES	NO	NO	NO	NO	NO	16,71	NO	NO	YES
C-SECTIONYES	NO	MALE	9	9	4010	NO	NO	YES	NO	YES	NO	NO	NO	NO	NO	16,86	NO	NO	NO	
C-SECTIONYES	NO	MALE	9	9	2215	NO	NO	NO	NO	YES	NO	NO	NO	YES	NO	16,86	NO	NO	NO	
VAGINAL BIRTH	YES	NO	FEMALE	9	9	3100	NO	NO	NO	NO	YES	NO	NO	NO	YES	NO	16,86	NO	NO	YES
VAGINAL BIRTH	YES	NO	MALE	9	10	1870	YES	NO	NO	NO	YES	NO	NO	NO	NO	NO	16,86	NO	NO	YES
VAGINAL BIRTH	YES	NO	FEMALE	6	9	2790	NO	NO	NO	NO	YES	NO	NO	NO	YES	YES	16,86	NO	NO	YES

C-SECTION	NO	NO	MALE	9	10	2920	YES	NO	NO	NO	YES	NO	NO	NO	NO	NO	17,00	NO	NO	NO	
VAGINAL BIRTH		NO	YES	MALE	3	9	3415	YES	NO	NO	NO	YES	YES	NO	NO	NO	YES	17,00	NO	YES	YES
C-SECTION	YES	NO	MALE	5	10	1180	YES	NO	NO	YES	YES	NO	YES	NO	NO	YES	17,00	NO	NO	YES	
C-SECTION	YES	NO	MALE	7	9	1555	NO	NO	NO	YES	YES	NO	YES	NO	NO	NO	17,00	NO	NO	YES	
C-SECTION	NO	NO	FEMALE	9	9	2870	NO	NO	NO	NO	YES	NO	NO	NO	NO	NO	17,00	NO	NO	YES	
VAGINAL BIRTH		NO	NO	FEMALE	9	9	3150	NO	NO	NO	NO	YES	NO	NO	NO	NO	NO	17,00	NO	NO	YES
VAGINAL BIRTH		YES	NO	FEMALE	9	9	3510	NO	NO	YES	NO	YES	NO	NO	NO	NO	NO	17,14	NO	YES	NO
VAGINAL BIRTH		NO	NO	MALE	8	9	3295	NO	NO	NO	NO	YES	NO	NO	NO	NO	NO	17,14	NO	YES	NO
C-SECTION	YES	NO	MALE	9	10	1955	NO	NO	NO	NO	YES	NO	NO	NO	NO	NO	17,14	NO	NO	NO	
VAGINAL BIRTH		NO	NO	FEMALE	8	9	4130	NO	NO	NO	NO	YES	NO	NO	NO	NO	NO	17,14	NO	NO	YES
VAGINAL BIRTH		YES	NO	MALE	10	10	2775	NO	NO	NO	NO	YES	NO	NO	NO	NO	NO	17,14	NO	NO	YES
C-SECTION	NO	NO	FEMALE	5	8	2480	YES	NO	NO	NO	YES	NO	NO	NO	NO	YES	17,14	NO	NO	YES	
VAGINAL BIRTH		NO	NO	MALE	9	10	3680	NO	NO	NO	NO	YES	NO	NO	NO	NO	NO	17,14	NO	YES	YES
VAGINAL BIRTH		YES	NO	MALE	8	9	3260	NO	NO	YES	NO	YES	NO	NO	NO	NO	NO	17,29	NO	NO	NO
C-SECTION	NO	NO	MALE	7	8	3030	YES	NO	NO	NO	YES	NO	NO	NO	NO	NO	17,29	NO	NO	YES	
VAGINAL BIRTH		NO	YES	MALE	9	9	3020	NO	NO	NO	NO	YES	NO	NO	NO	NO	NO	17,29	NO	YES	YES
C-SECTION	YES	NO	FEMALE	9	10	2510	NO	NO	NO	NO	YES	NO	NO	NO	NO	NO	17,43	NO	NO	NO	
C-SECTION	NO	NO	FEMALE	9	10	3360	NO	NO	NO	NO	YES	NO	NO	NO	NO	NO	17,43	NO	NO	NO	
C-SECTION	YES	NO	MALE	8	10	3965	YES	NO	NO	NO	YES	NO	NO	NO	NO	NO	17,43	NO	NO	NO	
C-SECTION	YES	NO	FEMALE	8	8	3290	NO	NO	NO	NO	YES	NO	NO	NO	NO	NO	17,43	NO	NO	NO	
VAGINAL BIRTH		NO	NO	FEMALE	8	9	3345	NO	NO	NO	NO	YES	NO	NO	NO	NO	NO	17,43	NO	NO	YES
VAGINAL BIRTH		YES	NO	MALE	0	0	495		NO	NO	NO	YES	NO	NO	YES	NO	NO	17,43	NO	NO	YES
VAGINAL BIRTH		YES	NO	FEMALE	9	10	2190	NO	YES	NO	NO	YES	NO	NO	NO	NO	NO	17,43	NO	NO	YES
C-SECTION	NO	NO	FEMALE	9	9	3470	NO	NO	NO	NO	YES	NO	NO	NO	NO	NO	17,43	NO	YES	YES	
VAGINAL BIRTH		NO	NO	FEMALE	9	10	3120	NO	NO	NO	NO	YES	NO	NO	NO	NO	NO	17,43	NO	NO	YES
VAGINAL BIRTH		YES	NO	MALE	8	9	3045	NO	NO	NO	NO	YES	NO	NO	NO	NO	NO	17,43	NO	NO	YES
C-SECTION	NO	NO	MALE	8	9	3345	NO	NO	NO	NO	YES	NO	NO	NO	NO	NO	17,43	NO	NO	YES	
VAGINAL BIRTH		NO	NO	MALE	9	9	3440	NO	NO	NO	NO	YES	NO	NO	NO	NO	NO	17,43	NO	NO	YES
VAGINAL BIRTH		NO	NO	MALE	9	10	3205	NO	NO	NO	NO	YES	NO	NO	NO	NO	NO	17,43	NO	NO	YES
VAGINAL BIRTH		NO	NO	FEMALE	9	9	3090	NO	NO	NO	NO	YES	NO	NO	NO	NO	NO	17,43	NO	NO	YES
VAGINAL BIRTH		NO	YES	FEMALE	4	7	2810	YES	NO	NO	NO	YES			NO	NO	YES	17,57	NO	YES	NO
C-SECTION	YES	NO	FEMALE	10	10	2985	NO	NO	NO	NO	YES	NO	NO	NO	YES	NO	17,57	NO	NO	NO	
VAGINAL BIRTH		YES	NO	MALE	9	9	2505	NO	NO	NO	NO	YES	NO	NO	NO	NO	NO	17,57	NO	YES	YES
VAGINAL BIRTH		NO	NO	FEMALE	9	10	2595	NO	NO	NO	NO	YES	NO	NO	NO	NO	NO	17,57	NO	NO	YES
C-SECTION	NO	NO	FEMALE	8	9	2840	NO	NO	NO	NO	YES	NO	NO	NO	NO	NO	17,57	NO	NO	YES	
VAGINAL BIRTH		NO	NO	FEMALE	9	9	2555	NO	NO	NO	NO	YES	NO	NO	NO	NO	NO	17,57	NO	NO	YES
C-SECTION	YES	NO	FEMALE	8	9	2470	NO	NO	NO	NO	YES	NO	NO	NO	NO	NO	17,71	NO	YES	NO	
C-SECTION	YES	NO	FEMALE	9	9	2690	NO	NO	NO	NO	YES	NO	NO	NO	YES	NO	17,71	NO	NO	NO	
VAGINAL BIRTH		YES	NO	FEMALE	9	9	2565	NO	NO	NO	NO	YES	NO	NO	NO	YES	NO	17,71	NO	NO	NO
C-SECTION	NO	NO	MALE	9	10	3565	NO	NO	NO	NO	YES	NO	NO	NO	NO	NO	17,71	NO	NO	NO	
C-SECTION	NO	NO	MALE	4	7	3295	YES	NO	NO	NO	YES	NO	NO	NO	NO	YES	17,71	NO	NO	NO	
C-SECTION	YES	NO	FEMALE	8	8	2365	YES	NO	NO	NO	YES	NO	NO	NO	NO	NO	17,71	NO	NO	NO	
C-SECTION	YES	NO	MALE	8	8	4175	NO	NO	NO	NO	YES	NO	NO	NO	NO	NO	17,71	NO	NO	NO	
VAGINAL BIRTH		NO	NO	MALE	9	9	2940	NO	NO	NO	NO	YES	NO	NO	NO	NO	NO	17,71	NO	YES	YES
VAGINAL BIRTH		NO	NO	MALE	9	10	3340	NO	NO	NO	NO	YES	NO	NO	NO	NO	NO	17,71	NO	NO	YES
C-SECTION	NO	NO	MALE	8	9	3390	NO	NO	NO	NO	YES	NO	NO	NO	NO	NO	17,86	NO	NO	NO	
VAGINAL BIRTH		YES	NO	MALE	9	10	2395	NO	YES	NO	NO	YES	NO	NO	NO	NO	NO	17,86	NO	NO	NO
VAGINAL BIRTH		NO	NO	MALE	9	9	2560	NO	NO	NO	NO	YES	NO	NO	NO	NO	NO	17,86	NO	YES	YES
C-SECTION	YES	NO	MALE	9	10	3755	NO	NO	NO	NO	YES	NO	NO	NO	NO	NO	18,00	NO	NO	NO	
C-SECTION	NO	NO	FEMALE	9	10	3345	NO	NO	NO	NO	YES	NO	NO	NO	NO	NO	18,00	NO	NO	NO	
VAGINAL BIRTH		YES	NO	MALE	9	9	3480	NO	YES	NO	NO	YES	NO	YES	NO	NO	NO	18,00	NO	NO	YES
C-SECTION	NO	NO	FEMALE	6	9	3600	NO	NO	NO	NO	YES	NO	NO	NO	NO	YES	18,00	NO	YES	YES	
VAGINAL BIRTH		YES	NO	FEMALE	9	10	2680	NO	NO	NO	NO	YES	NO	NO	NO	NO	NO	18,00	NO	NO	YES

VAGINAL BIRTH	YES	NO	MALE	0	0	1890		NO	NO	NO	YES	NO	NO	YES	NO	NO	18,14	NO	NO	NO
VAGINAL BIRTH	NO	NO	FEMALE	8	10	2890	NO	NO	NO	NO	YES	NO	NO	NO	NO	NO	18,14	NO	NO	YES
C-SECTIONYES	NO	MALE	8	8	2610	NO	YES	NO	NO	YES	NO	YES	NO	NO	NO	18,29	NO	NO	NO	
C-SECTIONYES	NO	MALE	9	10	2748	NO	NO	NO	NO	YES	NO	NO	NO	NO	NO	18,29	NO	NO	NO	
VAGINAL BIRTH	NO	NO	MALE	8	8	3125	NO	NO	NO	NO	YES	NO	NO	NO	NO	NO	18,29	NO	NO	YES
VAGINAL BIRTH	NO	NO	MALE	8	9	4070	NO	NO	NO	NO	YES	NO	NO	NO	NO	NO	18,29	NO	NO	YES
VAGINAL BIRTH	YES	NO	MALE	9	10	2870	NO	NO	NO	YES	YES	NO	NO	NO	NO	NO	18,29	NO	NO	YES
VAGINAL BIRTH	YES	NO	MALE	9	10	2765	NO	NO	NO	YES	YES	NO	NO	NO	NO	NO	18,29	NO	NO	YES
VAGINAL BIRTH	NO	NO	MALE	9	10	3435	NO	NO	NO	NO	YES	NO	NO	NO	NO	NO	18,29	NO	YES	YES
C-SECTIONYES	NO	MALE	8	9	2615	NO	YES	NO	NO	YES	NO	NO	NO	NO	NO	18,43	NO	NO	NO	
VAGINAL BIRTH	YES	NO	FEMALE	9	10	3280	NO	YES	NO	NO	YES	NO	NO	NO	NO	NO	18,43	NO	NO	NO
VAGINAL BIRTH	YES	NO	FEMALE	8	9	3135	NO	NO	NO	NO	YES	NO	NO	NO	NO	NO	18,43	NO	NO	NO
C-SECTIONYES	NO	MALE	1	2	2670	YES	NO	NO	NO	YES	NO	NO	NO	NO	YES	18,43	NO	NO	NO	
C-SECTIONNO	NO	MALE	7	8	3515	NO	NO	NO	NO	YES	NO	NO	NO	NO	NO	18,43	NO	NO	NO	
VAGINAL BIRTH	YES	NO	FEMALE	7	9	3380	NO	NO	NO	NO	YES	NO	NO	NO	NO	NO	18,43	NO	NO	YES
VAGINAL BIRTH	NO	NO	FEMALE	9	10	3110	NO	NO	NO	NO	YES	NO	NO	NO	NO	NO	18,43	NO	NO	YES
VAGINAL BIRTH	NO	NO	FEMALE	8	9	3025	NO	NO	NO	NO	YES	NO	NO	NO	NO	NO	18,43	NO	NO	YES
VAGINAL BIRTH	NO	NO	MALE	8	9	3330	NO	NO	NO	NO	YES	NO	NO	NO	NO	NO	18,43	NO	NO	YES
VAGINAL BIRTH	NO	NO	MALE	9	10	3115	NO	NO	NO	NO	YES	NO	NO	NO	NO	NO	18,43	NO	NO	YES
VAGINAL BIRTH	YES	NO	FEMALE	9	10	2530	NO	YES	NO	NO	YES	NO	NO	NO	NO	NO	18,57	NO	NO	NO
VAGINAL BIRTH	NO	NO	MALE	6	8	3360	YES	NO	NO	NO	YES	NO	YES	NO	NO	YES	18,57	NO	YES	YES
VAGINAL BIRTH	NO	NO	FEMALE	9	9	3270	NO	NO	NO	NO	YES	NO	NO	NO	NO	NO	18,71	NO	NO	NO
VAGINAL BIRTH	NO	NO	FEMALE	9	10	2915	NO	NO	NO	NO	YES	NO	NO	NO	NO	NO	18,71	NO	YES	NO
VAGINAL BIRTH	YES	NO	FEMALE	0	0	380	NO	NO	NO	NO	YES	NO	NO	YES	NO	NO	18,71	NO	YES	YES
C-SECTIONNO	NO	MALE	8	9	2845	NO	NO	NO	NO	YES	NO	NO	NO	NO	NO	18,86	NO	NO	NO	
VAGINAL BIRTH	YES	NO	MALE	0	0	280		NO	NO	NO	YES	NO	NO	YES	NO	NO	18,86	NO	NO	NO
C-SECTIONYES	NO	MALE	8	10	3360	NO	NO	NO	NO	YES	NO	NO	NO	YES	NO	18,86	NO	NO	NO	
VAGINAL BIRTH	NO	NO	FEMALE	9	9	3240	NO	NO	NO	NO	YES	NO	NO	NO	NO	NO	18,86	NO	NO	YES
VAGINAL BIRTH	YES	NO	MALE	9	10	3140	NO	YES	NO	NO	YES	NO	NO	NO	NO	NO	18,86	NO	NO	YES
VAGINAL BIRTH	NO	NO	FEMALE	9	10	2820	NO	NO	NO	NO	YES	NO	NO	NO	NO	NO	19,00	NO	NO	NO
C-SECTIONYES	NO	MALE	2	6	2710	YES	NO	NO	NO	YES	NO	NO	NO	NO	YES	19,00	NO	NO	YES	
C-SECTIONYES	NO	FEMALE	7	9	2980	YES	YES	NO	NO	YES	YES	YES	NO	NO	NO	19,14	NO	YES	NO	
C-SECTIONNO	NO	FEMALE	9	10	2820	NO	NO	NO	NO	YES	NO	NO	NO	NO	NO	19,14	NO	NO	NO	
C-SECTIONNO	NO	MALE	9	10	3755	NO	NO	NO	NO	YES	NO	NO	NO	NO	NO	19,14	NO	NO	NO	
VAGINAL BIRTH	NO	NO	FEMALE	9	10	2980	NO	NO	NO	NO	YES	NO	NO	NO	NO	NO	19,14	NO	NO	YES
C-SECTIONYES	NO	FEMALE	8	9	3250	NO	YES	NO	NO	YES	NO	NO	NO	NO	NO	19,29	NO	NO	NO	
C-SECTIONNO	NO	FEMALE	9	9	3215	NO	NO	NO	NO	YES	NO	NO	NO	NO	NO	19,29	NO	NO	NO	
VAGINAL BIRTH	NO	NO	MALE	9	10	3780	NO	NO	NO	NO	YES	NO	NO	NO	NO	NO	19,29	NO	NO	YES
VAGINAL BIRTH	NO	NO	FEMALE	8	9	2845	NO	NO	NO	NO	YES	NO	NO	NO	NO	NO	19,29	NO	YES	YES
VAGINAL BIRTH	YES	NO	FEMALE	9	9	2455	NO	NO	NO	NO	YES	NO	NO	NO	YES	NO	19,43	NO	YES	NO
VAGINAL BIRTH	YES	NO	MALE	9	9	3015	NO	NO	NO	NO	YES	NO	NO	NO	NO	NO	19,43	NO	NO	NO
C-SECTIONNO	NO	MALE	9	9	2485	NO	NO	NO	NO	YES	NO	NO	NO	NO	NO	19,43	NO	NO	NO	
C-SECTIONYES	NO	MALE	9	10	2140	NO	YES	NO	NO	YES	NO	NO	NO	NO	NO	19,43	NO	NO	NO	
C-SECTIONYES	NO	MALE	8	9	2850	NO	NO	NO	NO	YES	NO	NO	NO	YES	NO	19,43	NO	NO	NO	
VAGINAL BIRTH	YES	NO	FEMALE	9	10	3090	NO	NO	NO	NO	YES	NO	NO	NO	NO	NO	19,43	NO	NO	YES
VAGINAL BIRTH	YES	NO	FEMALE	8	9	3595	YES	NO	YES	NO	YES	NO	NO	NO	NO	NO	19,43	NO	NO	YES
VAGINAL BIRTH	NO	NO	FEMALE	8	10	3775	NO	NO	NO	NO	YES	NO	NO	NO	NO	NO	19,43	NO	NO	YES
VAGINAL BIRTH	YES	NO	FEMALE	1	1	290	NO	NO	NO	NO	YES	NO	NO	YES	NO	NO	19,43	NO	NO	YES
C-SECTIONYES	NO	FEMALE	8	9	3160	NO	YES	NO	NO	YES	NO	NO	NO	NO	NO	19,57	NO	NO	NO	
C-SECTIONYES	NO	FEMALE	8	9	3035	NO	YES	NO	NO	YES	NO	NO	NO	NO	NO	19,57	NO	NO	NO	
VAGINAL BIRTH	NO	NO	FEMALE	9	9	3065	NO	NO	NO	NO	YES	NO	NO	NO	NO	NO	19,57	NO	NO	YES
VAGINAL BIRTH	NO	NO	FEMALE	3	3	2800	NO	NO	NO	NO	YES	NO	NO	YES	NO	YES	19,57	NO	NO	YES
VAGINAL BIRTH	YES	NO	MALE	8	9	2880	NO	NO	NO	NO	YES	NO	NO	NO	NO	NO	19,71	NO	NO	NO

VAGINAL BIRTH		NO	NO	MALE	9	9	3535	YES	NO	NO	NO	YES			NO	NO	NO	19,71	NO	NO	YES
VAGINAL BIRTH		NO	NO	FEMALE	8	9	3625	NO	NO	NO	NO	YES	NO	NO	NO	NO	NO	19,86	NO	NO	YES
VAGINAL BIRTH		YES	NO	FEMALE	7	8	3300	YES	NO	NO	NO	YES	NO	NO	NO	NO	YES		NO	NO	NO
C-SECTION	NO	NO	MALE	9	10	3230	NO	NO	NO	NO	YES	NO	NO	NO	NO	NO		NO	NO	NO	
C-SECTION	YES	NO	MALE	9	10	3015	NO	NO	NO	NO	YES	NO	NO	NO	YES	NO		NO	YES	YES	
VAGINAL BIRTH		NO	YES	FEMALE	8	9	3900	NO	NO	NO	NO	YES	NO	NO	NO	NO	NO		NO	YES	YES
VAGINAL BIRTH		YES	NO	FEMALE	9	10	3160	NO	NO	NO	NO	YES	NO	NO	NO	NO	NO		NO	NO	YES
VAGINAL BIRTH		NO	NO	MALE	8	10	2565	NO	NO	NO	NO	YES	NO	NO	NO	NO	NO		NO	YES	YES
C-SECTION	NO	NO	FEMALE	8	9	4185	NO	NO	NO	NO	YES	NO	NO	NO	NO	NO		NO	NO	YES	
VAGINAL BIRTH		NO	NO	MALE	9	10	3100	NO	NO	NO	NO	YES	NO	NO	NO	NO	NO		NO	NO	YES
C-SECTION	YES	NO	FEMALE	2	4	3265	YES	YES	NO	NO	YES	NO	NO	NO	NO	YES		NO	NO	YES	
C-SECTION	NO	NO	FEMALE	9	9	2830	NO	NO	NO	NO	YES	NO	NO	NO	NO	NO		NO	YES	YES	
C-SECTION	YES	NO	FEMALE	8	8	2975	NO	NO	NO	NO	YES	NO	NO	NO	NO	NO		NO	NO	NO	
VAGINAL BIRTH		YES	NO	FEMALE	9	9	3230	NO	NO	NO	NO	YES	NO	NO	NO	NO	NO		NO	YES	YES
VAGINAL BIRTH		NO	NO	MALE	8	8	3990	NO	NO	NO	NO	YES	NO	NO	NO	NO	NO		NO	NO	YES
C-SECTION	NO	NO	MALE	9	10	3875	NO	NO	NO	NO	YES	NO	NO	NO	NO	NO		NO	NO	NO	
C-SECTION	YES	NO	MALE	9	9	3145	NO	NO	NO	NO	YES	NO	NO	NO	NO	NO		NO	NO	NO	
C-SECTION	YES	NO	FEMALE	8	9	3505	NO	NO	NO	NO	YES	NO	NO	NO	NO	NO		NO	NO	NO	
VAGINAL BIRTH		NO	YES	FEMALE	9	10	3170	NO	NO	NO	NO	YES	NO	NO	NO	NO	NO		NO	YES	YES
VAGINAL BIRTH		NO	NO	MALE	8	10	3305	NO	NO	NO	NO	YES	NO	NO	NO	NO	NO		NO	NO	YES
C-SECTION	NO	NO	MALE	9	10	3320	NO	NO	NO	NO	YES	NO	NO	NO	NO	NO		NO	NO	NO	
C-SECTION	NO	NO	FEMALE	9	10	2770	NO	NO	NO	NO	YES	NO	NO	NO	NO	NO		NO	NO	YES	
VAGINAL BIRTH		NO	NO	MALE	8	10	3595	NO	NO	NO	NO	YES	NO	NO	NO	NO	NO		NO	NO	YES
VAGINAL BIRTH		NO	NO	MALE	9	9	2710	NO	NO	NO	NO	YES	NO	NO	NO	NO	NO		NO	NO	NO
VAGINAL BIRTH		NO	NO	MALE	9	10	2840	NO	NO	NO	NO	YES	NO	NO	NO	NO	NO		NO	NO	YES
VAGINAL BIRTH		NO	NO	MALE	10	10	2675	NO	NO	NO	NO	YES	NO	NO	NO	NO	NO		NO	NO	YES
VAGINAL BIRTH		NO	NO	FEMALE	9	10	2600	NO	NO	NO	NO	YES	NO	NO	NO	NO	NO		NO	NO	YES
VAGINAL BIRTH		NO	NO	MALE	5	7	2580	NO	NO	NO	NO	YES	NO	NO	NO	NO	YES		NO	NO	NO
C-SECTION	YES	NO	FEMALE	8	9	3940	NO	YES	YES	NO	YES	NO	NO	NO	NO	NO		NO	NO	NO	
C-SECTION	YES	NO	FEMALE	5	9	2740	YES	YES	NO	NO	YES	YES	YES	NO	NO	YES		NO	NO	NO	
C-SECTION	YES	NO	FEMALE	8	9	2430	NO	NO	NO	NO	YES	NO	NO	NO	NO	NO		NO	NO	NO	
C-SECTION	YES	NO	MALE	8	9	3540	NO	NO	YES	NO	YES	NO	NO	NO	NO	NO		NO	NO	NO	
C-SECTION	YES	NO	FEMALE	8	7	2375	YES	NO	NO	NO	YES	NO	NO	NO	NO	NO		NO	NO	YES	
VAGINAL BIRTH		NO	NO	FEMALE	8	9	2840	NO	NO	NO	NO	YES	NO	NO	NO	NO	NO		NO	NO	YES
VAGINAL BIRTH		YES	NO	FEMALE	8	9	3355	NO	YES	NO	NO	YES	NO	NO	NO	NO	NO		NO	NO	NO
C-SECTION	YES	NO	MALE	9	9	3820	NO	YES	NO	NO	YES	NO	NO	NO	NO	NO		NO	NO	NO	
VAGINAL BIRTH		NO	NO	MALE	4	8	3435	YES	NO	NO	NO	YES	NO	NO	NO	NO	YES		NO	YES	YES
VAGINAL BIRTH		YES	NO	MALE	1	1	520	NO	YES	NO	NO	YES	NO	NO	YES	NO	YES		NO	NO	YES
VAGINAL BIRTH		NO	NO	FEMALE	8	10	3215	NO	NO	NO	NO	YES	NO	NO	NO	NO	NO		NO	NO	YES
C-SECTION	YES	NO	FEMALE			2030		NO	NO	NO	YES	YES	YES	YES	NO	NO		NO	NO	NO	
C-SECTION	YES	NO	MALE	9	10	3135	NO	YES	NO	NO	YES	NO	NO	NO	YES	NO		NO	NO	NO	
C-SECTION	YES	NO	MALE	6	8	2490	NO	YES	NO	NO	YES	NO	NO	NO	NO	YES		NO	NO	NO	
VAGINAL BIRTH		NO	NO	MALE	9	10	3015	NO	NO	NO	NO	YES	NO	NO	NO	NO	NO		NO	NO	YES
VAGINAL BIRTH		YES	NO	FEMALE	8	9	3430	NO	NO	NO	NO	YES	NO	NO	NO	NO	NO		NO	YES	YES
VAGINAL BIRTH		NO	NO	MALE	8	9	3310	NO	NO	NO	NO	YES	NO	NO	NO	NO	NO		NO	NO	YES
VAGINAL BIRTH		NO	NO	FEMALE	8	9	2560	NO	NO	NO	NO	YES	NO	NO	NO	NO	NO		NO	NO	NO
C-SECTION	YES	NO	MALE	9	9	3680	NO	YES	NO	NO	YES	NO	NO	NO	NO	NO		NO	YES	NO	
C-SECTION	NO	NO	MALE	9	9	3290	NO	NO	NO	NO	YES	NO	NO	NO	NO	NO		NO	NO	NO	
C-SECTION	YES	NO	FEMALE	6	7	3265	NO	NO	YES	NO	YES	NO	NO	NO	NO	YES		NO	NO	YES	
C-SECTION	YES	NO	MALE	8	9	1425	YES	YES	NO	NO	YES	NO	NO	NO	NO	NO		NO	NO	NO	
C-SECTION	NO	NO	MALE	7	9	3115	NO	NO	NO	NO	YES	NO	NO	NO	NO	NO		NO	NO	YES	
C-SECTION	NO	NO	MALE	8	9	2755	NO	NO	NO	NO	YES	NO	NO	NO	NO	NO		NO	NO	YES	
VAGINAL BIRTH		NO	NO	MALE	8	10	3400	NO	NO	NO	NO	YES	NO	NO	NO	NO	NO		NO	NO	YES

C-SECTION	YES	NO	MALE	4	7	2285	NO	NO	NO	NO	YES	NO	NO	NO	YES	YES		NO	NO	NO	
VAGINAL BIRTH		YES	NO	MALE	10	10	2825	NO	NO	NO	NO	YES	NO	NO	NO	NO	NO		NO	YES	NO
VAGINAL BIRTH		YES	NO	MALE	9	10	2245	NO	NO	NO	NO	YES	NO	NO	NO	NO	NO		NO	YES	NO
C-SECTION	YES	NO	MALE	0	0	4750		YES	YES	NO	NO	NO	NO	YES	NO	NO		NO	NO	YES	
C-SECTION	NO	NO	FEMALE	9	10	3360	NO	NO	NO	NO	NO	NO	NO	NO	NO	NO		NO	NO	YES	
C-SECTION	YES	NO	MALE	0	0	1940		YES	NO	NO	NO	NO	NO	YES	NO	NO		NO	NO	YES	
C-SECTION	YES	NO	MALE	3	5	2515	YES	YES	YES	NO	NO	YES	YES	NO	NO	YES		NO	NO	NO	
VAGINAL BIRTH		YES	NO	MALE	8	9	3125	NO	NO	NO	NO	NO	NO	YES	NO	NO	NO		NO	YES	NO
VAGINAL BIRTH		YES	NO	MALE	8	9	3240	NO	YES	NO	NO	NO	NO	NO	NO	NO	NO		NO	NO	NO
VAGINAL BIRTH		NO	NO	MALE	8	9	4575	NO	NO	NO	NO	YES	NO	NO	NO	NO	NO		NO	NO	NO
C-SECTION	YES	NO	FEMALE	9	9	3725	NO	NO	YES	NO	YES	NO	NO	NO	NO	NO		NO	NO	NO	
C-SECTION	YES	NO	MALE	5	8	1095	YES	NO	YES	NO	YES	NO	NO	NO	NO	YES		NO	NO	NO	
VAGINAL BIRTH		YES	NO	FEMALE			1610		NO	NO	NO	YES	NO	NO	YES	YES	NO		NO	NO	NO
VAGINAL BIRTH		YES	NO	FEMALE	0	0	265		YES	NO	NO	YES	NO	NO	YES	NO	NO		NO	NO	NO
VAGINAL BIRTH		YES	NO	MALE	0	0	285		YES	YES	NO	YES	NO	NO	YES	NO	NO		NO	NO	NO
C-SECTION	YES	NO	MALE	9	9	2065	NO	YES	NO	NO	YES	NO	NO	NO	NO	NO		NO	NO	NO	
C-SECTION	NO	NO	FEMALE	9	10	3365	NO	NO	NO	NO	YES	NO	NO	NO	NO	NO		NO	YES	NO	
VAGINAL BIRTH		NO	NO	FEMALE	10	10	3080	NO	NO	NO	NO	YES	NO	NO	NO	NO	NO		NO	NO	NO
C-SECTION	YES	NO	FEMALE	9	10	3700	NO	NO	NO	NO	NO	NO	NO	NO	YES	NO		NO	YES	NO	
C-SECTION	NO	NO	FEMALE	9	9	3990	NO	NO	NO	NO	YES	NO	NO	NO	NO	NO		NO	YES	NO	
VAGINAL BIRTH		YES	NO	MALE	4	8	2945	YES	NO	NO	NO	YES	NO	NO	NO	YES	YES		NO	NO	NO
C-SECTION	NO	NO	FEMALE	9	9	3330	NO	NO	NO	NO	YES	NO	NO	NO	NO	NO		NO	NO	NO	
VAGINAL BIRTH		NO	NO	FEMALE	9	10	3135	NO	NO	NO	NO	YES	NO	NO	NO	NO	NO		NO	NO	NO
VAGINAL BIRTH		NO	NO	FEMALE	9	10	2880	NO	NO	NO	NO	YES	NO	NO	NO	NO	NO		NO	NO	NO
VAGINAL BIRTH		YES	NO	MALE	8	9	4705	NO	NO	YES	NO	YES	NO	NO	NO	NO	NO		NO	NO	NO
VAGINAL BIRTH		YES	NO	MALE	8	9	3045	NO	YES	NO	NO	YES	NO	NO	NO	NO	NO		NO	NO	NO
VAGINAL BIRTH		NO	NO	FEMALE	9	10	2840	NO	NO	NO	NO	YES	NO	NO	NO	NO	NO		NO	NO	NO
VAGINAL BIRTH		NO	NO	MALE	8	9	3830	NO	NO	NO	NO	NO	NO	NO	NO	NO	NO		NO	YES	NO
VAGINAL BIRTH		NO	NO	MALE	8	9	3670	NO	NO	NO	NO	YES	NO	NO	NO	NO	NO		NO	YES	NO
VAGINAL BIRTH		YES	NO	MALE	9	10	3390	NO	YES	NO	NO	YES	NO	NO	NO	NO	NO		NO	YES	NO
VAGINAL BIRTH		NO	NO	FEMALE	9	9	2540	NO	NO	NO	NO	YES	NO	NO	NO	NO	NO		NO	NO	NO
VAGINAL BIRTH		YES	NO	MALE	9	9	3575	NO	NO	NO	NO	YES	NO	NO	NO	NO	NO		NO	YES	NO
VAGINAL BIRTH		NO	NO	FEMALE	0	0	2275		NO	NO	NO	YES	NO	NO	YES	NO	NO		NO	NO	NO
VAGINAL BIRTH		YES	NO	FEMALE	9	10	2490	NO	YES	NO	NO	YES	NO	NO	NO	NO	NO		NO	NO	NO
C-SECTION	YES	NO	FEMALE	9	10	2355	NO	NO	NO	NO	NO	NO	NO	NO	NO	NO		NO	NO	NO	
VAGINAL BIRTH		YES	NO	MALE	8	9	2475	YES	NO	NO	NO	NO	NO	NO	NO	NO	NO		NO	NO	NO
VAGINAL BIRTH		YES	NO	FEMALE	8	9	2560	YES	NO	NO	NO	NO	NO	NO	NO	NO	NO		NO	NO	NO
C-SECTION	YES	NO	MALE	9	10	3185	NO	NO	YES	NO	NO	NO	NO	NO	NO	NO		NO	NO	NO	
C-SECTION	YES	NO	MALE	8	9	3795	YES	NO	NO	NO	YES	NO	NO	NO	YES	NO		NO	YES	NO	
VAGINAL BIRTH		YES	NO	FEMALE	9	10	2810	NO	NO	NO	NO	YES	NO	NO	NO	NO	NO		NO	YES	NO
C-SECTION	YES	NO	MALE	8	10	2650	NO	NO	NO	NO	YES	NO	NO	NO	NO	NO		NO	YES	NO	
VAGINAL BIRTH		YES	NO	MALE	7	9	4090	NO	NO	YES	NO	NO	NO	NO	NO	NO	NO		NO	YES	NO
VAGINAL BIRTH		NO	NO	MALE	9	10	3630	YES	NO	NO	NO	YES	NO	NO	NO	NO	NO		NO	YES	NO
VAGINAL BIRTH		NO	NO	FEMALE	9	10	2905	NO	NO	NO	NO	NO	NO	NO	NO	NO	NO		NO	NO	NO
VAGINAL BIRTH		YES	NO	FEMALE	3	8	3785	NO	NO	NO	NO	YES	NO	NO	NO	NO	YES		NO	YES	NO
C-SECTION	NO	NO	FEMALE	9	10	3370	NO	NO	NO	NO	NO	NO	NO	NO	NO	NO		NO	YES	NO	
VAGINAL BIRTH		YES	NO	FEMALE	9	10	2955	NO	YES	NO	NO	NO	NO	NO	NO	NO	NO		NO	YES	NO
VAGINAL BIRTH		NO	NO	MALE	9	10	3320	NO	NO	NO	NO	NO	NO	NO	NO	NO	NO		NO	NO	NO
VAGINAL BIRTH		YES	NO	MALE	9	9	3190	NO	YES	NO	NO	YES	NO	NO	NO	NO	NO		NO	YES	NO
VAGINAL BIRTH		NO	NO	MALE	8	9	3400	NO	NO	NO	NO	YES	NO	NO	NO	NO	NO		NO	YES	NO
C-SECTION	YES	NO	FEMALE	9	9	3670	NO	YES	NO	NO	NO	NO	NO	NO	NO	NO		NO	NO	NO	
VAGINAL BIRTH		NO	NO	FEMALE	8	9	3090	NO	NO	NO	NO	YES	NO	NO	NO	NO	NO		NO	NO	NO
VAGINAL BIRTH		YES	NO	MALE	9	9	2545	NO	NO	NO	NO	YES	NO	NO	NO	NO	NO		NO	NO	NO

VAGINAL BIRTH		YES	NO	MALE	0	0			NO	NO	NO	NO	NO	NO	YES	NO	NO		NO	NO	NO
VAGINAL BIRTH		YES	NO	MALE	8	9	3360	NO	YES	NO	NO	YES	NO	NO	NO	NO	NO		NO	NO	NO
C-SECTION	NO	NO	FEMALE	9	10	3640	NO	NO	NO	NO	YES	NO	NO	NO	NO	NO		NO	YES	NO	
C-SECTION	YES	NO	FEMALE	8	9	2760	NO	YES	NO	NO	NO	NO	NO	NO	NO	NO		NO	NO	NO	
C-SECTION	YES	NO	FEMALE	7	8	3330	NO	NO	NO	NO	YES	NO	NO	NO	YES	NO		NO	NO	NO	
VAGINAL BIRTH		YES	NO	MALE	8	9	3095	NO	NO	NO	NO	NO	NO	NO	NO	NO	NO		NO	NO	NO
C-SECTION	YES	NO	FEMALE	8	9	2690	NO	YES	NO	NO	YES	NO	NO	NO	NO	NO		NO	NO	NO	
VAGINAL BIRTH		YES	NO	MALE	7	9	3205	NO	YES	NO	NO	YES	NO	NO	NO	NO	NO		NO	YES	NO
VAGINAL BIRTH		YES	NO	FEMALE	9	10	3130	NO	NO	YES	NO	YES	NO	NO	NO	NO	NO		NO	NO	NO
VAGINAL BIRTH		YES	NO	MALE	9	9	3160	NO	YES	NO	NO	YES	NO	NO	NO	NO	NO		NO	NO	NO
VAGINAL BIRTH		NO	NO	FEMALE	8	9	3390	NO	NO	NO	NO	YES	NO	NO	NO	NO	NO		NO	NO	NO
VAGINAL BIRTH		YES	NO	FEMALE	9	10	2715	NO	NO	NO	NO	YES	NO	NO	NO	YES	NO		NO	NO	NO
VAGINAL BIRTH		YES	NO	MALE	9	9	2890	NO	NO	NO	NO	YES	NO	NO	NO	NO	NO		NO	YES	NO
C-SECTION	YES	NO	FEMALE	4	6	775	YES	YES	NO	NO	YES	YES	YES	NO	NO	YES		NO	NO	NO	
C-SECTION	YES	NO	MALE	8	9	2600	YES	YES	NO	NO	NO	YES	YES	NO	NO	NO		NO	NO	NO	
C-SECTION	YES	NO	MALE	0	0	350		NO	NO	NO	NO	YES	YES	YES	NO	NO		NO	NO	NO	
C-SECTION	YES	NO	FEMALE	2	4	3300	YES	NO	NO	NO	NO	NO	YES	NO	YES	YES		NO	NO	NO	
C-SECTION	YES	NO	FEMALE	9	10	2985	YES	NO	NO	NO	YES	NO	YES	NO	YES	NO		NO	NO	NO	
VAGINAL BIRTH		YES	NO	FEMALE	0	0	500		NO	NO	NO	NO	NO	NO	YES	NO	NO		NO	NO	NO
C-SECTION	YES	NO	FEMALE	8	9	2350	YES	NO	NO	NO	NO	NO	NO	NO	NO	NO		NO	NO	NO	
C-SECTION	YES	NO	MALE	9	10	2090	YES	NO	NO	NO	NO	NO	NO	NO	NO	NO		NO	NO	NO	
C-SECTION	YES	NO	FEMALE	8	9	1740	YES	YES	NO	NO	NO	NO	NO	NO	NO	NO		NO	NO	NO	
C-SECTION	YES	NO	MALE	9	9	3195	NO	NO	NO	NO	YES	NO	NO	NO	YES	NO		NO	NO	NO	
C-SECTION	NO	NO	FEMALE	8	9	3370	NO	NO	NO	NO	YES	NO	NO	NO	NO	NO		NO	NO	NO	
C-SECTION	YES	NO	FEMALE	9	9	2740	NO	NO	NO	NO	NO	NO	NO	NO	YES	NO		NO	NO	NO	
C-SECTION	YES	NO	FEMALE	8	9	2650	NO	NO	NO	NO	NO	NO	NO	NO	YES	NO		NO	NO	NO	
C-SECTION	YES	NO	FEMALE	9	10	2930	NO	NO	NO	NO	YES	NO	NO	NO	NO	NO		NO	NO	NO	
C-SECTION	YES	NO	MALE	8	9	4085	YES	NO	YES	NO	YES	NO	NO	NO	NO	NO		NO	NO	NO	
C-SECTION	YES	NO	FEMALE	9	10	2500	NO	NO	NO	NO	NO	NO	NO	NO	YES	NO		NO	NO	NO	
C-SECTION	YES	NO	FEMALE	9	9	3365	NO	YES	NO	NO	NO	NO	NO	NO	NO	NO		NO	NO	NO	
C-SECTION	YES	NO	MALE	8	9	2830	NO	NO	NO	NO	YES	NO	NO	NO	NO	NO		NO	NO	NO	
C-SECTION	NO	NO	FEMALE	9	9	3055	NO	NO	NO	NO	NO	NO	NO	NO	NO	NO		NO	NO	NO	
C-SECTION	NO	NO	MALE	8	9	3125	NO	NO	NO	NO	NO	NO	NO	NO	NO	NO		NO	NO	NO	
VAGINAL BIRTH		NO	NO	FEMALE	8	9	3015	NO	NO	NO	NO	YES	NO	NO	NO	NO	NO		NO	NO	NO
C-SECTION	NO	NO	FEMALE	9	10	2915	NO	NO	NO	NO	YES	NO	NO	NO	NO	NO		NO	NO	NO	
C-SECTION	YES	NO	MALE	9	10	3615	NO	YES	NO	NO	YES	NO	NO	NO	NO	NO		NO	NO	NO	
VAGINAL BIRTH		YES	NO	MALE	7	8	935	YES	NO	NO	YES	YES	NO	NO	YES	NO	NO		NO	NO	NO
VAGINAL BIRTH		YES	NO	FEMALE	4	9	785	YES	NO	NO	YES	YES	NO	NO	YES	NO	YES		NO	NO	NO
C-SECTION	YES	NO	MALE	6	9	1570	YES	YES	NO	NO	YES	NO	NO	YES	NO	YES		NO	NO	NO	
C-SECTION	YES	NO	FEMALE	7	9	930	YES	YES	YES	NO	NO	NO	NO	NO	NO	NO		NO	NO	NO	
C-SECTION	YES	NO	FEMALE	8	9	535	YES	YES	NO	NO	YES	NO	NO	NO	NO	NO		NO	NO	NO	
C-SECTION	YES	NO	MALE	9	9	1690	YES	YES	NO	NO	YES	NO	NO	NO	NO	NO		NO	NO	NO	
C-SECTION	YES	NO	MALE	8	9	1930	YES	NO	NO	YES	YES	NO	NO	NO	NO	NO		NO	NO	NO	
C-SECTION	YES	NO	MALE	8	9	925	YES	NO	NO	YES	YES	NO	NO	NO	NO	NO		NO	NO	NO	
C-SECTION	YES	NO	FEMALE	9	9	2300	NO	NO	NO	NO	YES	NO	NO	NO	NO	NO		NO	NO	NO	
VAGINAL BIRTH		YES	NO	MALE	10	9	1735	YES	YES	NO	NO	NO	NO	NO	NO	NO	NO		NO	NO	NO
C-SECTION	YES	NO	FEMALE	8	8	2145	YES	YES	NO	NO	NO	NO	NO	NO	NO	NO		NO	NO	NO	
C-SECTION	YES	NO	FEMALE	8	8	2475	NO	NO	YES	NO	YES	NO	NO	NO	NO	NO		NO	NO	NO	
C-SECTION	YES	NO	MALE	8	10	3710	NO	YES	NO	NO	YES	NO	NO	NO	NO	NO		NO	NO	NO	
C-SECTION	YES	NO	MALE	8	9	2985	NO	YES	YES	NO	NO	NO	NO	NO	NO	NO		NO	NO	NO	
C-SECTION	YES	NO	FEMALE	9	10	3040	NO	YES	NO	NO	YES	NO	NO	NO	NO	NO		NO	NO	NO	
C-SECTION	NO	NO	FEMALE	9	9	3070	NO	NO	NO	NO	YES	NO	NO	NO	NO	NO		NO	NO	NO	
C-SECTION	YES	NO	FEMALE	9	10	3215	NO	NO	NO	NO	NO	NO	NO	NO	NO	NO		NO	NO	NO	

C-SECTION	YES	NO	FEMALE	9	9	2505	NO	NO	NO	NO	NO	NO	NO	NO	NO	NO		NO	NO	NO	
C-SECTION	YES	NO	MALE	8	9	3820	NO	NO	NO	NO	YES	NO	NO	NO	NO	NO		NO	NO	NO	
C-SECTION	YES	NO	FEMALE	8	9	3970	NO	NO	NO	NO	NO	NO	NO	NO	YES	NO		NO	NO	NO	
C-SECTION	YES	NO	FEMALE	8	8	3830	NO	YES	YES	NO	YES	NO	NO	NO	NO	NO		NO	NO	NO	
C-SECTION	YES	NO	MALE	9	9	3175	YES	YES	NO	NO	YES	NO	NO	NO	NO	NO		NO	NO	NO	
C-SECTION	YES	NO	FEMALE	8	8	3245	NO	NO	NO	NO	YES	NO	NO	NO	NO	NO		NO	NO	NO	
C-SECTION	YES	NO	FEMALE	8	8	2620	NO	YES	NO	NO	YES	NO	NO	NO	NO	NO		NO	NO	NO	
C-SECTION	YES	NO	MALE	8	9	3545	NO	NO	NO	NO	NO	NO	NO	NO	NO	NO		NO	NO	NO	
C-SECTION	NO	NO	FEMALE	9	10	2750	NO	NO	NO	NO	YES	NO	NO	NO	NO	NO		NO	NO	NO	
C-SECTION	NO	NO	MALE	9	10	2725	NO	NO	NO	NO	NO	NO	NO	NO	NO	NO		NO	NO	NO	
C-SECTION	NO	NO	MALE	8	9	2775	NO	NO	NO	NO	NO	NO	NO	NO	NO	NO		NO	NO	NO	
C-SECTION	YES	NO	FEMALE	8	9	2985	NO	YES	NO	NO	YES	NO	YES	NO	YES	NO		NO	NO	NO	
C-SECTION	YES	NO	MALE	8	10	4380	NO	NO	YES	NO	YES	NO	YES	NO	NO	NO		NO	NO	NO	
VAGINAL BIRTH		YES	NO	MALE	8	9	2945	NO	NO	NO	NO	YES	NO	NO	NO	NO	NO		NO	NO	NO
C-SECTION	YES	NO	FEMALE	8	9	2770	NO	NO	NO	NO	YES	NO	NO	NO	NO	NO		NO	NO	NO	
C-SECTION	NO	NO	FEMALE	8	9	2990	NO	NO	NO	NO	NO	NO	NO	NO	NO	NO		NO	NO	NO	
C-SECTION	YES	NO	MALE	9	9	3650	NO	NO	YES	NO	NO	NO	NO	NO	NO	NO		NO	NO	NO	
C-SECTION	YES	NO	MALE	8	9	3340	YES	NO	NO	NO	NO	NO	NO	NO	NO	NO		NO	NO	NO	
C-SECTION	YES	NO	MALE	7	9	1435	YES	NO	NO	YES	YES	NO	NO	NO	NO	NO		NO	NO	NO	
C-SECTION	YES	NO	MALE	8	8	1790	YES	NO	NO	YES	YES	NO	NO	NO	NO	NO		NO	NO	NO	
C-SECTION	YES	NO	MALE	7	9	4120	NO	NO	YES	NO	NO	NO	NO	NO	NO	NO		NO	NO	NO	
C-SECTION	YES	NO	FEMALE	6	8	3700	YES	NO	YES	NO	NO	NO	NO	NO	NO	YES		NO	NO	NO	
C-SECTION	YES	NO	MALE	9	9	4245	NO	NO	NO	NO	NO	NO	NO	NO	NO	NO		NO	NO	NO	
C-SECTION	YES	NO	MALE	2	6	4315	NO	NO	NO	NO	YES	NO	NO	NO	NO	YES		NO	NO	NO	
C-SECTION	YES	NO	FEMALE	9	10	3220	NO	YES	NO	NO	NO	NO	NO	NO	NO	NO		NO	NO	NO	
C-SECTION	NO	NO	MALE	9	10	3405	NO	NO	NO	NO	NO	NO	NO	NO	NO	NO		NO	NO	NO	
C-SECTION	YES	NO	MALE	4	9	2540	NO	NO	YES	NO	NO	NO	YES	NO	NO	YES		NO	NO	NO	
C-SECTION	YES	NO	MALE	3	4	1825	YES	NO	NO	NO	NO	NO	YES	NO	NO	YES		NO	NO	NO	
C-SECTION	YES	NO	FEMALE	7	9	1950	YES	YES	NO	NO	YES	YES	NO	NO	NO	NO		NO	NO	NO	
C-SECTION	YES	NO	FEMALE	7	9	1180	YES	NO	NO	NO	NO	NO	NO	NO	NO	NO		NO	NO	NO	
VAGINAL BIRTH		YES	NO	FEMALE	9	10	3405	NO	YES	NO	NO	NO	NO	YES	NO	NO	NO		NO	NO	YES
VAGINAL BIRTH		YES	NO	MALE	8	9	2425	NO	NO	NO	NO	NO	NO	NO	NO	NO	NO		NO	NO	YES
VAGINAL BIRTH		YES	NO	FEMALE	7	8	2310	NO	YES	NO	NO	NO	NO	NO	NO	NO	NO		NO	NO	YES
VAGINAL BIRTH		YES	NO	FEMALE	8	10	3220	YES	NO	NO	NO	NO	NO	NO	NO	YES	NO		NO	NO	YES
C-SECTION	NO	NO	FEMALE	1	4	3800	YES	NO	NO	NO	NO	NO	NO	NO	NO	YES		NO	NO	YES	
VAGINAL BIRTH		NO	NO	FEMALE	9	10	2780	NO	NO	NO	NO	NO	NO	NO	NO	NO	NO		NO	NO	YES
C-SECTION	YES	NO	MALE	6	8	3590	YES	NO	NO	NO	NO	NO	NO	NO	NO	YES		NO	NO	YES	
VAGINAL BIRTH		NO	NO	MALE	10	10	2905	NO	NO	NO	NO	NO	NO	NO	NO	NO	NO		NO	NO	YES
VAGINAL BIRTH		NO	NO	FEMALE	9	9	2880	NO	NO	NO	NO	NO	NO	NO	NO	NO	NO		NO	NO	YES
VAGINAL BIRTH		NO	NO	MALE	9	10	3395	NO	NO	NO	NO	NO	NO	NO	NO	NO	NO		NO	NO	YES
VAGINAL BIRTH		YES	NO	MALE	6	8	2880	NO	NO	NO	NO	NO	NO	NO	NO	YES	YES		NO	NO	YES
C-SECTION	YES	NO	MALE	9	9	2805	NO	NO	NO	NO	NO	NO	NO	NO	NO	NO		NO	NO	YES	
C-SECTION	YES	NO	FEMALE	2	8	1110	YES	NO	NO	NO	NO	NO	NO	NO	NO	NO		NO	NO	YES	
VAGINAL BIRTH		YES	NO	FEMALE	0	0	320		NO	NO	NO	NO	NO		NO	NO	NO		NO	NO	YES
C-SECTION	NO	NO	MALE	3	8	3640	YES	NO	NO	NO	YES	YES	YES	NO	NO	YES		NO	NO	YES	
C-SECTION	YES	NO	MALE	9	9	2120	YES	NO	NO	NO	NO	NO	YES	NO	NO	NO		NO	NO	YES	
VAGINAL BIRTH		YES	NO	MALE	8	8	845	YES	NO	NO	YES	YES	NO	NO	YES	YES	NO		NO	NO	YES
VAGINAL BIRTH		YES	NO	FEMALE	3	7	705	YES	NO	NO	YES	YES	NO	NO	NO	YES	YES		NO	NO	YES
VAGINAL BIRTH		NO	NO	FEMALE	8	9	1290	YES	NO	NO	NO	NO	NO	NO	NO	NO	NO		NO	NO	YES
VAGINAL BIRTH		YES	NO	FEMALE	9	10	2280	NO	NO	NO	NO	YES	NO	NO	NO	NO	NO		NO	YES	YES
VAGINAL BIRTH		NO	NO	FEMALE	8	8	2720	YES	NO	NO	NO	YES	NO	NO	NO	NO	NO		NO	YES	YES
C-SECTION	NO	NO	MALE	9	10	2975	NO	NO	NO	NO	NO	NO	NO	NO	NO	NO		NO	NO	YES	
VAGINAL BIRTH		NO	NO	FEMALE	9	10	3240	NO	NO	NO	NO	YES	NO	NO	NO	NO	NO		NO	YES	YES

VAGINAL BIRTH	YES	NO	MALE	9	10	2985	NO	YES	NO	NO	NO	NO	NO	NO	NO	NO		NO	NO	YES
VAGINAL BIRTH	NO	NO	MALE	9	10	3065	NO	NO	NO	NO	NO	NO	NO	NO	NO	NO		NO	NO	YES
VAGINAL BIRTH	YES	NO	MALE	8	9	3090	NO	NO	NO	NO	YES	NO	NO	NO	NO	NO		NO	NO	YES
VAGINAL BIRTH	NO	NO	FEMALE	9	10	2965	NO	NO	NO	NO	NO	NO	NO	NO	NO	NO		NO	NO	YES
VAGINAL BIRTH	NO	NO	FEMALE	9	9	3450	NO	NO	NO	NO	YES	NO	NO	NO	NO	NO		NO	NO	YES
VAGINAL BIRTH	NO	NO	FEMALE	9	10	3080	NO	NO	NO	NO	NO	NO	NO	NO	NO	NO		NO	YES	YES
VAGINAL BIRTH	YES	NO	MALE	9	9	3035	NO	YES	NO	NO	YES	NO	NO	NO	NO	NO		NO	NO	YES
VAGINAL BIRTH	NO	NO	FEMALE	9	9	3395	NO	NO	NO	NO	NO	NO	NO	NO	NO	NO		NO	NO	YES
VAGINAL BIRTH	NO	NO	MALE	8	10	3725	NO	NO	NO	NO	YES	NO	NO	NO	NO	NO		NO	YES	YES
VAGINAL BIRTH	NO	NO	MALE	8	9	2935	NO	NO	NO	NO	NO	NO	NO	NO	NO	NO		NO	NO	YES
VAGINAL BIRTH	YES	NO	FEMALE	9	10	3405	NO	NO	NO	NO	YES	NO	NO	NO	NO	NO		NO	NO	YES
VAGINAL BIRTH	YES	NO	FEMALE	9	10	3680	NO	NO	NO	NO	YES	NO	NO	NO	NO	NO		NO	NO	YES
VAGINAL BIRTH	YES	NO	MALE	8	10	2760	NO	YES	NO	NO	YES	NO	NO	NO	NO	NO		NO	NO	YES
C-SECTIONNO	NO	MALE	4	8	3395	YES	NO	NO	NO	YES	NO	NO	NO	NO	YES		NO	NO	YES	
VAGINAL BIRTH	NO	NO	MALE	9	10	2970	NO	NO	NO	NO	NO	NO	NO	NO	NO	NO		NO	NO	YES
VAGINAL BIRTH	NO	NO	MALE	9	10	2755	NO	NO	NO	NO	YES	NO	NO	NO	NO	NO		NO	YES	YES
VAGINAL BIRTH	NO	NO	MALE	8	10	3490	NO	NO	NO	NO	YES	NO	NO	NO	NO	NO		NO	NO	YES
VAGINAL BIRTH	NO	NO	FEMALE	10	10	3360	NO	NO	NO	NO	YES	NO	NO	NO	NO	NO		NO	NO	YES
VAGINAL BIRTH	NO	NO	FEMALE	8	9	2685	NO	NO	NO	NO	YES	NO	NO	NO	NO	NO		NO	NO	YES
VAGINAL BIRTH	YES	NO	FEMALE	8	9	3020	NO	YES	NO	NO	NO	NO	NO	NO	NO	NO		NO	YES	YES
VAGINAL BIRTH	YES	NO	MALE	8	9	3715	NO	NO	NO	NO	NO	NO	NO	NO	NO	NO		NO	YES	YES
VAGINAL BIRTH	YES	NO	MALE	10	10	3260	NO	YES	NO	NO	YES	NO	NO	NO	NO	NO		NO	NO	YES
C-SECTIONNO	NO	MALE	6	10	3210	NO	NO	NO	NO	YES	NO	NO	NO	NO	NO		NO	NO	YES	
VAGINAL BIRTH	NO	NO	FEMALE	4	8	2700	NO	NO	NO	NO	YES	NO	NO	NO	NO	YES		NO	NO	YES
VAGINAL BIRTH	NO	YES	MALE	8	10	3370	YES	NO	NO	NO	YES	NO	NO	NO	NO	NO		NO	YES	YES
VAGINAL BIRTH	NO	NO	MALE	9	10	3480	NO	NO	NO	NO	YES	NO	NO	NO	NO	NO		NO	YES	YES
VAGINAL BIRTH	NO	NO	FEMALE	9	9	3010	NO	NO	NO	NO	YES	NO	NO	NO	NO	NO		NO	NO	YES
VAGINAL BIRTH	NO	NO	FEMALE	9	10	2765	NO	NO	NO	NO	NO	NO	NO	NO	NO	NO		NO	YES	YES
VAGINAL BIRTH	YES	NO	MALE	9	10	3305	NO	NO	NO	NO	NO	NO	NO	NO	YES	NO		NO	NO	YES
VAGINAL BIRTH	NO	NO	FEMALE	8	9	3200	NO	NO	NO	NO	YES	NO	NO	NO	NO	NO		NO	NO	YES
VAGINAL BIRTH	YES	NO	FEMALE	8	8	3195	NO	NO	NO	NO	NO	NO	NO	NO	NO	NO		NO	NO	YES
C-SECTIONNO	NO	FEMALE	8	9	3570	NO	NO	NO	NO	NO	NO	NO	NO	NO	NO		NO	NO	YES	
VAGINAL BIRTH	NO	NO	FEMALE	8	9	3690	NO	NO	NO	NO	YES	NO	NO	NO	NO	NO		NO	NO	YES
VAGINAL BIRTH	NO	NO	MALE	8	9	2840	NO	NO	NO	NO	YES	NO	NO	NO	NO	NO		NO	YES	YES
VAGINAL BIRTH	NO	NO	FEMALE	9	9	3200	NO	NO	NO	NO	NO	NO	NO	NO	NO	NO		NO	NO	YES
VAGINAL BIRTH	NO	NO	MALE	7	9	2830	NO	NO	NO	NO	YES	NO	NO	NO	NO	NO		NO	NO	YES
VAGINAL BIRTH	NO	NO	FEMALE	9	10	3270	NO	NO	NO	NO	YES	NO	NO	NO	NO	NO		NO	NO	YES
VAGINAL BIRTH	NO	NO	MALE	9	10	2785	NO	NO	NO	NO	YES	NO	NO	NO	NO	NO		NO	NO	YES
VAGINAL BIRTH	NO	NO	FEMALE	9	10	2915	NO	NO	NO	NO	YES	NO	NO	NO	NO	NO		NO	YES	YES
VAGINAL BIRTH	NO	NO	MALE	8	9	2605	NO	NO	NO	NO	YES	NO	NO	NO	NO	NO		NO	NO	YES
VAGINAL BIRTH	NO	YES	MALE	9	10	2990	NO	NO	NO	NO	YES	NO	NO	NO	NO	NO		NO	NO	YES
VAGINAL BIRTH	NO	NO	FEMALE	8	9	3005	NO	NO	NO	NO	YES	NO	NO	NO	NO	NO		NO	NO	YES
VAGINAL BIRTH	NO	YES	MALE	9	10	3875	NO	NO	NO	NO	NO	NO	NO	NO	NO	NO		NO	NO	YES
VAGINAL BIRTH	NO	NO	FEMALE	10	10	3120	NO	NO	NO	NO	NO	NO	NO	NO	NO	NO		NO	NO	YES
VAGINAL BIRTH	NO	NO	FEMALE	8	8	2610	NO	NO	NO	NO	YES	NO	NO	NO	NO	NO		NO	NO	YES
VAGINAL BIRTH	NO	NO	FEMALE	6	9	3700	YES	NO	NO	NO	NO	NO	NO	NO	NO	YES		NO	NO	YES
VAGINAL BIRTH	YES	NO	MALE	8	8	3460	NO	NO	NO	NO	NO	NO	NO	NO	NO	NO		NO	YES	YES
VAGINAL BIRTH	NO	YES	FEMALE	9	10	2995	NO	NO	NO	NO	YES	NO	NO	NO	NO	NO		NO	YES	YES
VAGINAL BIRTH	NO	NO	FEMALE	8	9	2960	NO	NO	NO	NO	YES	NO	NO	NO	NO	NO		NO	NO	YES
VAGINAL BIRTH	NO	NO	FEMALE	8	9	3160	NO	NO	NO	NO	YES	NO	NO	NO	NO	NO		NO	NO	YES
VAGINAL BIRTH	YES	YES	FEMALE	8	9	3305	NO	NO	NO	NO	YES	NO	NO	NO	NO	NO		NO	YES	YES
VAGINAL BIRTH	NO	NO	FEMALE	9	9	3525	NO	NO	NO	NO	YES	NO	NO	NO	NO	NO		NO	NO	YES
VAGINAL BIRTH	NO	NO	FEMALE	8	9	2910	NO	NO	NO	NO	NO	NO	NO	NO	NO	NO		NO	NO	YES

VAGINAL BIRTH	NO	NO	MALE	8	8	3105	NO	NO	NO	NO	YES	NO	NO	NO	NO	NO		NO	NO	YES
VAGINAL BIRTH	NO	NO	FEMALE	9	9	2890	NO	NO	NO	NO	YES	NO	NO	NO	NO	NO		NO	NO	YES
C-SECTIONNO	NO	MALE	9	9	3205	NO	NO	NO	NO	NO	NO	NO	NO	NO	NO		NO	YES	YES	
C-SECTIONNO	NO	FEMALE	5	9	3135	NO	NO	NO	NO	NO	NO	NO	NO	NO	YES		NO	NO	YES	
C-SECTIONYES	NO	MALE	8	8	2185	YES	NO	NO	YES	YES	NO	NO	NO	NO	NO		NO	NO	YES	
VAGINAL BIRTH	YES	NO	MALE	0	0	520		NO	NO	NO	YES	NO	NO	YES	NO	NO		NO	NO	YES
VAGINAL BIRTH	YES	NO	MALE	8	9	2044	YES	NO	NO	NO	NO	NO	NO	YES	NO	NO		NO	NO	YES
VAGINAL BIRTH	NO	NO	MALE	9	10	2380	NO	NO	NO	NO	NO	NO	NO	NO	NO	NO		NO	YES	YES
C-SECTIONYES	NO	MALE	8	9	1890	YES	NO	NO	YES	YES	NO	NO	NO	NO	NO		NO	NO	YES	
C-SECTIONYES	NO	MALE	3	7	1540	YES	YES	YES	NO	YES	NO	NO	NO	NO	YES		NO	NO	YES	
VAGINAL BIRTH	YES	NO	MALE	9	10	2250	NO	NO	NO	NO	NO	NO	NO	NO	NO	NO		NO	NO	YES
VAGINAL BIRTH	NO	NO	FEMALE	9	10	2315	NO	NO	NO	NO	NO	NO	NO	NO	NO	NO		NO	NO	YES
VAGINAL BIRTH	YES	NO	FEMALE	9	9	2410	NO	NO	YES	NO	YES	NO	NO	NO	NO	NO		NO	NO	YES
VAGINAL BIRTH	NO	NO	MALE	7	9	2440	NO	NO	NO	NO	YES	NO	NO	NO	NO	NO		NO	NO	YES
VAGINAL BIRTH	YES	NO	MALE	8	4	1365	YES	NO	NO	NO	YES	NO	NO	NO	NO	NO		NO	NO	YES
VAGINAL BIRTH	YES	NO	FEMALE	8	3	2500	YES	NO	YES	NO	YES	NO	NO	NO	NO	NO		NO	NO	YES
VAGINAL BIRTH	NO	NO	FEMALE	8	9	3475	NO	NO	NO	NO	NO	NO	NO	NO	NO	NO		NO	NO	YES
VAGINAL BIRTH	NO	NO	MALE	9	9	3185	NO	NO	NO	NO	NO	NO	NO	NO	NO	NO		NO	NO	YES
VAGINAL BIRTH	NO	NO	FEMALE	7	8	3125	NO	NO	NO	NO	NO	NO	NO	NO	NO	NO		NO	NO	YES
VAGINAL BIRTH	NO	NO	MALE	8	9	3490	NO	NO	NO	NO	YES	NO	NO	NO	NO	NO		NO	NO	YES
VAGINAL BIRTH	NO	NO	MALE	9	10	3085	NO	NO	NO	NO	YES	NO	NO	NO	NO	NO		NO	NO	YES
VAGINAL BIRTH	NO	NO	MALE	8	9	3700	NO	NO	NO	NO	YES	NO	NO	NO	NO	NO		NO	NO	YES
VAGINAL BIRTH	NO	NO	FEMALE	9	10	3460	NO	NO	NO	NO	NO	NO	NO	NO	NO	NO		NO	NO	YES
VAGINAL BIRTH	NO	NO	FEMALE	9	9	3025	NO	NO	NO	NO	NO	NO	NO	NO	NO	NO		NO	NO	YES
VAGINAL BIRTH	NO	NO	FEMALE	9	10	3025	NO	NO	NO	NO	YES	NO	NO	NO	NO	NO		NO	NO	YES
VAGINAL BIRTH	YES	NO	FEMALE	9	9	3160	NO	NO	NO	NO	NO	NO	NO	NO	YES	NO		NO	NO	YES
VAGINAL BIRTH	YES	NO	MALE	9	9	3040	NO	NO	NO	NO	NO	NO	NO	NO	NO	NO		NO	NO	YES
VAGINAL BIRTH	YES	NO	MALE	9	10	2835	NO	NO	NO	NO	YES	NO	NO	NO	YES	NO		NO	NO	YES
VAGINAL BIRTH	YES	NO	FEMALE	8	9	3345	NO	YES	NO	NO	NO	NO	NO	NO	NO	NO		NO	NO	YES
VAGINAL BIRTH	YES	NO	FEMALE	9	9	3015	NO	NO	NO	NO	NO	NO	NO	NO	YES	NO		NO	YES	YES
VAGINAL BIRTH	NO	NO	MALE	9	10	3140	NO	NO	NO	NO	NO	NO	NO	NO	NO	NO		NO	NO	YES
VAGINAL BIRTH	YES	NO	FEMALE	9	10	2670	NO	NO	NO	YES	YES	NO	NO	NO	NO	NO		NO	NO	YES
VAGINAL BIRTH	YES	NO	MALE	8	9	3270	NO	NO	NO	YES	YES	NO	NO	NO	NO	NO		NO	NO	YES
VAGINAL BIRTH	YES	NO	FEMALE	8	9	2850	NO	NO	NO	NO	NO	NO	NO	NO	NO	NO		NO	NO	YES
VAGINAL BIRTH	NO	NO	MALE	9	10	2930	NO	NO	NO	NO	NO	NO	NO	NO	NO	NO		NO	NO	YES
VAGINAL BIRTH	NO	YES	MALE	9	10	2835	YES	NO	NO	NO	NO	NO	NO	NO	NO	NO		NO	YES	YES
VAGINAL BIRTH	NO	NO	FEMALE	9	10	3310	NO	NO	NO	NO	NO	NO	NO	NO	NO	NO		NO	NO	YES
VAGINAL BIRTH	YES	NO	FEMALE	8	10	3640	NO	YES	NO	NO	NO	NO	NO	NO	NO	NO		NO	NO	YES
VAGINAL BIRTH	NO	NO	MALE	9	10	3415	NO	NO	NO	NO	YES	NO	NO	NO	NO	NO		NO	NO	YES
VAGINAL BIRTH	NO	NO	FEMALE	9	10	3300	NO	NO	NO	NO	NO	NO	NO	NO	NO	NO		NO	NO	YES
VAGINAL BIRTH	NO	NO	FEMALE	9	9	3405	NO	NO	NO	NO	YES	NO	NO	NO	NO	NO		NO	YES	YES
VAGINAL BIRTH	NO	NO	MALE	9	9	2630	NO	NO	NO	NO	NO	NO	NO	NO	NO	NO		NO	NO	YES
VAGINAL BIRTH	NO	NO	FEMALE	5	7	3510	NO	NO	NO	NO	YES	NO	NO	NO	NO	YES		NO	NO	YES
VAGINAL BIRTH	NO	NO	FEMALE	8	9	3700	NO	NO	NO	NO	NO	NO	NO	NO	NO	NO		NO	NO	YES
VAGINAL BIRTH	NO	NO	FEMALE	9	10	3065	NO	NO	NO	NO	NO	NO	NO	NO	NO	NO		NO	NO	YES
VAGINAL BIRTH	YES	NO	MALE	9	10	2775	NO	YES	NO	NO	NO	NO	NO	NO	NO	NO		NO	NO	YES
VAGINAL BIRTH	YES	NO	FEMALE	9	10	2785	NO	NO	NO	NO	YES	NO	NO	NO	NO	NO		NO	NO	YES
VAGINAL BIRTH	NO	NO	FEMALE	9	10	3330	NO	NO	NO	NO	YES	NO	NO	NO	NO	NO		NO	NO	YES
C-SECTIONNO	NO	FEMALE	9	10	3160	NO	NO	NO	NO	NO	NO	NO	NO	NO	NO		NO	NO	YES	
VAGINAL BIRTH	NO	NO	MALE	7	9	3555	NO	NO	NO	NO	YES	NO	NO	NO	NO	NO		NO	NO	YES
VAGINAL BIRTH	NO	NO	FEMALE	9	9	3015	NO	NO	NO	NO	NO	NO	NO	NO	NO	NO		NO	NO	YES
VAGINAL BIRTH	NO	NO	FEMALE	5	7	3190	YES	NO	NO	NO	YES	NO	NO	NO	NO	YES		NO	YES	YES
VAGINAL BIRTH	NO	NO	MALE	8	10	2860	NO	NO	NO	NO	NO	NO	NO	NO	NO	NO		NO	NO	YES

VAGINAL BIRTH	YES	NO	FEMALE	4	8	2785	YES	NO	NO	NO	YES	NO	NO	NO	NO	YES		NO	NO	YES
VAGINAL BIRTH	NO	NO	FEMALE	9	10	2545	NO	NO	NO	NO	YES	NO	NO	NO	NO	NO		NO	NO	YES
VAGINAL BIRTH	NO	NO	MALE	8	9	3250	NO	NO	NO	NO	NO	NO	NO	NO	NO	NO		NO	NO	YES
VAGINAL BIRTH	NO	NO	FEMALE	9	10	3380	NO	NO	NO	NO	YES	NO	NO	NO	NO	NO		NO	NO	YES
VAGINAL BIRTH	NO	NO	FEMALE	2	8	2720	YES	NO	NO	NO	NO	NO	NO	NO	NO	YES		NO	NO	YES
VAGINAL BIRTH	NO	NO	MALE	9	10	3025	NO	NO	NO	NO	NO	NO	NO	NO	NO	NO		NO	NO	YES
VAGINAL BIRTH	NO	NO	FEMALE	10	10	3780	NO	NO	NO	NO	YES	NO	NO	NO	NO	NO		NO	NO	YES
VAGINAL BIRTH	NO	NO	FEMALE	8	9	3375	NO	NO	NO	NO	YES	NO	NO	NO	NO	NO		NO	NO	YES
VAGINAL BIRTH	NO	NO	FEMALE	8	9	3410	NO	NO	NO	NO	NO	NO	NO	NO	NO	NO		NO	NO	YES
VAGINAL BIRTH	NO	NO	MALE	9	10	3230	NO	NO	NO	NO	NO	NO	NO	NO	NO	NO		NO	NO	YES
VAGINAL BIRTH	NO	NO	MALE	8	9	3385	NO	NO	NO	NO	NO	NO	NO	NO	NO	NO		NO	NO	YES
C-SECTIONYES	NO	MALE	9	9	3170	NO	NO	NO	NO	NO	NO	NO	NO	YES	NO		NO	YES	YES	
VAGINAL BIRTH	NO	NO	FEMALE	9	9	2860	NO	NO	NO	NO	YES	NO	NO	NO	NO	NO		NO	NO	YES
VAGINAL BIRTH	NO	NO	MALE	9	9	3590	NO	NO	NO	NO	YES	NO	NO	NO	NO	NO		NO	NO	YES
VAGINAL BIRTH	NO	NO	FEMALE	9	10	3235	NO	NO	NO	NO	YES	NO	NO	NO	NO	NO		NO	NO	YES
VAGINAL BIRTH	YES	NO	MALE	8	9	2675	NO	NO	NO	NO	YES	NO	NO	NO	NO	NO		NO	YES	YES
C-SECTIONYES	NO	FEMALE	8	9	2860	NO	NO	NO	NO	YES	NO	NO	NO	NO	NO		NO	NO	YES	
VAGINAL BIRTH	NO	NO	MALE	9	9	2715	NO	NO	NO	NO	NO	NO	NO	NO	NO	NO		NO	NO	YES
VAGINAL BIRTH	YES	NO	FEMALE	9	9	2820	NO	NO	NO	NO	YES	NO	NO	NO	NO	NO		NO	NO	YES
VAGINAL BIRTH	NO	NO	MALE	9	9	3445	NO	NO	NO	NO	YES	NO	NO	NO	NO	NO		NO	NO	YES
VAGINAL BIRTH	YES	NO	FEMALE	5	9	3015	NO	NO	YES	NO	YES	NO	NO	NO	NO	NO		NO	NO	YES
VAGINAL BIRTH	NO	NO	MALE	9	10	3785	NO	NO	NO	NO	NO	NO	NO	NO	NO	NO		NO	NO	YES
VAGINAL BIRTH	NO	NO	MALE	9	10	3550	NO	NO	NO	NO	NO	NO	NO	NO	NO	NO		NO	NO	YES
VAGINAL BIRTH	NO	NO	FEMALE	9	9	2965	NO	NO	NO	NO	NO	NO	NO	NO	NO	NO		NO	NO	YES
VAGINAL BIRTH	NO	NO	FEMALE	9	8	2900	NO	NO	NO	NO	YES	NO	NO	NO	NO	NO		NO	NO	YES
VAGINAL BIRTH	NO	NO	FEMALE	8	9	3460	NO	NO	NO	NO	YES	NO	NO	NO	NO	NO		NO	NO	YES
VAGINAL BIRTH	NO	NO	FEMALE	9	10	3370	NO	NO	NO	NO	YES	NO	NO	NO	NO	NO		NO	NO	YES
C-SECTIONNO	NO	MALE	8	9	3770	NO	NO	NO	NO	YES	NO	NO	NO	NO	NO		NO	NO	YES	
VAGINAL BIRTH	YES	NO	MALE	9	10	3435	NO	NO	NO	NO	YES	NO	NO	NO	YES	NO		NO	NO	YES
VAGINAL BIRTH	NO	NO	MALE	7	9	3460	NO	NO	NO	NO	YES	NO	NO	NO	NO	NO		NO	NO	YES
VAGINAL BIRTH	NO	NO	MALE	8	8	3340	YES	NO	NO	NO	YES	NO	NO	NO	NO	NO		NO	YES	YES
VAGINAL BIRTH	NO	NO	FEMALE	8	9	3550	NO	NO	NO	NO	YES	NO	NO	NO	NO	NO		NO	NO	YES
VAGINAL BIRTH	NO	NO	MALE	8	9	3930	NO	NO	NO	NO	NO	NO	NO	NO	NO	NO		NO	NO	YES
VAGINAL BIRTH	NO	NO	MALE	9	10	3020	NO	NO	NO	NO	NO	NO	NO	NO	NO	NO		NO	NO	YES
VAGINAL BIRTH	NO	NO	FEMALE	9	10	2680	NO	NO	NO	NO	YES	NO	NO	NO	NO	NO		NO	NO	YES
VAGINAL BIRTH	NO	NO	FEMALE	9	9	3095	NO	NO	NO	NO	NO	NO	NO	NO	NO	NO		NO	NO	YES
VAGINAL BIRTH	YES	NO	MALE	6	9	1390	YES	NO	NO	NO	NO	YES	NO	NO	YES	YES		NO	NO	YES
VAGINAL BIRTH	YES	NO	FEMALE	5	8	1250	YES	NO	NO	NO	YES	NO	NO	NO	NO	YES		NO	NO	YES
VAGINAL BIRTH	NO	NO	MALE	9	9	3075	NO	NO	NO	NO	YES	NO	NO	NO	NO	NO		NO	NO	YES
VAGINAL BIRTH	YES	NO	MALE	9	9	2760	NO	NO	NO	NO	YES	NO	NO	NO	NO	NO		NO	NO	YES
VAGINAL BIRTH	NO	NO	MALE	9	10	2985	NO	NO	NO	NO	YES	NO	NO	NO	NO	NO		NO	NO	YES
C-SECTIONYES	NO	MALE	8	10	2995	NO	NO	NO	NO	YES	NO	NO	NO	NO	NO		NO	NO	YES	
VAGINAL BIRTH	NO	NO	FEMALE	9	10	3210	NO	NO	NO	NO	YES	NO	NO	NO	NO	NO		NO	NO	YES
VAGINAL BIRTH	NO	NO	MALE	8	9	2905	NO	NO	NO	NO	YES	NO	NO	NO	NO	NO		NO	NO	YES
VAGINAL BIRTH	NO	NO	FEMALE	9	10	3265	NO	NO	NO	NO	YES	NO	NO	NO	NO	NO		NO	NO	YES
C-SECTIONNO	NO	MALE	9	10	2790	NO	NO	NO	NO	YES	NO	NO	NO	NO	NO		NO	NO	YES	
VAGINAL BIRTH	NO	YES	MALE	9	9	3060	NO	NO	NO	NO	YES	NO	NO	NO	NO	NO		NO	YES	YES
VAGINAL BIRTH	YES	NO	MALE	9	9	2735	NO	NO	NO	NO	NO	NO	NO	NO	YES	NO		NO	NO	YES
VAGINAL BIRTH	YES	NO	FEMALE	8	9	2775	NO	NO	NO	NO	NO	NO	NO	NO	NO	NO		NO	NO	YES
VAGINAL BIRTH	NO	NO	MALE	8	9	3495	NO	NO	NO	NO	NO	NO	NO	NO	NO	NO		NO	NO	YES
VAGINAL BIRTH	YES	NO	MALE	10	10	3505	NO	YES	NO	NO	YES	NO	NO	NO	NO	NO		NO	NO	YES
VAGINAL BIRTH	NO	NO	FEMALE	8	9	3220	NO	NO	NO	NO	NO	NO	NO	NO	NO	NO		NO	YES	YES
VAGINAL BIRTH	NO	NO	FEMALE	9	9	3110	NO	NO	NO	NO	NO	NO	NO	NO	NO	NO		NO	YES	YES

VAGINAL BIRTH		YES	NO	MALE	9	9	3915	NO	NO	NO	NO	YES	NO	NO	NO	NO	NO		NO	YES	YES
VAGINAL BIRTH		NO	NO	MALE	8	9	3335	NO	NO	NO	NO	YES	NO	NO	NO	NO	NO		NO	NO	YES
VAGINAL BIRTH		YES	NO	FEMALE	9	9	2590	NO	NO	NO	NO	NO	NO	NO	NO	YES	NO		NO	NO	YES
VAGINAL BIRTH		NO	NO	MALE	8	9	3045	NO	NO	NO	NO	YES	NO	NO	NO	NO	NO		NO	NO	YES
C-SECTION	NO	NO	FEMALE	8	9	3610	NO	NO	NO	NO	YES	NO	NO	NO	NO	NO		NO	NO	YES	
VAGINAL BIRTH		NO	NO	FEMALE	9	9	3005	NO	NO	NO	NO	YES	NO	NO	NO	NO	NO		NO	NO	YES
VAGINAL BIRTH		NO	NO	MALE	9	9	3230	NO	NO	NO	NO	NO	NO	NO	NO	NO	NO		NO	NO	YES
C-SECTION	YES	NO	MALE	6	8	2285	YES	NO	NO	YES	YES	NO	NO	NO	NO	YES		NO	NO	YES	
C-SECTION	YES	NO	FEMALE	8	9	1935	YES	NO	NO	YES	YES	NO	NO	NO	NO	NO		NO	NO	YES	
VAGINAL BIRTH		YES	NO	MALE	9	9	3220	NO	NO	NO	NO	YES	NO	NO	NO	NO	NO		NO	NO	YES
VAGINAL BIRTH		YES	NO	MALE	9	9	2125	NO	YES	NO	NO	YES	NO	NO	NO	NO	NO	20,00	NO	NO	NO
VAGINAL BIRTH		YES	NO	FEMALE	8	10	2840	NO	NO	NO	NO	YES	NO	NO	NO	YES	NO	20,00	NO	YES	NO
C-SECTION	YES	NO	MALE	1	8	2780	YES	NO	YES	NO	YES			NO	NO	YES	20,00	NO	NO	NO	
VAGINAL BIRTH		NO	NO	MALE	9	10	2860	NO	NO	NO	NO	YES	NO	NO	NO	NO	NO	20,00	NO	NO	YES
VAGINAL BIRTH		NO	NO	FEMALE	8	9	3070	NO	NO	NO	NO	YES	NO	NO	NO	NO	NO	20,14	NO	NO	YES
C-SECTION	NO	NO	MALE	8	9	4020	NO	NO	NO	NO	YES	NO	NO	NO	NO	NO	20,29	NO	NO	NO	
C-SECTION	NO	NO	MALE	8	9	2600	NO	NO	NO	NO	YES			NO	NO	NO	20,29	NO	NO	NO	
C-SECTION	YES	NO	FEMALE	8	8	2050	YES	YES	NO	NO	YES	NO	NO	NO	NO	NO	20,29	NO	NO	NO	
C-SECTION	YES	NO	MALE	8	9	3520	YES	NO	YES	NO	YES	NO	NO	NO	NO	NO	20,29	NO	NO	NO	
C-SECTION	NO	NO	MALE	10	10	4015	NO	NO	NO	NO	YES	NO	YES	NO	NO	NO	20,43	NO	YES	NO	
C-SECTION	YES	NO	MALE	8	9	3790	NO	NO	YES	NO	YES	NO	NO	NO	NO	NO	20,43	NO	NO	NO	
C-SECTION	YES	NO	MALE	9	9	2615	NO	YES	NO	NO	YES	NO	NO	NO	NO	NO	20,43	NO	NO	NO	
C-SECTION	NO	NO	MALE	7	9	3950	YES	NO	NO	NO	YES	NO	NO	NO	NO	NO	20,43	NO	NO	NO	
C-SECTION	YES	NO	FEMALE	9	9	1200	YES	YES	NO	YES	YES	NO	NO	NO	NO	NO	20,43	NO	NO	NO	
C-SECTION	YES	NO	MALE	7	10	1310	YES	YES	NO	NO	YES	NO	NO	NO	NO	NO	20,43	NO	NO	NO	
C-SECTION	YES	NO	MALE	9	9	2835	NO	NO	NO	NO	YES	NO	NO	NO	NO	NO	20,43	NO	NO	NO	
VAGINAL BIRTH		YES	NO	MALE	9	10	2610	NO	NO	YES	NO	YES	NO	NO	NO	NO	NO	20,43	NO	NO	YES
VAGINAL BIRTH		NO	NO	FEMALE	8	9	2975	NO	NO	NO	NO	YES	NO	NO	NO	NO	NO	20,43	NO	NO	YES
C-SECTION	YES	NO	MALE	8	9	2875	NO	NO	YES	NO	YES	NO	NO	NO	NO	NO	20,43	NO	NO	YES	
C-SECTION	YES	NO	MALE	8	9	3030	NO	NO	NO	NO	YES	NO	NO	NO	NO	NO	20,43	NO	NO	YES	
VAGINAL BIRTH		NO	NO	MALE	9	9	3000	NO	NO	NO	NO	YES	NO	NO	NO	NO	NO	20,57	NO	NO	YES
VAGINAL BIRTH		YES	NO	MALE	7	8	3330	NO	YES	NO	NO	YES	NO	NO	NO	NO	NO	20,57	NO	YES	YES
VAGINAL BIRTH		YES	NO	FEMALE	7	8	2780	NO	NO	YES	NO	YES	NO	NO	NO	NO	NO	20,57	NO	NO	YES
VAGINAL BIRTH		NO	NO	MALE	8	8	2650	NO	NO	NO	NO	YES	NO	NO	NO	NO	NO	20,71	NO	NO	NO
C-SECTION	NO	NO	MALE	7	7	3625	NO	NO	NO	NO	YES	NO	NO	NO	NO	NO	20,71	NO	NO	NO	
C-SECTION	YES	NO	MALE	8	7	4260	YES	YES	NO	NO	YES	NO	NO	NO	NO	NO	20,71	NO	NO	NO	
VAGINAL BIRTH		YES	NO	MALE	9	10	2030	YES	NO	NO	NO	YES	NO	NO	NO	NO	NO	20,71	NO	NO	YES
VAGINAL BIRTH		NO	NO	MALE	9	10	3855	NO	NO	NO	NO	YES	NO	NO	NO	NO	NO	20,71	NO	NO	YES
VAGINAL BIRTH		NO	NO	FEMALE	9	10	3310	NO	NO	NO	NO	YES	NO	NO	NO	NO	NO	20,71	NO	NO	YES
VAGINAL BIRTH		YES	NO	MALE	8	9	2950	NO	NO	NO	NO	YES	NO	NO	NO	NO	NO	20,71	NO	NO	YES
VAGINAL BIRTH		NO	NO	MALE	10	10	3625	NO	NO	NO	NO	YES	NO	NO	NO	NO	NO	20,71	NO	NO	YES
VAGINAL BIRTH		NO	NO	MALE	8	9	3835	YES	NO	NO	NO	YES	NO	NO	NO	NO	NO	20,86	NO	YES	NO
C-SECTION	YES	NO	MALE	8	9	2645	NO	NO	NO	NO	YES	NO	NO	NO	NO	NO	20,86	NO	NO	NO	
C-SECTION	NO	NO	MALE	8	9	3875	NO	NO	NO	NO	YES	NO	NO	NO	NO	NO	20,86	NO	NO	NO	
VAGINAL BIRTH		YES	NO	FEMALE	9	9	2645	YES	NO	NO	NO	YES	NO	NO	NO	NO	NO	20,86	NO	NO	YES
VAGINAL BIRTH		YES	NO	MALE	0	0	1450		NO	NO	NO	YES	NO	NO	YES	NO	NO	21,00	NO	NO	YES
C-SECTION	YES	NO	MALE	9	10	2860	NO	NO	NO	NO	YES	NO	NO	NO	NO	NO	21,00	NO	NO	YES	
C-SECTION	YES	NO	MALE	9	9	4350	NO	YES	NO	NO	YES	NO	NO	NO	NO	NO	21,14	NO	NO	NO	
VAGINAL BIRTH		NO	NO	MALE	9	10	3370	NO	NO	NO	NO	YES	NO	NO	NO	NO	NO	21,14	NO	NO	YES
C-SECTION	YES	NO	MALE	8	9	2970	NO	YES	YES	NO	YES	NO	NO	NO	NO	NO	21,29	NO	NO	NO	
VAGINAL BIRTH		NO	NO	FEMALE	9	9	2450	NO	NO	NO	NO	YES	NO	NO	NO	NO	NO	21,29	NO	YES	YES
VAGINAL BIRTH		NO	NO	FEMALE	9	10	3210	NO	NO	NO	NO	YES	NO	NO	NO	NO	NO	21,29	NO	NO	YES
VAGINAL BIRTH		NO	NO	MALE	9	9	3455	NO	NO	NO	NO	YES	NO	NO	NO	NO	NO	21,29	NO	NO	YES

VAGINAL BIRTH	NO	YES	NO	UNDETERMINATED		0	0	350		NO	NO	NO	YES	NO	NO	YES	NO	NO	21,43	NO	NO
VAGINAL BIRTH	NO	YES	NO	UNDETERMINATED		0	0	350		NO	NO	NO	YES	NO	NO	YES	NO	NO	21,43	NO	NO
C-SECTION	NO	NO	MALE	8	9	3280	NO	NO	NO	NO	YES	NO	NO	NO	NO	NO	21,43	NO	NO	NO	
VAGINAL BIRTH		YES	NO	MALE	8	8	3285	YES	NO	NO	NO	YES	NO	NO	NO	NO	NO	21,43	NO	YES	YES
VAGINAL BIRTH		YES	NO	FEMALE	8	9	2560	NO	NO	NO	NO	YES	NO	NO	NO	NO	NO	21,43	NO	NO	YES
C-SECTION	NO	NO	FEMALE	8	9	3785	NO	NO	NO	NO	YES	NO	NO	NO	NO	NO	21,43	NO	NO	YES	
VAGINAL BIRTH		YES	NO	FEMALE	9	10	3320	NO	NO	NO	NO	YES	NO	NO	NO	NO	NO	21,43	NO	NO	YES
VAGINAL BIRTH		NO	NO	FEMALE	9	10	3420	NO	NO	NO	NO	YES	NO	NO	NO	NO	NO	21,43	NO	YES	YES
C-SECTION	NO	NO	MALE	8	9	2930	NO	NO	NO	NO	YES	NO	NO	NO	NO	NO	21,43	NO	NO	YES	
VAGINAL BIRTH		YES	NO	MALE	9	10	3120	NO	NO	NO	NO	YES	NO	NO	NO	YES	NO	21,43	NO	YES	YES
VAGINAL BIRTH		YES	NO	FEMALE	9	10	3355	NO	NO	YES	NO	YES	NO	NO	NO	NO	NO	21,57	NO	NO	NO
VAGINAL BIRTH		NO	NO	MALE	8	9	2750	NO	NO	NO	NO	YES	NO	NO	NO	NO	NO	21,57	NO	YES	YES
VAGINAL BIRTH		NO	NO	MALE	9	9	2745	NO	NO	NO	NO	YES	NO	NO	NO	NO	NO	21,57	NO	NO	YES
VAGINAL BIRTH		NO	NO	FEMALE	8	9	3690	NO	NO	NO	NO	YES	NO	NO	NO	NO	NO	21,71	NO	YES	NO
VAGINAL BIRTH		YES	NO	MALE			400		NO	NO	NO	YES	NO	NO	YES	NO	NO	21,71	NO	NO	NO
VAGINAL BIRTH		NO	NO	MALE	4	8	3550	YES	NO	NO	NO	YES	NO	NO	NO	NO	YES	21,71	NO	YES	YES
VAGINAL BIRTH		NO	NO	FEMALE	8	9	3685	NO	NO	NO	NO	YES	NO	NO	NO	NO	NO	21,86	NO	NO	NO
VAGINAL BIRTH		NO	YES	MALE	9	9	3600	NO	NO	NO	NO	YES	NO	NO	NO	NO	NO	21,86	NO	NO	NO
C-SECTION	YES	NO	MALE	9	10	3575	NO	NO	YES	NO	YES	NO	NO	NO	NO	NO	21,86	NO	NO	NO	
VAGINAL BIRTH		NO	NO	MALE	9	10	2785	NO	NO	NO	NO	YES	NO	NO	NO	NO	NO	21,86	NO	NO	YES
VAGINAL BIRTH		NO	NO	MALE	9	9	3300	NO	NO	NO	NO	YES	NO	NO	NO	NO	NO	21,86	NO	NO	YES
C-SECTION	YES	NO	FEMALE	8	10	2830	NO	YES	NO	NO	YES	NO	NO	NO	NO	NO	22,00	NO	NO	NO	
VAGINAL BIRTH		NO	NO	FEMALE	8	9	3530	NO	NO	NO	NO	YES	NO	NO	NO	NO	NO	22,29	NO	YES	NO
C-SECTION	NO	NO	FEMALE	8	9	2930	NO	NO	NO	NO	YES	NO	NO	NO	NO	NO	22,29	NO	NO	NO	
VAGINAL BIRTH		YES	NO	MALE	9	10	2775	NO	NO	NO	NO	YES	NO	NO	NO	YES	NO	22,29	NO	NO	YES
VAGINAL BIRTH		YES	NO	MALE	0	0	575	NO	NO	NO	NO	YES	NO	NO	YES	NO	NO	22,43	NO	NO	NO
C-SECTION	YES	NO	FEMALE	9	10	3300	NO	NO	NO	NO	YES	NO	NO	NO	YES	NO	22,43	NO	NO	NO	
VAGINAL BIRTH		NO	NO	FEMALE	9	10	2875	NO	NO	NO	NO	YES	NO	NO	NO	NO	NO	22,43	NO	NO	YES
VAGINAL BIRTH		YES	YES	FEMALE			2250		YES	NO	NO	YES	NO	YES	YES	NO	NO	22,43	NO	NO	YES
C-SECTION	YES	NO	FEMALE	8	9	2755	NO	NO	NO	NO	YES	NO	NO	NO	NO	NO	22,57	NO	NO	YES	
VAGINAL BIRTH		NO	NO	FEMALE	9	9	2950	NO	NO	NO	NO	YES	NO	NO	NO	NO	NO	22,71	NO	YES	NO
VAGINAL BIRTH		NO	NO	MALE	8	9	3625	NO	NO	NO	NO	YES	NO	NO	NO	NO	NO	22,71	NO	NO	YES
C-SECTION	NO	NO	FEMALE	8	9	3950	NO	NO	NO	NO	YES	NO	NO	NO	NO	NO	22,71	NO	NO	YES	
C-SECTION	NO	NO	FEMALE	8	9	3110	NO	NO	NO	NO	YES	NO	NO	NO	NO	NO	22,86	NO	NO	NO	
C-SECTION	YES	NO	FEMALE	9	10	3430	NO	NO	YES	NO	YES	NO	NO	NO	NO	NO	22,86	NO	NO	NO	
C-SECTION	YES	NO	MALE	8	8	1675	YES	NO	NO	NO	YES	NO	NO	NO	NO	NO	23,00	NO	NO	NO	
VAGINAL BIRTH		YES	NO	MALE	8	9	3313	NO	YES	NO	NO	YES	NO	NO	NO	NO	NO	23,00	NO	NO	YES
C-SECTION	YES	NO	MALE	9	10	5110	NO	YES	YES	NO	YES	NO	NO	NO	NO	NO	23,14	NO	NO	NO	
VAGINAL BIRTH		YES	YES	MALE	8	10	3200	NO	NO	NO	NO	YES	NO	NO	NO	YES	NO	23,14	NO	YES	YES
VAGINAL BIRTH		YES	NO	FEMALE	9	9	3360	NO	NO	NO	NO	YES	NO	NO	NO	NO	NO	23,29	NO	NO	NO
VAGINAL BIRTH		NO	NO	MALE	9	9	3490	NO	NO	NO	NO	YES	NO	NO	NO	NO	NO	23,29	NO	NO	YES
VAGINAL BIRTH		NO	NO	MALE	9	10	3005	NO	NO	NO	NO	YES	NO	NO	NO	NO	NO	23,29	NO	NO	YES
VAGINAL BIRTH		YES	NO	MALE	9	9	3010	NO	NO	NO	NO	YES	NO	NO	NO	YES	NO	23,29	NO	NO	YES
VAGINAL BIRTH		YES	NO	MALE	0	0	2385		NO	NO	NO	YES	NO	NO	NO	NO	NO	23,43	NO	NO	NO
VAGINAL BIRTH		NO	NO	FEMALE	9	9	3810	NO	NO	NO	NO	YES	NO	NO	NO	NO	NO	23,43	NO	YES	NO
VAGINAL BIRTH		NO	NO	MALE	7	10	3605	NO	NO	NO	NO	YES	NO	NO	NO	NO	NO	23,43	NO	YES	NO
VAGINAL BIRTH		NO	NO	MALE	9	10	3770	NO	NO	NO	NO	YES	NO	NO	NO	NO	NO	23,43	NO	NO	YES
VAGINAL BIRTH		NO	NO	MALE	9	9	3145	NO	NO	NO	NO	YES	NO	NO	NO	NO	NO	23,43	NO	NO	YES
VAGINAL BIRTH		NO	NO	MALE	9	10	3860	NO	NO	NO	NO	YES	NO	NO	NO	NO	NO	23,43	NO	NO	YES
VAGINAL BIRTH		NO	NO	FEMALE	9	9	3260	NO	NO	NO	NO	YES			NO	NO	NO	23,43	NO	NO	YES
C-SECTION	NO	NO	MALE	8	9	2650	NO	NO	NO	NO	YES	NO	NO	NO	NO	NO	23,43	NO	NO	YES	

VAGINAL BIRTH	NO	NO	MALE	9	10	3200	NO	NO	NO	NO	YES	NO	NO	NO	NO	NO	23,57	NO	YES	YES
VAGINAL BIRTH	YES	NO	FEMALE	0	0	755		NO	NO	NO	YES	NO	NO	YES	NO	NO	23,57	NO	NO	YES
VAGINAL BIRTH	YES	NO	MALE	9	9	2600	NO	NO	NO	NO	YES	NO	NO	NO	NO	NO	23,71	NO	NO	YES
VAGINAL BIRTH	YES	NO	MALE	1	0	2565		NO	NO	NO	YES	NO	NO	YES	NO	YES	23,86	NO	NO	NO
VAGINAL BIRTH	NO	NO	MALE	9	9	3495	NO	NO	NO	NO	YES	NO	NO	NO	NO	NO	23,86	NO	NO	YES
VAGINAL BIRTH	YES	NO	MALE	9	9	3105	NO	NO	NO	NO	YES	NO	NO	NO	NO	NO	23,86	NO	NO	YES
C-SECTIONYES	NO	FEMALE	8	9	2505	NO	NO	NO	NO	YES	NO	NO	NO	NO	NO	23,86	NO	NO	YES	
VAGINAL BIRTH	NO	NO	MALE	9	10	2730	NO	NO	NO	NO	YES	NO	NO	NO	NO	NO	24,29	NO	NO	YES
VAGINAL BIRTH	NO	NO	MALE	9	9	2805	NO	NO	NO	NO	YES	NO	NO	NO	NO	NO	24,29	NO	NO	YES
C-SECTIONYES	NO	FEMALE	8	9	2420	YES	NO	NO	NO	YES	YES	NO	NO	NO	NO	24,43	NO	NO	NO	
VAGINAL BIRTH	YES	NO	MALE	8	9	2280	NO	YES	NO	NO	YES	NO	NO	NO	NO	NO	24,43	NO	NO	NO
VAGINAL BIRTH	NO	NO	FEMALE	9	10	2390	NO	NO	NO	NO	YES	NO	NO	NO	NO	NO	24,43	NO	NO	NO
VAGINAL BIRTH	NO	NO	MALE	9	9	2820	NO	NO	NO	NO	YES	NO	NO	NO	NO	NO	24,43	NO	NO	YES
VAGINAL BIRTH	NO	NO	MALE	8	9	3630	NO	NO	NO	NO	YES	NO	NO	NO	NO	NO	24,57	NO	NO	YES
VAGINAL BIRTH	YES	NO	MALE	9	10	2353	NO	NO	NO	NO	YES	NO	NO	NO	YES	NO	24,71	NO	NO	YES
C-SECTIONYES	NO	MALE	9	10	3360	NO	NO	YES	NO	YES	NO	NO	NO	NO	NO	25,00	NO	NO	NO	
VAGINAL BIRTH	NO	YES	MALE	9	9	3010	NO	NO	NO	NO	YES	NO	NO	NO	NO	NO	25,00	NO	NO	YES
VAGINAL BIRTH	NO	NO	FEMALE	9	10	3250	NO	NO	NO	NO	YES	NO	NO	NO	NO	NO	25,00	NO	NO	YES
VAGINAL BIRTH	NO	NO	MALE	9	10	3800	YES	NO	NO	NO	YES	NO	NO	NO	NO	NO	25,00	NO	NO	YES
C-SECTIONYES	NO	FEMALE	2	8	1060	YES	NO	NO	NO	YES	NO	NO	NO	NO	YES	25,00	NO	NO	YES	
C-SECTIONYES	NO	FEMALE	7	8	1285	YES	NO	NO	NO	YES	NO	NO	YES	NO	NO	25,29	NO	NO	NO	
C-SECTIONYES	NO	FEMALE	2	7	1225	YES	NO	NO	NO	YES	NO	NO	NO	NO	YES	25,29	NO	NO	NO	
VAGINAL BIRTH	NO	NO	MALE	8	9	3640	NO	NO	NO	NO	YES	NO	NO	NO	NO	NO	25,29	NO	NO	YES
C-SECTIONYES	NO	MALE	8	9	3605	NO	YES	NO	NO	YES	NO	NO	NO	NO	NO	25,43	NO	NO	NO	
C-SECTIONYES	NO	MALE	8	9	3040	NO	NO	NO	NO	YES	NO	NO	NO	YES	NO	25,43	NO	NO	YES	
VAGINAL BIRTH	NO	NO	FEMALE	7	8	3340	NO	NO	NO	NO	YES	NO	NO	NO	NO	NO	25,43	NO	NO	YES
VAGINAL BIRTH	NO	NO	FEMALE	9	10	3100	NO	NO	NO	NO	YES	NO	NO	NO	NO	NO	25,71	NO	NO	NO
VAGINAL BIRTH	NO	NO	MALE	9	10	3050	NO	NO	NO	NO	YES	NO	NO	NO	NO	NO	25,71	NO	NO	YES
VAGINAL BIRTH	NO	NO	FEMALE	9	10	3070	NO	NO	NO	NO	YES	NO	NO	NO	NO	NO	25,71	NO	NO	YES
C-SECTIONYES	NO	MALE	9	10	2700	NO	NO	NO	NO	YES	NO	NO	NO	NO	NO	26,00	NO	NO	NO	
VAGINAL BIRTH	NO	NO	MALE	8	9	3360	NO	NO	NO	NO	YES	NO	NO	NO	NO	NO	26,00	NO	NO	YES
VAGINAL BIRTH	NO	NO	FEMALE	9	10	3400	NO	NO	NO	NO	YES	NO	NO	NO	NO	NO	26,14	NO	NO	YES
VAGINAL BIRTH	YES	NO	MALE	8	9	3275	NO	NO	NO	NO	YES	NO	NO	NO	NO	NO	26,29	NO	NO	YES
C-SECTIONYES	NO	MALE	8	9	2990	NO	NO	NO	NO	YES	NO	NO	NO	NO	NO	26,43	NO	NO	NO	
C-SECTIONYES	NO	FEMALE	9	9	3770	NO	NO	NO	NO	YES	NO	NO	NO	NO	NO	26,43	NO	NO	NO	
VAGINAL BIRTH	YES	NO	FEMALE	8	9	2245	YES	YES	NO	NO	YES	NO	NO	NO	NO	NO	26,43	NO	YES	YES
VAGINAL BIRTH	YES	NO	FEMALE	9	10	3075	NO	NO	NO	NO	YES	NO	NO	NO	NO	NO	26,43	NO	NO	YES
VAGINAL BIRTH	YES	NO	FEMALE	9	9	2190	NO	YES	NO	NO	YES	NO	NO	NO	NO	NO	26,57	NO	NO	NO
VAGINAL BIRTH	YES	NO	FEMALE	9	9	2740	NO	NO	NO	NO	YES	NO	NO	NO	NO	NO	26,71	NO	YES	YES
VAGINAL BIRTH	NO	NO	FEMALE	8	9	2640	NO	NO	NO	NO	YES	NO	NO	NO	NO	NO	26,71	NO	NO	YES
VAGINAL BIRTH	YES	NO	FEMALE	9	9	2680	NO	NO	NO	NO	YES	NO	NO	NO	YES	NO	26,71	NO	YES	YES
VAGINAL BIRTH	YES	NO	FEMALE	9	10	2710	NO	NO	NO	NO	YES	NO	NO	NO	NO	NO	26,86	NO	YES	YES
VAGINAL BIRTH	YES	NO	MALE	9	10	2610	NO	YES	NO	NO	YES	NO	NO	NO	NO	NO	26,86	NO	NO	YES
VAGINAL BIRTH	NO	NO	MALE	9	10	3355	NO	NO	NO	NO	YES	NO	NO	NO	NO	NO	27,00	NO	NO	YES
VAGINAL BIRTH	NO	YES	FEMALE	8	10	3445	NO	NO	NO	NO	YES			NO	NO	NO	27,00	NO	YES	YES
VAGINAL BIRTH	NO	NO	FEMALE	8	9	3540	NO	NO	NO	NO	YES	NO	NO	NO	NO	NO	27,43	NO	NO	YES
VAGINAL BIRTH	YES	NO	FEMALE	9	10	3020	NO	NO	NO	NO	YES	NO	NO	NO	NO	NO	28,43	NO	NO	YES
VAGINAL BIRTH	NO	NO	MALE	9	10	2830	NO	NO	NO	NO	YES	NO	NO	NO	NO	NO	28,43	NO	NO	YES
VAGINAL BIRTH	NO	NO	FEMALE	9	9	3150	NO	NO	NO	NO	YES	NO	NO	NO	NO	NO	28,43	NO	NO	YES
VAGINAL BIRTH	NO	NO	FEMALE	9	10	3405	NO	NO	NO	NO	YES	NO	NO	NO	NO	NO	28,43	NO	NO	YES
C-SECTIONNO	NO	FEMALE	9	10	3160	NO	NO	NO	NO	YES	NO	NO	NO	NO	NO	28,57	NO	NO	YES	
VAGINAL BIRTH	NO	NO	MALE	9	10	3225	NO	NO	NO	NO	YES	NO	NO	NO	NO	NO	28,86	NO	NO	YES
VAGINAL BIRTH	YES	NO	MALE	9	10	3610	NO	NO	NO	NO	YES	NO	NO	NO	NO	NO	28,86	NO	NO	YES

VAGINAL BIRTH		NO	NO	FEMALE	9	10	3070	NO	NO	NO	NO	YES			NO	NO	NO	29,00	NO	NO	YES
VAGINAL BIRTH		NO	NO	FEMALE	9	10	2630	NO	NO	NO	NO	YES	NO	NO	NO	NO	NO	29,29	NO	NO	YES
VAGINAL BIRTH		YES	NO	FEMALE	8	9	2505	NO	NO	NO	NO	YES	NO	NO	NO	NO	NO	29,43	NO	NO	YES
VAGINAL BIRTH		NO	NO	MALE	7	9	3295	NO	NO	NO	NO	YES	NO	NO	NO	NO	NO	29,43	NO	YES	YES
C-SECTION	NO	NO	FEMALE	9	9	3865	NO	NO	NO	NO	YES	NO	NO	NO	NO	NO	29,57	NO	NO	NO	
VAGINAL BIRTH		YES	NO	FEMALE	9	9	2630	NO	NO	NO	NO	YES	NO	NO	NO	NO	NO	29,86	NO	NO	YES
VAGINAL BIRTH		YES	NO	FEMALE	9	9	2395	NO	NO	NO	NO	YES	NO	NO	NO	NO	NO	30,00	NO	NO	NO
VAGINAL BIRTH		NO	NO	MALE	9	10	3495	NO	NO	NO	NO	YES	NO	NO	NO	NO	NO	30,00	NO	NO	YES
C-SECTION	YES	NO	FEMALE	9	9	2785	NO	YES	NO	NO	YES	NO	NO	NO	NO	NO	30,43	NO	NO	NO	
C-SECTION	NO	NO	MALE	9	10	3625	NO	NO	NO	NO	YES	NO	NO	NO	NO	NO	30,43	NO	NO	NO	
C-SECTION	YES	NO	FEMALE	7	8	2720	NO	NO	NO	NO	YES	NO	NO	NO	NO	NO	30,43	NO	NO	YES	
VAGINAL BIRTH		NO	NO	FEMALE	9	9	3185	NO	NO	NO	NO	YES	NO	NO	NO	NO	NO	30,43	NO	NO	YES
C-SECTION	NO	NO	MALE	5	9	2105	YES	NO	NO	NO	YES	NO	NO	NO	NO	YES	31,00	NO	NO	YES	
C-SECTION	YES	NO	FEMALE	8	8	2400	YES	YES	NO	NO	YES	NO	NO	NO	NO	NO	31,29	NO	NO	NO	
C-SECTION	YES	NO	MALE	9	10	3150	NO	NO	NO	NO	YES	NO	NO	NO	NO	NO	31,29	NO	YES	YES	
VAGINAL BIRTH		YES	NO	FEMALE	8	10	2250	NO	NO	NO	NO	YES	NO	NO	NO	NO	NO	31,43	NO	NO	NO
C-SECTION	YES	NO	FEMALE	8	9	3805	NO	NO	YES	NO	YES	NO	NO	NO	NO	NO	31,43	NO	NO	NO	
VAGINAL BIRTH		NO	NO	MALE	8	9	2970	NO	NO	NO	NO	YES	NO	YES	NO	NO	NO	31,43	NO	NO	YES
VAGINAL BIRTH		YES	NO	FEMALE			1800		NO	NO	NO	YES	NO	NO	YES	NO	NO	31,57	NO	NO	NO
C-SECTION	YES	NO	FEMALE	3	6	2110	YES	NO	NO	NO	YES	NO	NO	NO	NO	YES	31,57	NO	NO	NO	
VAGINAL BIRTH		YES	NO	FEMALE	8	9	1750	YES	NO	NO	NO	YES	NO	NO	NO	NO	NO	31,71	NO	NO	YES
C-SECTION	NO	NO	FEMALE	6	9	2705	NO	NO	NO	NO	YES	NO	NO	NO	NO	YES	31,71	NO	NO	YES	
VAGINAL BIRTH		NO	NO	FEMALE	7	9	2885	NO	NO	NO	NO	YES	NO	NO	NO	NO	NO	31,86	NO	NO	YES
VAGINAL BIRTH		YES	NO	MALE	9	9	2240	YES	NO	NO	NO	YES	NO	NO	NO	NO	NO	31,86	NO	NO	YES
C-SECTION	YES	NO	MALE	4	9	2615	YES	NO	NO	NO	YES	NO	NO	NO	YES	YES	32,14	NO	NO	NO	
C-SECTION	NO	NO	MALE	8	8	2950	NO	NO	NO	NO	YES			NO	NO	NO	32,14	NO	NO	NO	
VAGINAL BIRTH		YES	NO	MALE	9	10	3190	NO	YES	NO	NO	YES	NO	NO	NO	NO	NO	32,43	NO	NO	NO
VAGINAL BIRTH		YES	NO	FEMALE	8	9	2820	YES	YES	NO	NO	YES	NO	NO	NO	NO	NO	32,43	NO	NO	NO
C-SECTION	YES	NO	FEMALE	7	9	2360	YES	YES	YES	YES	YES	NO	NO	NO	NO	NO	32,43	NO	NO	NO	
C-SECTION	YES	NO	MALE	9	9	1628	YES	YES	YES	YES	YES	NO	NO	NO	NO	NO	32,43	NO	NO	NO	
C-SECTION	YES	NO	MALE	9	9	1900	YES	NO	NO	NO	YES	NO	NO	NO	NO	NO	32,57	NO	NO	NO	
VAGINAL BIRTH		YES	NO	MALE	9	9	2850	NO	NO	NO	NO	YES	NO	NO	NO	YES	NO	32,57	NO	NO	YES
C-SECTION	YES	NO	FEMALE	8	9	2890	NO	YES	NO	NO	YES	NO	NO	NO	NO	NO	32,71	NO	NO	YES	
VAGINAL BIRTH		NO	YES	MALE	2	5	2470	NO	NO	NO	NO	YES	NO	NO	NO	NO	YES	32,71	NO	YES	YES
VAGINAL BIRTH		YES	YES	FEMALE	9	9	3075	NO	NO	NO	NO	YES	NO	NO	NO	NO	NO	32,86	NO	NO	NO
C-SECTION	YES	NO	MALE	9	10	2305	YES	NO	NO	NO	YES	NO	NO	NO	NO	NO	33,00	NO	NO	NO	
VAGINAL BIRTH		YES	NO	MALE	7	9	2880	NO	YES	NO	NO	YES	NO	NO	NO	NO	NO	33,00	NO	NO	YES
C-SECTION	YES	NO	FEMALE	0	2	1875	YES	NO	NO	NO	YES	NO	NO	YES	NO	YES	33,14	NO	NO	NO	
C-SECTION	NO	NO	MALE			3595	NO	NO	NO	NO	YES	NO	NO	NO	NO	NO	33,14	NO	NO	YES	
VAGINAL BIRTH		NO	NO	FEMALE	9	9	3685	NO	NO	NO	NO	YES	NO	NO	NO	NO	NO	33,29	NO	NO	YES
VAGINAL BIRTH		YES	NO	FEMALE	9	9	2840	NO	YES	NO	NO	YES	NO	NO	NO	NO	NO	33,43	NO	NO	NO
C-SECTION	YES	NO	MALE	7	9	1720	YES	YES	NO	YES	YES	NO	NO	NO	NO	NO	33,43	NO	NO	NO	
C-SECTION	YES	NO	MALE	8	9	2110	YES	YES	NO	YES	YES	NO	NO	NO	NO	NO	33,43	NO	NO	NO	
VAGINAL BIRTH		NO	NO	MALE	8	9	3400	NO	NO	NO	NO	YES	NO	NO	NO	NO	NO	33,43	NO	NO	YES
VAGINAL BIRTH		YES	NO	MALE	8	9	3240	NO	YES	NO	NO	YES	NO	NO	NO	NO	NO	34,00	NO	NO	NO
C-SECTION	YES	NO	FEMALE	9	10	3360	NO	NO	NO	NO	YES	NO	NO	NO	YES	NO	34,43	NO	NO	NO	
VAGINAL BIRTH		YES	NO	MALE	9	9	3345	NO	YES	NO	NO	YES	NO	NO	NO	NO	NO	34,57	NO	NO	NO
C-SECTION	YES	NO	MALE	9	10	3780	NO	YES	NO	NO	YES	NO	NO	NO	NO	NO	34,71	NO	NO	YES	
C-SECTION	NO	NO	MALE	9	10	3060	NO	NO	NO	NO	YES	NO	NO	NO	NO	NO	35,00	NO	NO	NO	
VAGINAL BIRTH		NO	NO	MALE	8	9	3065	NO	NO	NO	NO	YES	NO	NO	NO	NO	NO	35,29	NO	YES	YES
C-SECTION	NO	NO	MALE	9	9	3000	NO	NO	NO	NO	YES	NO	NO	NO	NO	NO	35,43	NO	YES	YES	
C-SECTION	NO	NO	FEMALE	9	9	2885	NO	NO	NO	NO	YES	NO	NO	NO	NO	NO	35,71	NO	NO	YES	
VAGINAL BIRTH		YES	NO	MALE	9	10	2995	NO	YES	NO	NO	YES	NO	NO	NO	NO	NO	36,14	NO	NO	YES

C-SECTIONYES	NO	FEMALE	8	9	2365	YES	YES	NO	NO	YES	NO	NO	NO	YES	NO	36,29	NO	NO	NO	
C-SECTIONYES	NO	MALE	7	9	2900	NO	YES	NO	NO	YES	NO	NO	NO	NO	NO	36,43	NO	NO	NO	
C-SECTIONNO	NO	MALE	8	9	2745	NO	NO	NO	NO	YES	NO	NO	NO	NO	NO	36,86	NO	NO	NO	
VAGINAL BIRTH	YES	NO	FEMALE	8	9	3060	NO	YES	NO	NO	YES	NO	NO	NO	NO	NO	37,00	NO	NO	NO
VAGINAL BIRTH	YES	NO	MALE	9	10	2460	NO	NO	NO	NO	YES	NO	NO	NO	NO	NO	37,43	NO	NO	NO
C-SECTIONYES	NO	MALE	5	9	3575	YES	YES	NO	NO	YES	NO	NO	NO	NO	YES	37,43	NO	NO	NO	
VAGINAL BIRTH	NO	NO	FEMALE	8	9	3555	NO	NO	NO	NO	YES	NO	NO	NO	NO	NO	37,43	NO	NO	YES
C-SECTIONYES	NO	MALE	3	9	3255	YES	YES	NO	NO	YES	NO	NO	NO	NO	YES	37,57	NO	NO	NO	
VAGINAL BIRTH	NO	NO	MALE	8	10	3540	NO	NO	NO	NO	YES	NO	NO	NO	NO	NO	37,71	NO	YES	NO
C-SECTIONYES	NO	MALE	9	10	4075	NO	NO	YES	NO	YES	NO	NO	NO	NO	NO	37,86	NO	NO	YES	
C-SECTIONYES	NO	MALE	8	9	3350	NO	YES	NO	NO	YES	NO	NO	NO	NO	NO	38,29	NO	YES	NO	
VAGINAL BIRTH	YES	NO	FEMALE	9	9	2705	NO	NO	NO	NO	YES	NO	NO	NO	NO	NO	38,29	NO	NO	YES
VAGINAL BIRTH	NO	NO	MALE	9	9	2115	NO	NO	NO	NO	YES	NO	NO	NO	NO	NO	5,00	NO	NO	YES
VAGINAL BIRTH	NO	NO	MALE	9	10	3340	NO	NO	NO	NO	YES	NO	NO	NO	NO	NO	5,14	NO	NO	YES
VAGINAL BIRTH	NO	NO	FEMALE			385	NO	NO	NO	NO	YES	NO	NO	YES	NO	NO	5,43	NO	NO	NO
C-SECTIONYES	NO	MALE	1	8	2290	YES	NO	NO	NO	YES	NO	NO	NO	NO	YES	5,71	NO	NO	NO	
C-SECTIONYES	NO	FEMALE	9	10	3570	NO	NO	NO	NO	YES	NO	NO	NO	YES	NO	5,71	NO	NO	NO	
VAGINAL BIRTH	YES	NO	FEMALE	9	10	3290	NO	NO	NO	NO	YES	NO	NO	NO	NO	NO	5,71	NO	NO	YES
VAGINAL BIRTH	NO	NO	MALE	8	10	3035	NO	NO	NO	NO	YES	NO	NO	NO	NO	NO	5,71	NO	NO	YES
VAGINAL BIRTH	YES	YES	MALE	2	7	3080	YES	NO	YES	NO	YES	NO	NO	NO	NO	YES	5,71	NO	NO	YES
VAGINAL BIRTH	YES	NO	FEMALE	10	10	4005	NO	YES	NO	NO	YES	NO	NO	NO	NO	NO	5,86	NO	NO	NO
VAGINAL BIRTH	YES	NO	FEMALE	8	9	3260	NO	NO	YES	NO	YES	NO	NO	NO	NO	NO	5,86	NO	NO	NO
VAGINAL BIRTH	YES	NO	FEMALE	9	9	3295	NO	NO	NO	NO	YES	NO	NO	NO	YES	NO	5,86	NO	YES	NO
VAGINAL BIRTH	YES	NO	MALE	9	9	2685	NO	YES	NO	NO	YES	NO	NO	NO	NO	NO	5,86	NO	NO	NO
C-SECTIONYES	NO	MALE	9	10	2255	NO	NO	NO	NO	YES	NO	NO	NO	NO	NO	5,86	NO	NO	NO	
VAGINAL BIRTH	YES	NO	FEMALE			630		NO	NO	NO	YES	NO	NO	YES	NO	NO	6,00	NO	NO	NO
C-SECTIONYES	NO	FEMALE	9	10	3295	NO	YES	NO	NO	YES	NO	NO	NO	NO	NO	6,00	NO	NO	NO	
C-SECTIONYES	NO	MALE	7	8	2890	NO	YES	NO	NO	YES	NO	NO	NO	NO	NO	6,00	NO	NO	NO	
C-SECTIONYES	NO	MALE	8	9	1960	NO	YES	NO	YES	YES	NO	NO	NO	NO	NO	6,00	NO	NO	NO	
C-SECTIONYES	NO	FEMALE	8	9	2030	NO	YES	NO	YES	YES	NO	NO	NO	NO	NO	6,00	NO	NO	NO	
C-SECTIONYES	NO	FEMALE	8	9	2930	NO	YES	NO	NO	YES	NO	NO	NO	YES	NO	6,00	NO	NO	NO	
VAGINAL BIRTH	YES	NO	MALE	10	10	2900	NO	NO	NO	NO	YES	NO	NO	NO	NO	NO	6,00	NO	NO	YES
VAGINAL BIRTH	NO	YES	UNDETERMINATED		8	9	2330	NO	NO	NO	NO	YES	NO	NO	NO	NO	NO	6,00	NO	YES
YES																				
VAGINAL BIRTH	YES	NO	MALE	6	8	2315	NO	YES	NO	NO	YES	NO	NO	NO	NO	YES	6,00	NO	NO	YES
VAGINAL BIRTH	NO	NO	FEMALE	9	9	3665	NO	NO	NO	NO	YES	NO	NO	NO	NO	NO	6,00	NO	NO	YES
VAGINAL BIRTH	NO	NO	MALE	8	9	2635	NO	NO	NO	NO	YES	NO	NO	NO	NO	NO	6,00	NO	NO	YES
VAGINAL BIRTH	YES	NO	MALE	9	9	3920	NO	NO	NO	NO	YES	NO	NO	NO	NO	NO	6,00	NO	NO	YES
VAGINAL BIRTH	YES	NO	FEMALE	0	0	2950		NO	NO	NO	YES	NO	NO	YES	NO	YES	6,14	NO	NO	NO
C-SECTIONYES	NO	MALE	9	10	3225	NO	NO	NO	NO	YES	NO	NO	NO	YES	NO	6,14	NO	NO	NO	
C-SECTIONYES	NO	MALE	8	9	2350	NO	NO	NO	YES	YES	NO	NO	NO	NO	NO	6,14	NO	NO	NO	
C-SECTIONYES	NO	MALE	9	10	2425	NO	NO	NO	YES	YES	NO	NO	NO	NO	NO	6,14	NO	NO	NO	
C-SECTIONYES	NO	MALE	8	9	1570	YES	YES	NO	NO	YES	NO	NO	NO	NO	NO	6,14	NO	NO	NO	
C-SECTIONYES	NO	FEMALE	9	10	3290	NO	NO	NO	NO	YES	NO	NO	NO	NO	NO	6,14	NO	NO	NO	
VAGINAL BIRTH	YES	NO	MALE	9	10	3020	NO	NO	NO	NO	YES	NO	NO	NO	NO	NO	6,29	NO	YES	NO
VAGINAL BIRTH	YES	NO	MALE	8	9	3490	NO	NO	NO	NO	YES	NO	NO	NO	NO	NO	6,29	NO	NO	NO
C-SECTIONNO	NO	FEMALE	8	9	3740	NO	NO	NO	NO	YES	NO	NO	NO	NO	NO	6,29	NO	YES	NO	
C-SECTIONYES	NO	FEMALE	9	10	2430	NO	NO	NO	NO	YES	NO	NO	NO	NO	NO	6,29	NO	NO	NO	
C-SECTIONYES	NO	MALE	6	8	2120	YES	YES	NO	NO	YES	NO	NO	NO	NO	YES	6,29	NO	NO	NO	
C-SECTIONNO	NO	MALE	8	9	3800	NO	NO	NO	NO	YES	NO	NO	NO	NO	NO	6,29	NO	NO	NO	
C-SECTIONYES	NO	FEMALE	8	9	3135	NO	NO	NO	NO	YES	NO	NO	NO	YES	NO	6,29	NO	NO	YES	
VAGINAL BIRTH	YES	NO	MALE	9	10	2640	NO	NO	NO	NO	YES	NO	NO	NO	NO	NO	6,29	NO	NO	YES
VAGINAL BIRTH	YES	NO	FEMALE	9	10	2805	NO	NO	NO	NO	YES	NO	NO	NO	NO	NO	6,43	NO	NO	NO

C-SECTION	YES	NO	FEMALE	9	10	3490	NO	NO	YES	NO	YES	NO	NO	NO	NO	NO	6,43	NO	YES	NO	
C-SECTION	YES	NO	FEMALE	8	9	2915	NO	YES	NO	NO	YES	NO	NO	NO	NO	NO	6,43	NO	NO	NO	
C-SECTION	YES	NO	FEMALE	8	10	2595	NO	NO	NO	NO	YES	NO	NO	NO	NO	NO	6,43	NO	NO	NO	
C-SECTION	YES	NO	MALE	9	10	3570	NO	NO	YES	NO	YES	NO	NO	NO	NO	NO	6,43	NO	NO	NO	
C-SECTION	NO	NO	MALE	8	9	3340	NO	NO	NO	NO	YES	NO	NO	NO	NO	NO	6,43	NO	NO	NO	
C-SECTION	NO	YES	MALE	8	9	4050	NO	NO	NO	NO	YES	NO	NO	NO	NO	NO	6,43	NO	NO	NO	
C-SECTION	YES	NO	MALE	6	9	1750	YES	NO	NO	NO	YES	NO	NO	NO	NO	YES	6,43	NO	NO	NO	
VAGINAL BIRTH		YES	NO	MALE	9	10	2345	NO	NO	NO	NO	YES	NO	NO	NO	NO	NO	6,43	NO	NO	YES
VAGINAL BIRTH		NO	NO	FEMALE	8	9	3705	NO	NO	NO	NO	YES	NO	NO	NO	NO	NO	6,43	NO	NO	YES
C-SECTION	YES	NO	MALE	8	9	2080	NO	NO	NO	YES	YES	NO	NO	NO	NO	NO	6,43	NO	NO	YES	
C-SECTION	YES	NO	MALE	8	10	2250	NO	NO	NO	YES	YES	NO	NO	NO	NO	NO	6,43	NO	NO	YES	
C-SECTION	NO	NO	FEMALE	9	9	3740	NO	NO	NO	NO	YES	NO	NO	NO	NO	NO	6,43	NO	NO	YES	
VAGINAL BIRTH		NO	NO	MALE	6	8	3475	NO	NO	NO	NO	YES	NO	NO	NO	NO	YES	6,43	NO	YES	YES
VAGINAL BIRTH		YES	NO	FEMALE	9	10	2940	NO	NO	NO	NO	YES	NO	NO	NO	YES	NO	6,43	NO	YES	YES
VAGINAL BIRTH		YES	NO	FEMALE	5	9	875	YES	NO	NO	NO	YES	NO	NO	NO	NO	YES	6,43	NO	NO	YES
VAGINAL BIRTH		YES	YES	MALE	5	9	2980	YES	YES	NO	NO	YES	NO	NO	NO	NO	YES	6,57	NO	YES	NO
VAGINAL BIRTH		YES	NO	MALE	0	1	495	NO	NO	NO	NO	YES	NO	NO	YES	NO	NO	6,57	NO	NO	NO
C-SECTION	YES	NO	FEMALE	8	8	2460	NO	NO	NO	YES	YES	NO	NO	NO	NO	NO	6,57	NO	NO	NO	
C-SECTION	YES	NO	FEMALE	5	9	2215	NO	NO	NO	YES	YES	NO	NO	NO	NO	YES	6,57	NO	NO	NO	
C-SECTION	NO	NO	FEMALE	8	9	2495	NO	NO	NO	NO	YES	NO	NO	NO	NO	NO	6,57	NO	NO	NO	
C-SECTION	NO	NO	MALE	9	10	3015	NO	NO	NO	NO	YES	NO	NO	NO	NO	NO	6,57	NO	NO	NO	
C-SECTION	YES	NO	MALE	8	9	3230	NO	NO	NO	NO	YES	NO	NO	NO	NO	NO	6,57	NO	NO	NO	
C-SECTION	NO	NO	MALE	7	9	3730	NO	NO	NO	NO	YES	NO	NO	NO	NO	NO	6,57	NO	NO	NO	
C-SECTION	YES	NO	MALE	8	9	3390	NO	NO	NO	NO	YES	NO	NO	NO	NO	NO	6,57	NO	NO	NO	
C-SECTION	YES	NO	MALE	9	10	3690	NO	NO	NO	NO	YES	NO	NO	NO	YES	NO	6,57	NO	NO	NO	
C-SECTION	YES	NO	FEMALE	8	9	2975	NO	YES	YES	NO	YES	NO	NO	NO	NO	NO	6,57	NO	NO	NO	
C-SECTION	YES	NO	MALE	8	9	4640	NO	YES	NO	NO	YES	NO	NO	NO	NO	NO	6,57	NO	NO	NO	
VAGINAL BIRTH		NO	YES	FEMALE	9	9	3250	NO	NO	NO	NO	YES	NO	NO	NO	NO	NO	6,57	NO	YES	YES
VAGINAL BIRTH		NO	YES	FEMALE	7	10	2970	NO	NO	NO	NO	YES	NO	NO	NO	NO	NO	6,57	NO	YES	YES
C-SECTION	NO	NO	FEMALE	8	9	4105	NO	NO	NO	NO	YES	NO	NO	NO	NO	NO	6,57	NO	NO	YES	
VAGINAL BIRTH		NO	NO	MALE	10	10	2865	NO	NO	NO	NO	YES	NO	NO	NO	NO	NO	6,57	NO	YES	YES
VAGINAL BIRTH		YES	NO	FEMALE	9	9	2920	NO	NO	NO	NO	YES	NO	NO	NO	YES	NO	6,57	NO	NO	YES
VAGINAL BIRTH		YES	NO	FEMALE	9	10	2425	NO	NO	NO	NO	YES	NO	NO	NO	NO	NO	6,71	NO	NO	NO
VAGINAL BIRTH		YES	NO	FEMALE	9	10	3805	NO	YES	NO	NO	YES	NO	NO	NO	NO	NO	6,71	NO	NO	NO
VAGINAL BIRTH		YES	NO	MALE	9	9	3570	NO	YES	NO	NO	YES	NO	NO	NO	NO	NO	6,71	NO	NO	NO
C-SECTION	YES	NO	FEMALE	9	9	2130	NO	NO	NO	NO	YES	NO	NO	NO	NO	NO	6,71	NO	NO	YES	
VAGINAL BIRTH		NO	NO	FEMALE	8	9	3740	NO	NO	NO	NO	YES	NO	NO	NO	NO	NO	6,71	NO	NO	YES
VAGINAL BIRTH		YES	NO	MALE	8	8	3455	NO	NO	NO	NO	YES	NO	NO	NO	NO	NO	6,71	NO	NO	YES
C-SECTION	NO	NO	FEMALE	4	6	2585	YES	NO	NO	NO	YES	NO	NO	NO	NO	YES	6,86	NO	YES	NO	
VAGINAL BIRTH		YES	NO	FEMALE	9	9	2540	NO	YES	NO	NO	YES	NO	NO	NO	NO	NO	6,86	NO	NO	NO
VAGINAL BIRTH		YES	NO	MALE	9	9	2950	NO	YES	NO	NO	YES	NO	NO	NO	NO	NO	6,86	NO	NO	NO
C-SECTION	YES	NO	MALE	9	9	3925	NO	NO	YES	NO	YES	NO	NO	NO	NO	NO	6,86	NO	NO	NO	
C-SECTION	YES	NO	FEMALE	10	10	2820	NO	YES	NO	NO	YES	NO	NO	NO	NO	NO	6,86	NO	NO	NO	
C-SECTION	YES	NO	MALE	6	7	1030	YES	NO	NO	NO	YES	NO	NO	YES	NO	YES	6,86	NO	NO	NO	
VAGINAL BIRTH		NO	NO	FEMALE	8	9	3765	NO	NO	NO	NO	YES	NO	NO	NO	NO	NO	6,86	NO	NO	YES
VAGINAL BIRTH		NO	NO	FEMALE	8	9	3010	NO	NO	NO	NO	YES	NO	NO	NO	NO	NO	6,86	NO	NO	YES
VAGINAL BIRTH		YES	NO	MALE	9	9	3365	NO	NO	NO	NO	YES	NO	NO	NO	NO	NO	6,86	NO	NO	YES
C-SECTION	YES	NO	MALE	9	10	3110	NO	NO	NO	NO	YES	NO	NO	NO	NO	NO	6,86	NO	NO	YES	
VAGINAL BIRTH		YES	NO	MALE	10	10	3140	NO	NO	NO	NO	YES	NO	NO	NO	NO	NO	6,86	NO	NO	YES
VAGINAL BIRTH		NO	NO	FEMALE	8	10	2580	NO	NO	NO	NO	YES	NO	NO	NO	NO	NO	6,86	NO	YES	YES
VAGINAL BIRTH		NO	NO	FEMALE	9	10	3380	NO	NO	NO	NO	YES	NO	NO	NO	NO	NO	6,86	NO	YES	YES
C-SECTION	YES	NO	FEMALE	0	0	1600	YES	NO	NO	NO	YES	NO	NO	YES	NO	YES	7,00	NO	NO	NO	
C-SECTION	YES	NO	FEMALE	8	9	3185	NO	NO	NO	NO	YES	NO	NO	NO	NO	NO	7,00	NO	NO	NO	

C-SECTION	NO	NO	MALE	8	9	3525	NO	NO	NO	NO	YES	NO	NO	NO	NO	NO	7,00	NO	NO	NO	
C-SECTION	YES	NO	MALE	5	9	2640	NO	NO	NO	NO	YES	NO	NO	NO	NO	YES	7,00	NO	NO	NO	
C-SECTION	YES	NO	FEMALE	0	0	2995		NO	NO	NO	YES	NO	NO	YES	NO	NO	7,00	NO	NO	NO	
C-SECTION	YES	NO	FEMALE	7	9	3115	NO	YES	NO	NO	YES	NO	NO	NO	NO	NO	7,00	NO	NO	NO	
C-SECTION	NO	NO	FEMALE	8	9	3815	NO	NO	NO	NO	YES	NO	NO	NO	NO	NO	7,00	NO	NO	NO	
VAGINAL BIRTH		YES	NO	FEMALE	7	9	2160	NO	NO	NO	YES	YES	NO	NO	NO	NO	NO	7,00	NO	NO	YES
VAGINAL BIRTH		YES	NO	FEMALE	8	9	2210	NO	NO	NO	YES	YES	NO	NO	NO	NO	NO	7,00	NO	NO	YES
VAGINAL BIRTH		NO	NO	FEMALE	9	10	3155	NO	NO	NO	NO	YES	NO	NO	NO	NO	NO	7,00	NO	NO	YES
VAGINAL BIRTH		YES	NO	MALE	8	9	3780	NO	NO	NO	NO	YES	NO	NO	NO	NO	NO	7,00	NO	NO	YES
VAGINAL BIRTH		YES	NO	FEMALE	9	10	3180	NO	NO	NO	NO	YES	NO	NO	NO	NO	NO	7,00	NO	NO	YES
C-SECTION	YES	NO	MALE	2	9	3310	NO	NO	NO	NO	YES	NO	NO	NO	NO	YES	7,00	NO	NO	YES	
VAGINAL BIRTH		NO	NO	MALE	9	10	3220	NO	NO	NO	NO	YES	NO	NO	NO	NO	NO	7,00	NO	NO	YES
VAGINAL BIRTH		NO	YES	FEMALE	9	9	3165	NO	NO	NO	NO	YES	NO	NO	NO	NO	NO	7,00	NO	YES	YES
C-SECTION	YES	NO	FEMALE	2	8	2765	YES	NO	NO	YES	YES	NO	NO	NO	NO	YES	7,00	NO	YES	YES	
C-SECTION	YES	NO	FEMALE	4	9	2975	YES	NO	NO	YES	YES	NO	NO	NO	NO	YES	7,00	NO	YES	YES	
VAGINAL BIRTH		NO	NO	FEMALE	8	9	3180	NO	NO	NO	NO	YES	NO	NO	NO	NO	NO	7,00	NO	NO	YES
VAGINAL BIRTH		YES	NO	FEMALE	4	8	1220	YES	NO	NO	NO	YES	NO	NO	NO	NO	YES	7,00	NO	NO	YES
C-SECTION	YES	NO	FEMALE	9	10	2925	NO	YES	NO	NO	YES	NO	NO	NO	NO	NO	7,00	NO	NO	YES	
C-SECTION	YES	NO	MALE	8	9	2655	NO	YES	NO	NO	YES	NO	NO	NO	NO	NO	7,00	NO	NO	YES	
VAGINAL BIRTH		NO	NO	MALE	9	9	2515	NO	NO	NO	NO	YES	NO	NO	NO	NO	NO	7,00	NO	NO	YES
C-SECTION	YES	NO	FEMALE	9	10	2625	NO	NO	NO	NO	YES	NO	NO	NO	NO	NO	7,14	NO	NO	NO	
C-SECTION	NO	NO	FEMALE	9	9	3960	NO	NO	NO	NO	YES	NO	NO	NO	NO	NO	7,14	NO	NO	NO	
C-SECTION	YES	NO	MALE	9	9	3155	NO	NO	NO	NO	YES	NO	NO	NO	NO	NO	7,14	NO	NO	NO	
C-SECTION	NO	NO	FEMALE	8	8	3355	NO	NO	NO	NO	YES	NO	NO	NO	NO	NO	7,14	NO	NO	NO	
C-SECTION	YES	NO	MALE	9	10	3850	NO	NO	YES	NO	YES	NO	NO	NO	NO	NO	7,14	NO	NO	NO	
C-SECTION	YES	NO	MALE	8	10	3460	NO	NO	YES	NO	YES	NO	NO	NO	NO	NO	7,14	NO	NO	YES	
VAGINAL BIRTH		NO	NO	FEMALE	7	8	2715	NO	NO	NO	NO	YES	NO	NO	NO	NO	NO	7,14	NO	YES	YES
C-SECTION	NO	NO	MALE	9	9	2970	NO	NO	NO	NO	YES	NO	NO	NO	NO	NO	7,14	NO	YES	YES	
C-SECTION	YES	NO	FEMALE	8	8	3430	NO	YES	YES	NO	YES	NO	NO	NO	NO	NO	7,14	NO	NO	YES	
VAGINAL BIRTH		YES	YES	MALE	7	9	3300	NO	NO	NO	NO	YES	NO	NO	NO	NO	NO	7,14	NO	NO	YES
VAGINAL BIRTH		YES	NO	MALE	0	0	475		NO	NO	YES	YES	NO	NO	YES	NO	NO	7,29	NO	NO	NO
VAGINAL BIRTH		YES	NO	MALE	1	1	475	NO	NO	NO	YES	YES	NO	NO	YES	NO	NO	7,29	NO	NO	NO
C-SECTION	NO	NO	MALE	5	9	945	YES	NO	NO	NO	YES	YES	NO	NO	NO	YES	7,29	NO	NO	NO	
C-SECTION	NO	NO	MALE	9	10	3555	NO	NO	NO	NO	YES	NO	NO	NO	NO	NO	7,29	NO	NO	NO	
C-SECTION	NO	NO	MALE	8	9	4800	NO	NO	NO	NO	YES	NO	NO	NO	NO	NO	7,29	NO	NO	NO	
C-SECTION	YES	NO	FEMALE	8	9	2925	NO	NO	NO	NO	YES	NO	NO	NO	NO	NO	7,29	NO	NO	NO	
VAGINAL BIRTH		NO	NO	MALE	9	10	3430	NO	NO	NO	NO	YES	NO	NO	NO	NO	NO	7,29	NO	NO	YES
VAGINAL BIRTH		YES	NO	MALE	8	9	2540	NO	NO	NO	NO	YES			NO	NO	NO	7,29	NO	NO	YES
VAGINAL BIRTH		NO	NO	MALE	9	10	3210	NO	NO	NO	NO	YES	NO	NO	NO	NO	NO	7,29	NO	NO	YES
VAGINAL BIRTH		NO	NO	MALE	9	10	4400	NO	NO	NO	NO	YES	NO	NO	NO	NO	NO	7,29	NO	NO	YES
VAGINAL BIRTH		YES	NO	FEMALE	9	9	2165	NO	NO	NO	NO	YES	NO	NO	NO	NO	NO	7,29	NO	NO	YES
VAGINAL BIRTH		YES	NO	MALE	9	9	3205	NO	NO	NO	NO	YES	NO	NO	NO	YES	NO	7,43	NO	YES	NO
C-SECTION	YES	NO	MALE	9	10	3125	NO	NO	NO	NO	YES	NO	NO	NO	NO	NO	7,43	NO	YES	NO	
C-SECTION	YES	NO	FEMALE	8	9	2745	YES	YES	YES	NO	YES	NO	NO	NO	NO	NO	7,43	NO	NO	NO	
C-SECTION	YES	NO	FEMALE	7	8	2445	NO	NO	NO	NO	YES	NO	NO	NO	NO	NO	7,43	NO	NO	NO	
C-SECTION	YES	NO	MALE	9	9	2605	NO	YES	NO	NO	YES	NO	NO	NO	NO	NO	7,43	NO	NO	NO	
C-SECTION	YES	NO						YES	NO	NO	YES	NO	NO	YES	NO	NO	7,43	NO	NO	NO	
C-SECTION	YES	NO	MALE	6	9	2300	YES	NO	YES	NO	YES	NO	NO	YES	NO	YES	7,43	NO	NO	NO	
C-SECTION	YES	NO	FEMALE	7	9	1325	NO	YES	NO	NO	YES	NO	NO	NO	NO	NO	7,43	NO	NO	NO	
C-SECTION	YES	NO	FEMALE	9	9	2720	NO	YES	NO	NO	YES	NO	NO	NO	NO	NO	7,43	NO	NO	NO	
C-SECTION	YES	NO	MALE	8	9	3380	NO	NO	YES	NO	YES	NO	NO	NO	NO	NO	7,43	NO	NO	NO	
C-SECTION	NO	NO	MALE	7	9	3925	NO	NO	NO	NO	YES	NO	NO	NO	NO	NO	7,43	NO	NO	NO	
C-SECTION	YES	NO	FEMALE	9	9	3435	NO	NO	NO	NO	YES	NO	NO	NO	NO	NO	7,43	NO	NO	NO	

C-SECTIONYES	NO	MALE	7	8	2780	NO	NO	NO	NO	YES	NO	NO	NO	NO	NO	7,43	NO	YES	YES	
VAGINAL BIRTH	YES	YES	FEMALE	9	9	2980	NO	NO	NO	NO	YES	NO	NO	NO	YES	NO	7,43	NO	NO	YES
VAGINAL BIRTH	NO	NO	MALE	8	9	2830	NO	NO	NO	NO	YES	NO	NO	NO	NO	NO	7,43	NO	YES	YES
VAGINAL BIRTH	NO	NO	MALE	9	10	2850	NO	NO	NO	NO	YES	NO	NO	NO	NO	NO	7,43	NO	NO	YES
VAGINAL BIRTH	NO	NO	MALE	9	9	3695	NO	NO	NO	NO	YES	NO	NO	NO	NO	NO	7,43	NO	NO	YES
VAGINAL BIRTH	NO	NO	MALE	9	10	3265	NO	NO	NO	NO	YES	NO	NO	NO	NO	NO	7,43	NO	NO	YES
VAGINAL BIRTH	YES	NO	MALE	9	9	3490	NO	NO	NO	NO	YES	NO	NO	NO	NO	NO	7,43	NO	NO	YES
C-SECTIONNO	NO	MALE	8	9	3515	NO	NO	NO	NO	YES	NO	NO	NO	NO	NO	7,43	NO	YES	YES	
VAGINAL BIRTH	YES	NO	MALE	9	9	4130	NO	YES	NO	NO	YES	NO	NO	NO	NO	NO	7,43	NO	YES	YES
C-SECTIONYES	NO	MALE	8	9	3030	NO	NO	NO	NO	YES	NO	NO	NO	NO	NO	7,43	NO	NO	YES	
VAGINAL BIRTH	YES	NO	MALE	9	10	3815	NO	YES	NO	NO	YES	NO	NO	NO	NO	NO	7,57	NO	NO	NO
VAGINAL BIRTH	YES	NO	MALE	9	9	3595	NO	YES	NO	NO	YES	NO	NO	NO	NO	NO	7,57	NO	YES	NO
VAGINAL BIRTH	NO	NO	FEMALE	8	9	3795	NO	NO	NO	NO	YES	NO	NO	NO	NO	NO	7,57	NO	YES	NO
C-SECTIONYES	NO	MALE	9	10	2900	NO	YES	NO	NO	YES	NO	NO	NO	NO	NO	7,57	NO	NO	NO	
VAGINAL BIRTH	YES	YES	FEMALE	8	9	3125	NO	YES	NO	NO	YES	NO	NO	NO	NO	NO	7,57	NO	YES	NO
C-SECTIONYES	NO	MALE	9	9	3295	NO	NO	YES	NO	YES	NO	NO	NO	NO	NO	7,57	NO	NO	NO	
C-SECTIONYES	NO	FEMALE	9	9	3635	NO	NO	YES	NO	YES	NO	NO	NO	NO	NO	7,57	NO	NO	NO	
VAGINAL BIRTH	YES	NO	MALE	5	8	3405	NO	NO	NO	NO	YES	NO	NO	NO	NO	YES	7,57	NO	YES	YES
VAGINAL BIRTH	NO	NO	MALE	8	9	3805	NO	NO	NO	NO	YES	NO	NO	NO	NO	NO	7,57	NO	NO	YES
VAGINAL BIRTH	NO	NO	FEMALE	8	8	2505	NO	NO	NO	NO	YES	NO	NO	NO	NO	NO	7,57	NO	NO	YES
VAGINAL BIRTH	YES	NO	FEMALE	8	9	2190	NO	NO	NO	NO	YES	NO	NO	NO	NO	NO	7,57	NO	NO	YES
VAGINAL BIRTH	NO	NO	FEMALE	8	9	2265	NO	NO	NO	NO	YES	NO	NO	NO	NO	NO	7,57	NO	NO	YES
VAGINAL BIRTH	NO	NO	MALE	9	10	3050	NO	NO	NO	NO	YES	NO	NO	NO	NO	NO	7,57	NO	YES	YES
VAGINAL BIRTH	NO	NO	FEMALE	6	7	3705	NO	NO	NO	NO	YES	NO	NO	NO	NO	YES	7,57	NO	NO	YES
VAGINAL BIRTH	NO	NO	FEMALE	7	9	3030	NO	NO	NO	NO	YES	NO	NO	NO	NO	NO	7,57	NO	NO	YES
C-SECTIONNO	NO	FEMALE	5	8	3500	YES	NO	NO	NO	YES	NO	NO	NO	NO	YES	7,71	NO	NO	NO	
VAGINAL BIRTH	YES	NO	MALE	8	10	4035	YES	YES	NO	NO	YES	NO	NO	NO	NO	NO	7,71	NO	YES	NO
VAGINAL BIRTH	YES	NO	MALE	8	9	2290	YES	YES	NO	NO	YES	NO	NO	NO	NO	NO	7,71	NO	YES	NO
VAGINAL BIRTH	YES	NO	FEMALE	8	9	2710	NO	NO	NO	NO	YES	NO	NO	NO	YES	NO	7,71	NO	NO	NO
VAGINAL BIRTH	YES	NO	FEMALE	9	10	3185	NO	YES	NO	NO	YES	NO	NO	NO	NO	NO	7,71	NO	YES	NO
VAGINAL BIRTH	YES	NO	MALE	4	8	3420	NO	NO	YES	NO	YES	NO	NO	NO	NO	YES	7,71	NO	NO	NO
VAGINAL BIRTH	YES	NO	FEMALE	0	0	800		YES	NO	NO	YES	NO	NO	YES	NO	NO	7,71	NO	NO	NO
C-SECTIONYES	NO	MALE	7	9	1200	NO	YES	NO	NO	YES	NO	NO	NO	NO	NO	7,71	NO	NO	NO	
C-SECTIONYES	NO	MALE	8	9	2740	YES	NO	NO	NO	YES	NO	NO	NO	NO	NO	7,71	NO	NO	NO	
C-SECTIONYES	NO	FEMALE	9	9	3540	NO	NO	YES	NO	YES	NO	NO	NO	NO	NO	7,71	NO	NO	NO	
C-SECTIONYES	NO	MALE	9	10	2530	NO	NO	NO	NO	YES	NO	NO	NO	NO	NO	7,71	NO	NO	NO	
VAGINAL BIRTH	NO	NO	MALE	8	9	3820	NO	NO	NO	NO	YES	NO	NO	NO	NO	NO	7,71	NO	NO	YES
VAGINAL BIRTH	YES	NO	FEMALE	7	8	525	YES	NO	NO	NO	YES	NO	NO	NO	NO	NO	7,71	NO	NO	YES
VAGINAL BIRTH	YES	NO	FEMALE	8	9	2375	NO	NO	NO	NO	YES	NO	NO	NO	NO	NO	7,71	NO	NO	YES
C-SECTIONYES	NO	FEMALE	8	9	3020	NO	NO	NO	NO	YES	NO	NO	NO	NO	NO	7,71	NO	NO	YES	
VAGINAL BIRTH	NO	NO	MALE	9	10	3895	NO	NO	NO	NO	YES	NO	NO	NO	NO	NO	7,71	NO	NO	YES
C-SECTIONYES	NO	FEMALE	8	9	2585	NO	NO	NO	NO	YES	NO	NO	NO	YES	NO	7,86	NO	NO	NO	
C-SECTIONNO	NO	MALE	8	9	3770	NO	NO	NO	NO	YES	NO	NO	NO	NO	NO	7,86	NO	YES	NO	
VAGINAL BIRTH	NO	NO	FEMALE	9	10	2170	NO	NO	NO	NO	YES	NO	NO	NO	NO	NO	7,86	NO	NO	YES
VAGINAL BIRTH	YES	NO	FEMALE	8	9	1785	YES	NO	NO	YES	YES	NO	NO	NO	NO	NO	7,86	NO	NO	YES
VAGINAL BIRTH	NO	NO	FEMALE	9	9	3490	NO	NO	NO	NO	YES	NO	NO	NO	NO	NO	7,86	NO	NO	YES
VAGINAL BIRTH	YES	NO	MALE	1	2	1580	YES	NO	NO	NO	YES	NO	NO	NO	YES	YES	7,86	NO	NO	YES
VAGINAL BIRTH	YES	NO	FEMALE	7	9	3410	NO	NO	NO	NO	YES	NO	NO	NO	NO	NO	8,00	NO	NO	NO
VAGINAL BIRTH	NO	NO	FEMALE	8	10	2610	NO	NO	NO	NO	YES	NO	NO	NO	NO	NO	8,00	NO	NO	NO
VAGINAL BIRTH	YES	NO	FEMALE	9	9	2230	NO	NO	NO	NO	YES	NO	NO	NO	NO	NO	8,00	NO	YES	NO
C-SECTIONYES	NO	FEMALE	10	10	3365	NO	YES	NO	NO	YES	NO	NO	NO	NO	NO	8,00	NO	NO	NO	
C-SECTIONYES	NO	FEMALE	8	9	3750	NO	NO	YES	NO	YES	NO	NO	NO	NO	NO	8,00	NO	NO	NO	
VAGINAL BIRTH	NO	YES	MALE	6	9	2905	NO	NO	NO	NO	YES	NO	NO	NO	NO	YES	8,00	NO	YES	NO

C-SECTIONYES	NO	MALE	7	9	2040	YES	YES	NO	NO	YES	NO	NO	NO	NO	NO	8,00	NO	NO	NO	
C-SECTIONYES	NO	FEMALE	8	9	4380	NO	NO	YES	NO	YES	NO	NO	NO	NO	NO	8,00	NO	NO	NO	
C-SECTIONYES	NO	FEMALE	1	7	1540	YES	YES	NO	NO	YES	NO	YES	NO	NO	YES	8,00	NO	NO	NO	
C-SECTIONYES	NO	FEMALE	8	9	3125	NO	NO	NO	NO	YES	NO	NO	NO	NO	NO	8,00	NO	NO	NO	
C-SECTIONYES	NO	MALE	3	10	2110	YES	NO	NO	NO	YES	NO	NO	NO	YES	YES	8,00	NO	NO	YES	
VAGINAL BIRTH	NO	NO	FEMALE	5	8	2160	NO	NO	NO	NO	YES	NO	NO	NO	NO	YES	8,00	NO	NO	YES
VAGINAL BIRTH	YES	NO	MALE	8	9	3365	NO	NO	NO	NO	YES	NO	NO	NO	NO	NO	8,00	NO	NO	YES
VAGINAL BIRTH	NO	NO	MALE	8	9	3540	NO	NO	NO	NO	YES	NO	NO	NO	NO	NO	8,00	NO	NO	YES
VAGINAL BIRTH	NO	NO	MALE	8	9	4080	NO	NO	NO	NO	YES	NO	NO	NO	NO	NO	8,00	NO	YES	YES
VAGINAL BIRTH	YES	NO	MALE	4	9	1975	YES	NO	NO	NO	YES	NO	NO	NO	YES	YES	8,00	NO	YES	YES
VAGINAL BIRTH	YES	NO	MALE	9	9	2020	YES	YES	NO	NO	YES	NO	NO	NO	NO	NO	8,00	NO	NO	YES
VAGINAL BIRTH	NO	NO	FEMALE	8	9	2965	NO	NO	NO	NO	YES	NO	NO	NO	NO	NO	8,00	NO	YES	YES
VAGINAL BIRTH	YES	NO	FEMALE	9	9	2525	NO	NO	NO	NO	YES	NO	NO	NO	NO	NO	8,00	NO	NO	YES
VAGINAL BIRTH	NO	NO	MALE	9	10	3550	NO	NO	NO	NO	YES	NO	NO	NO	NO	NO	8,00	NO	NO	YES
VAGINAL BIRTH	NO	NO	MALE	9	10	3400	NO	NO	NO	NO	YES	NO	NO	NO	NO	NO	8,00	NO	NO	YES
VAGINAL BIRTH	YES	NO	MALE	9	9	2875	NO	NO	NO	NO	YES	NO	NO	NO	NO	NO	8,00	NO	NO	YES
VAGINAL BIRTH	NO	NO	MALE	9	10	3010	NO	NO	NO	NO	YES	NO	NO	NO	NO	NO	8,00	NO	NO	YES
VAGINAL BIRTH	YES	NO	MALE	0	0	585		NO	NO	NO	YES	NO	NO	YES	NO	NO	8,14	NO	NO	NO
VAGINAL BIRTH	YES	NO	FEMALE	9	9	2460	NO	NO	NO	NO	YES	NO	NO	NO	NO	NO	8,14	NO	NO	NO
VAGINAL BIRTH	YES	NO	FEMALE	9	9	3760	NO	NO	NO	NO	YES	NO	NO	NO	NO	NO	8,14	NO	YES	NO
VAGINAL BIRTH	YES	NO	FEMALE	9	10	2675	NO	YES	NO	NO	YES	NO	NO	NO	NO	NO	8,14	NO	NO	NO
VAGINAL BIRTH	YES	NO	FEMALE	8	9	2600	NO	NO	NO	NO	YES	NO	NO	NO	NO	NO	8,14	NO	YES	NO
VAGINAL BIRTH	NO	NO	FEMALE	8	9	2940	NO	NO	NO	NO	YES	NO	NO	NO	NO	NO	8,14	NO	NO	NO
C-SECTIONNO	NO	FEMALE	9	10	4010	NO	NO	NO	NO	YES	NO	NO	NO	NO	NO	8,14	NO	NO	NO	
VAGINAL BIRTH	NO	YES	MALE	8	9	3190	NO	NO	NO	NO	YES	NO	NO	NO	NO	NO	8,14	NO	YES	YES
VAGINAL BIRTH	YES	NO	MALE	6	8	1830	YES	NO	NO	NO	YES	NO	NO	NO	NO	YES	8,14	NO	NO	YES
VAGINAL BIRTH	NO	NO	FEMALE	9	10	3410	NO	NO	NO	NO	YES	NO	NO	NO	NO	NO	8,14	NO	NO	YES
VAGINAL BIRTH	NO	NO	FEMALE	9	10	2830	NO	NO	NO	NO	YES	NO	NO	NO	NO	NO	8,14	NO	YES	YES
VAGINAL BIRTH	YES	YES	MALE	9	9	3085	NO	NO	NO	NO	YES	NO	NO	NO	NO	NO	8,14	NO	YES	YES
VAGINAL BIRTH	NO	NO	MALE	9	9	3050	NO	NO	NO	NO	YES	NO	NO	NO	NO	NO	8,14	NO	NO	YES
VAGINAL BIRTH	NO	NO	MALE	9	10	2890	NO	NO	NO	NO	YES	NO	NO	NO	NO	NO	8,14	NO	NO	YES
VAGINAL BIRTH	NO	NO	MALE	2	7	3265	NO	NO	NO	NO	YES	NO	NO	NO	NO	YES	8,14	NO	YES	YES
VAGINAL BIRTH	NO	NO	MALE	5	8	3390	YES	NO	NO	NO	YES	NO	NO	NO	NO	YES	8,14	NO	YES	YES
VAGINAL BIRTH	YES	NO	MALE	9	9	3035	NO	NO	YES	NO	YES	NO	YES	NO	NO	NO	8,29	NO	YES	NO
VAGINAL BIRTH	YES	YES	MALE	9	9	3600	NO	NO	YES	NO	YES	NO	YES	NO	NO	NO	8,29	NO	YES	NO
VAGINAL BIRTH	NO	NO	MALE	9	10	3075	NO	NO	NO	NO	YES	NO	NO	NO	NO	NO	8,29	NO	YES	NO
VAGINAL BIRTH	YES	NO	MALE	9	9	2745	NO	YES	NO	NO	YES	NO	NO	NO	NO	NO	8,29	NO	NO	NO
VAGINAL BIRTH	YES	YES	FEMALE	8	10	2720	NO	NO	NO	NO	YES	NO	NO	NO	NO	NO	8,29	NO	YES	NO
VAGINAL BIRTH	YES	NO	MALE	10	10	2735	NO	YES	NO	NO	YES	NO	NO	NO	NO	NO	8,29	NO	NO	NO
VAGINAL BIRTH	NO	NO	MALE	9	10	3310	NO	NO	NO	NO	YES	NO	NO	NO	NO	NO	8,29	NO	YES	NO
C-SECTIONYES	NO	FEMALE	5	8	2210	YES	NO	NO	NO	YES	NO	NO	NO	NO	YES	8,29	NO	NO	NO	
C-SECTIONYES	NO	FEMALE	9	10	3135	NO	NO	NO	NO	YES	NO	NO	NO	YES	NO	8,29	NO	NO	NO	
C-SECTIONNO	NO	FEMALE	9	9	3040	NO	NO	NO	NO	YES	NO	NO	NO	NO	NO	8,29	NO	NO	NO	
C-SECTIONNO	NO	MALE	9	10	3185	NO	NO	NO	NO	YES	NO	NO	NO	NO	NO	8,29	NO	NO	NO	
C-SECTIONYES	NO	MALE	9	10	3890	YES	NO	YES	NO	YES	NO	NO	NO	NO	NO	8,29	NO	NO	NO	
C-SECTIONNO	NO	FEMALE	8	9	3340	NO	NO	NO	NO	YES	NO	NO	NO	NO	NO	8,29	NO	NO	NO	
VAGINAL BIRTH	NO	NO	MALE	9	9	2850	NO	NO	NO	NO	YES	NO	NO	NO	NO	NO	8,29	NO	YES	YES
VAGINAL BIRTH	YES	YES	MALE	9	10	2440	NO	YES	YES	NO	YES	NO	NO	NO	NO	NO	8,29	NO	NO	YES
VAGINAL BIRTH	YES	NO	FEMALE	9	10	2105	YES	NO	NO	NO	YES	NO	NO	NO	NO	NO	8,29	NO	NO	YES
VAGINAL BIRTH	YES	NO	MALE	8	9	2790	YES	NO	NO	NO	YES	NO	NO	NO	NO	NO	8,29	NO	NO	YES
VAGINAL BIRTH	NO	NO	MALE	9	10	2755	NO	NO	NO	NO	YES	NO	NO	NO	NO	NO	8,29	NO	NO	YES
C-SECTIONNO	NO	MALE	8	9	3635	NO	NO	NO	NO	YES	NO	NO	NO	NO	NO	8,29	NO	YES	YES	
VAGINAL BIRTH	YES	NO	FEMALE	9	9	2280	NO	NO	NO	NO	YES	NO	NO	NO	NO	NO	8,43	NO	NO	NO

VAGINAL BIRTH	NO	YES	FEMALE	8	9	2200	YES	NO	NO	NO	YES	NO	NO	NO	NO	NO	8,43	NO	NO	NO
VAGINAL BIRTH	YES	NO	FEMALE	8	8	3420	NO	YES	YES	NO	YES	NO	NO	NO	NO	NO	8,43	NO	NO	NO
VAGINAL BIRTH	NO	NO	FEMALE	9	10	2870	NO	NO	NO	NO	YES	NO	NO	NO	NO	NO	8,43	NO	NO	NO
VAGINAL BIRTH	YES	YES	MALE	9	9	3385	NO	YES	NO	NO	YES	NO	NO	NO	NO	NO	8,43	NO	YES	NO
VAGINAL BIRTH	YES	NO	FEMALE	8	9	3180	NO	YES	NO	NO	YES	NO	NO	NO	NO	NO	8,43	NO	NO	NO
C-SECTION	YES	NO	FEMALE	8	9	2345	NO	NO	NO	NO	YES	NO	NO	NO	NO	NO	8,43	NO	NO	NO
C-SECTION	NO	NO	MALE	10	10	3715	NO	NO	NO	NO	YES	NO	NO	NO	NO	NO	8,43	NO	NO	NO
C-SECTION	YES	NO	MALE	9	10	3390	NO	NO	NO	NO	YES	NO	NO	NO	NO	NO	8,43	NO	NO	NO
C-SECTION	YES	NO	FEMALE	8	9	2385	NO	NO	NO	NO	YES	NO	NO	NO	YES	NO	8,43	NO	NO	NO
VAGINAL BIRTH	YES	NO	MALE	8	9	3605	NO	NO	NO	NO	YES	NO	NO	NO	NO	NO	8,43	NO	NO	YES
VAGINAL BIRTH	YES	NO	MALE	9	9	2685	NO	YES	YES	NO	YES	NO	NO	NO	NO	NO	8,43	NO	NO	YES
VAGINAL BIRTH	YES	NO	MALE	9	10	3110	NO	NO	NO	NO	YES	NO	NO	NO	NO	NO	8,43	NO	YES	YES
VAGINAL BIRTH	NO	NO	FEMALE	8	9	4030	NO	NO	NO	NO	YES	NO	NO	NO	NO	NO	8,43	NO	NO	YES
VAGINAL BIRTH	NO	NO	MALE	10	10	3410	NO	NO	NO	NO	YES	NO	NO	NO	NO	NO	8,43	NO	NO	YES
VAGINAL BIRTH	NO	NO	FEMALE	8	9	2760	NO	NO	NO	NO	YES	NO	NO	NO	NO	NO	8,43	NO	NO	YES
VAGINAL BIRTH	YES	NO	FEMALE	8	9	1280	YES	YES	NO	YES	YES	NO	NO	NO	NO	NO	8,43	NO	NO	YES
VAGINAL BIRTH	YES	NO	FEMALE	7	9	1395	YES	YES	NO	YES	YES	NO	NO	NO	NO	NO	8,43	NO	NO	YES
VAGINAL BIRTH	YES	NO	MALE	0	0	495		NO	NO	NO	YES	NO	NO	YES	NO	YES	8,43	NO	NO	YES
VAGINAL BIRTH	YES	NO	MALE	6	8	4485	NO	YES	NO	NO	YES	NO	NO	NO	NO	YES	8,57	NO	YES	NO
C-SECTION	YES	NO	MALE	6	9	3740	YES	NO	YES	NO	YES	NO	NO	NO	NO	YES	8,57	NO	NO	NO
VAGINAL BIRTH	YES	YES	FEMALE	9	10	2885	NO	NO	NO	NO	YES	NO	NO	NO	NO	NO	8,57	NO	NO	NO
C-SECTION	YES	NO	MALE	8	9	3550	NO	NO	YES	NO	YES	NO	NO	NO	NO	NO	8,57	NO	NO	NO
C-SECTION	NO	NO	FEMALE	8	10	2930	NO	NO	NO	NO	YES	NO	NO	NO	NO	NO	8,57	NO	YES	NO
C-SECTION	YES	NO	MALE	9	9	3930	NO	NO	YES	NO	YES	NO	NO	NO	NO	NO	8,57	NO	NO	NO
C-SECTION	YES	NO	MALE	4	9	2880	YES	YES	YES	NO	YES	NO	NO	NO	NO	YES	8,57	NO	NO	NO
VAGINAL BIRTH	YES	NO	MALE	9	9	2090	YES	NO	NO	NO	YES	NO	NO	NO	NO	NO	8,57	NO	YES	YES
VAGINAL BIRTH	YES	NO	FEMALE	10	10	3145	NO	NO	NO	NO	YES	NO	NO	NO	NO	NO	8,57	NO	YES	YES
VAGINAL BIRTH	YES	NO	MALE	9	10	3135	NO	NO	NO	NO	YES	NO	NO	NO	NO	NO	8,57	NO	YES	YES
VAGINAL BIRTH	NO	NO	MALE	9	10	2840	NO	NO	NO	NO	YES	NO	NO	NO	NO	NO	8,57	NO	NO	YES
VAGINAL BIRTH	NO	NO	FEMALE	10	10	3195	NO	NO	NO	NO	YES	NO	NO	NO	NO	NO	8,57	NO	NO	YES
VAGINAL BIRTH	NO	NO	FEMALE	8	8	3200	NO	NO	NO	NO	YES	NO	NO	NO	NO	NO	8,57	NO	NO	YES
VAGINAL BIRTH	NO	NO	MALE	9	10	3230	NO	NO	NO	NO	YES	NO	NO	NO	NO	NO	8,57	NO	NO	YES
VAGINAL BIRTH	NO	YES	FEMALE	8	9	2975	NO	NO	NO	NO	YES	NO	NO	NO	NO	NO	8,57	NO	YES	YES
VAGINAL BIRTH	NO	NO	FEMALE	9	9	2780	NO	NO	NO	NO	YES	NO	NO	NO	NO	NO	8,57	NO	NO	YES
VAGINAL BIRTH	NO	NO	FEMALE	9	10	3250	NO	NO	NO	NO	YES	NO	NO	NO	NO	NO	8,57	NO	NO	YES
VAGINAL BIRTH	NO	NO	MALE	9	10	3035	NO	NO	NO	NO	YES	NO	NO	NO	NO	NO	8,57	NO	NO	YES
VAGINAL BIRTH	NO	NO	FEMALE	9	10	3045	NO	NO	NO	NO	YES	NO	NO	NO	NO	NO	8,57	NO	YES	YES
VAGINAL BIRTH	YES	YES	FEMALE	8	9	2735	NO	NO	YES	NO	YES	NO	NO	NO	NO	NO	8,71	NO	YES	NO
VAGINAL BIRTH	NO	NO	MALE	7	8	3480	NO	NO	NO	NO	YES	NO	NO	NO	NO	NO	8,71	NO	YES	NO
C-SECTION	NO	NO	FEMALE	8	9	3260	NO	NO	NO	NO	YES	NO	NO	NO	NO	NO	8,71	NO	NO	NO
VAGINAL BIRTH	NO	NO	MALE	7	9	4030	NO	NO	NO	NO	YES	NO	NO	NO	NO	NO	8,71	NO	YES	NO
VAGINAL BIRTH	YES	NO	MALE	8	9	3785	NO	NO	NO	NO	YES	NO	NO	NO	NO	NO	8,71	NO	NO	NO
VAGINAL BIRTH	YES	NO	MALE	8	9	2300	NO	NO	NO	NO	YES	NO	NO	NO	NO	NO	8,71	NO	NO	NO
C-SECTION	NO	NO	FEMALE	8	9	3095	NO	NO	NO	NO	YES	NO	NO	NO	NO	NO	8,71	NO	NO	NO
C-SECTION	NO	NO	MALE	8	9	2605	NO	NO	NO	NO	YES	NO	NO	NO	NO	NO	8,71	NO	NO	NO
VAGINAL BIRTH	NO	NO	FEMALE	9	10	3340	NO	NO	NO	NO	YES	NO	NO	NO	NO	NO	8,71	NO	YES	NO
C-SECTION	YES	NO	FEMALE	9	10	2775	YES	NO	NO	NO	YES	NO	NO	NO	NO	NO	8,71	NO	NO	NO
C-SECTION	YES	NO	MALE	10	10	2795	NO	NO	NO	NO	YES	NO	NO	NO	NO	NO	8,71	NO	NO	NO
VAGINAL BIRTH	NO	NO	FEMALE	9	10	3420	NO	NO	NO	NO	YES	NO	NO	NO	NO	NO	8,71	NO	NO	NO
C-SECTION	NO	NO	FEMALE	9	10	3020	NO	NO	NO	NO	YES	NO	NO	NO	NO	NO	8,71	NO	NO	NO
C-SECTION	YES	NO	MALE	9	9	2460	NO	NO	NO	NO	YES	NO	NO	NO	NO	NO	8,71	NO	NO	YES
VAGINAL BIRTH	NO	NO	MALE	9	9	3640	NO	NO	NO	NO	YES	NO	NO	NO	NO	NO	8,71	NO	YES	YES
VAGINAL BIRTH	NO	NO	FEMALE	9	9	3210	NO	NO	NO	NO	YES	NO	NO	NO	NO	NO	8,71	NO	NO	YES

VAGINAL BIRTH		NO	NO	FEMALE	9	10	2850	NO	NO	NO	NO	YES	NO	NO	NO	NO	NO	8,71	NO	NO	YES
VAGINAL BIRTH		NO	NO	MALE	8	9	3280	NO	NO	NO	NO	YES	NO	NO	NO	NO	NO	8,71	NO	NO	YES
VAGINAL BIRTH		NO	NO	MALE	9	10	3585	NO	NO	NO	NO	YES	NO	NO	NO	NO	NO	8,71	NO	YES	YES
VAGINAL BIRTH		NO	NO	MALE	8	10	2950	NO	NO	NO	NO	YES	NO	NO	NO	NO	NO	8,71	NO	NO	YES
C-SECTION	NO	NO	MALE	8	9	2765	NO	NO	NO	NO	YES	NO	NO	NO	NO	NO	8,71	NO	NO	YES	
C-SECTION	YES	NO	FEMALE	9	9	3500	NO	YES	NO	NO	YES	NO	NO	NO	NO	NO	8,86	NO	YES	NO	
VAGINAL BIRTH		YES	NO	FEMALE	8	9	2885	NO	YES	YES	NO	YES	NO	NO	NO	NO	NO	8,86	NO	NO	NO
VAGINAL BIRTH		YES	NO	MALE	9	9	3395	NO	YES	YES	NO	YES	NO	NO	NO	NO	NO	8,86	NO	NO	NO
VAGINAL BIRTH		YES	NO	MALE	9	10	2755	NO	NO	NO	NO	YES	NO	NO	NO	NO	NO	8,86	NO	YES	NO
C-SECTION	YES	NO	MALE	8	8	3960	YES	NO	NO	NO	YES	NO	NO	NO	NO	NO	8,86	NO	NO	NO	
VAGINAL BIRTH		YES	NO	MALE	9	9	2945	NO	NO	NO	NO	YES	NO	NO	NO	YES	NO	8,86	NO	NO	YES
VAGINAL BIRTH		NO	NO	FEMALE	10	10	3950	NO	NO	NO	NO	YES	NO	NO	NO	NO	NO	8,86	NO	NO	YES
VAGINAL BIRTH		NO	NO	MALE	9	9	3750	NO	NO	NO	NO	YES	NO	NO	NO	NO	NO	8,86	NO	YES	YES
VAGINAL BIRTH		NO	NO	MALE	9	10	3325	NO	NO	NO	NO	YES	NO	NO	NO	NO	NO	8,86	NO	NO	YES
VAGINAL BIRTH		YES	NO	MALE	8	9	3195	YES	NO	NO	NO	YES	NO	NO	NO	YES	NO	8,86	NO	YES	YES
VAGINAL BIRTH		NO	NO	MALE	8	9	2930	NO	NO	NO	NO	YES	NO	NO	NO	NO	NO	8,86	NO	NO	YES
VAGINAL BIRTH		YES	YES	FEMALE	7	9	3540	NO	NO	NO	NO	YES	NO	NO	NO	NO	NO	8,86	NO	YES	YES
VAGINAL BIRTH		YES	NO	FEMALE	8	8	3025	NO	NO	YES	NO	YES	NO	NO	NO	NO	NO	8,86	NO	NO	YES
VAGINAL BIRTH		NO	NO	FEMALE	9	10	2525	NO	NO	NO	NO	YES	NO	NO	NO	NO	NO	8,86	NO	NO	YES
VAGINAL BIRTH		NO	NO	MALE	8	9	4160	NO	NO	NO	NO	YES	NO	NO	NO	NO	NO	8,86	NO	YES	YES
VAGINAL BIRTH		YES	NO	FEMALE	8	9	2995	NO	NO	YES	NO	YES	NO	NO	NO	NO	NO	8,86	NO	NO	YES
VAGINAL BIRTH		YES	NO	MALE	8	10	975	YES	NO	NO	NO	YES	NO	NO	NO	NO	NO	8,86	NO	YES	YES
VAGINAL BIRTH		YES	NO	FEMALE	7	9	2455	NO	NO	NO	NO	YES	NO	NO	NO	NO	NO	9,00	NO	NO	NO
C-SECTION	NO	NO	MALE	9	9	3915	NO	NO	NO	NO	YES	NO	NO	NO	NO	NO	9,00	NO	NO	NO	
VAGINAL BIRTH		NO	NO	MALE	9	9	3060	NO	NO	NO	NO	YES	NO	NO	NO	NO	NO	9,00	NO	NO	NO
VAGINAL BIRTH		YES	NO	MALE	0	0	2475		YES	NO	NO	YES	NO	NO	YES	NO	NO	9,00	NO	NO	NO
C-SECTION	YES	NO	MALE	9	9	2980	NO	NO	NO	NO	YES	NO	NO	NO	NO	NO	9,00	NO	NO	NO	
VAGINAL BIRTH		YES	NO	FEMALE	8	9	3535	NO	NO	YES	NO	YES	NO	NO	NO	NO	NO	9,00	NO	YES	NO
C-SECTION	YES	NO	MALE	5	8	2665	NO	YES	YES	NO	YES	NO	NO	NO	NO	YES	9,00	NO	NO	NO	
C-SECTION	YES	NO	MALE	8	9	2580	NO	YES	NO	NO	YES	NO	NO	NO	NO	NO	9,00	NO	NO	NO	
VAGINAL BIRTH		YES	NO	MALE	3	8	3810	NO	NO	YES	NO	YES	NO	NO	NO	NO	YES	9,00	NO	NO	NO
VAGINAL BIRTH		NO	NO	MALE	8	9	3330	NO	NO	NO	NO	YES	NO	NO	NO	NO	NO	9,00	NO	NO	NO
VAGINAL BIRTH		NO	NO	FEMALE	9	10	2650	NO	NO	NO	NO	YES	NO	NO	NO	NO	NO	9,00	NO	NO	NO
C-SECTION	YES	NO	MALE	8	9	1305	YES	YES	NO	NO	YES	YES	NO	NO	NO	NO	9,00	NO	NO	NO	
C-SECTION	YES	NO	FEMALE	9	9	2645	YES	NO	NO	NO	YES	NO	NO	NO	NO	NO	9,00	NO	NO	NO	
C-SECTION	YES	NO	MALE	8	10	1860	YES	NO	NO	NO	YES	NO	NO	NO	NO	NO	9,00	NO	NO	NO	
C-SECTION	YES	NO	MALE	8	9	3070	NO	YES	NO	NO	YES	NO	NO	NO	NO	NO	9,00	NO	NO	NO	
VAGINAL BIRTH		NO	NO	FEMALE	9	10	2705	NO	NO	NO	NO	YES	NO	NO	NO	NO	NO	9,00	NO	YES	YES
VAGINAL BIRTH		NO	NO	FEMALE	9	9	3015	NO	NO	NO	NO	YES	NO	NO	NO	NO	NO	9,00	NO	YES	YES
VAGINAL BIRTH		YES	NO	FEMALE	8	9	2490	NO	NO	NO	NO	YES	NO	NO	NO	NO	NO	9,00	NO	NO	YES
VAGINAL BIRTH		YES	NO	FEMALE	8	9	3080	NO	NO	YES	NO	YES	NO	NO	NO	NO	NO	9,00	NO	YES	YES
VAGINAL BIRTH		YES	NO	MALE	5	8	3060	NO	NO	NO	NO	YES	NO	NO	NO	YES	YES	9,00	NO	YES	YES
VAGINAL BIRTH		YES	NO	FEMALE	8	9	2975	NO	NO	NO	NO	YES	NO	NO	NO	NO	NO	9,00	NO	YES	YES
VAGINAL BIRTH		YES	NO	FEMALE	9	10	2810	NO	NO	YES	NO	YES	NO	NO	NO	NO	NO	9,00	NO	YES	YES
VAGINAL BIRTH		NO	NO	FEMALE	9	10	2680	NO	NO	NO	NO	YES	NO	NO	NO	NO	NO	9,00	NO	NO	YES
VAGINAL BIRTH		YES	NO	MALE	8	9	3810	NO	NO	NO	NO	YES	NO	NO	NO	NO	NO	9,00	NO	NO	YES
VAGINAL BIRTH		NO	NO	MALE	10	10	2915	NO	NO	NO	NO	YES	NO	NO	NO	NO	NO	9,00	NO	YES	YES
VAGINAL BIRTH		NO	NO	MALE	8	9	2630	NO	NO	NO	NO	YES	NO	NO	NO	NO	NO	9,14	NO	NO	NO
VAGINAL BIRTH		YES	NO	FEMALE	9	10	2955	NO	NO	YES	NO	YES	NO	NO	NO	NO	NO	9,14	NO	NO	NO
VAGINAL BIRTH		YES	YES	FEMALE	8	9	2755	NO	NO	NO	NO	YES	NO	NO	NO	NO	NO	9,14	NO	YES	NO
C-SECTION	NO	NO	FEMALE	8	9	3620	NO	NO	NO	NO	YES	NO	NO	NO	NO	NO	9,14	NO	YES	NO	
VAGINAL BIRTH		NO	NO	MALE	8	9	3185	NO	NO	NO	NO	YES	NO	NO	NO	NO	NO	9,14	NO	YES	NO
C-SECTION	YES	NO	MALE	6	9	1705	YES	NO	NO	NO	YES	NO	NO	NO	NO	YES	9,14	NO	NO	NO	

C-SECTION	YES	NO	FEMALE	9	10	3925	NO	NO	YES	NO	YES	NO	NO	NO	NO	NO	9,14	NO	NO	NO	
C-SECTION	YES	NO	FEMALE	2	7	830	YES	YES	NO	NO	YES	NO	NO	NO	NO	YES	9,14	NO	NO	NO	
C-SECTION	NO	NO	FEMALE	9	10	3585	NO	NO	NO	NO	YES	NO	NO	NO	NO	NO	9,14	NO	NO	NO	
C-SECTION	NO	NO	MALE	9	10	3340	NO	NO	NO	NO	YES	NO	NO	NO	NO	NO	9,14	NO	NO	NO	
C-SECTION	YES	NO	FEMALE	7	9	2305	NO	NO	NO	NO	YES	NO	NO	NO	NO	NO	9,14	NO	NO	NO	
C-SECTION	NO	NO	MALE	2	7	2435	YES	NO	NO	NO	YES	NO	NO	NO	NO	YES	9,14	NO	NO	NO	
VAGINAL BIRTH		NO	NO	MALE	9	10	3210	NO	NO	NO	NO	YES	NO	NO	NO	NO	NO	9,14	NO	NO	YES
C-SECTION	NO	NO	MALE	8	9	3210	NO	NO	NO	NO	YES	NO	NO	NO	NO	NO	9,14	NO	NO	YES	
VAGINAL BIRTH		NO	YES	MALE	9	9	3710	NO	NO	NO	NO	YES	NO	NO	NO	NO	NO	9,14	NO	YES	YES
VAGINAL BIRTH		NO	NO	MALE	9	10	2835	NO	NO	NO	NO	YES	NO	NO	NO	NO	NO	9,14	NO	NO	YES
C-SECTION	YES	NO	MALE	8	9	3420	NO	NO	NO	NO	YES	NO	NO	NO	NO	NO	9,14	NO	NO	YES	
C-SECTION	NO	NO	MALE	9	10	3630	NO	NO	NO	NO	YES	NO	NO	NO	NO	NO	9,14	NO	NO	YES	
VAGINAL BIRTH		YES	NO	FEMALE	8	8	3520	NO	NO	NO	NO	YES	NO	NO	NO	NO	NO	9,14	NO	NO	YES
VAGINAL BIRTH		YES	NO	MALE	8	9	2470	YES	YES	NO	NO	YES	NO	NO	NO	NO	NO	9,29	NO	NO	NO
VAGINAL BIRTH		NO	NO	FEMALE	8	9	3270	NO	NO	NO	NO	YES	NO	NO	NO	NO	NO	9,29	NO	YES	NO
VAGINAL BIRTH		NO	NO	MALE	0	0			NO	NO	NO	YES	NO	NO	NO	NO	NO	9,29	NO	NO	NO
C-SECTION	NO	NO	MALE	8	9	3740	NO	NO	NO	NO	YES	NO	NO	NO	NO	NO	9,29	NO	YES	NO	
VAGINAL BIRTH		NO	NO	FEMALE	9	10	2980	NO	NO	NO	NO	YES	NO	NO	NO	NO	NO	9,29	NO	NO	NO
C-SECTION	YES	NO	FEMALE	8	9	1920	NO	NO	NO	NO	YES	NO	NO	NO	YES	NO	9,29	NO	NO	NO	
C-SECTION	YES	NO	FEMALE	8	9	1085	NO	YES	NO	NO	YES	NO	NO	NO	NO	NO	9,29	NO	NO	NO	
C-SECTION	NO	NO	MALE	8	9	3025	NO	NO	NO	NO	YES	NO	NO	NO	NO	NO	9,29	NO	NO	NO	
C-SECTION	YES	NO	FEMALE	9	10	3505	NO	YES	YES	NO	YES	NO	NO	NO	NO	NO	9,29	NO	NO	NO	
C-SECTION	YES	NO	MALE	9	9	3240	NO	YES	NO	NO	YES	NO	NO	NO	NO	NO	9,29	NO	NO	NO	
C-SECTION	NO	NO	MALE	9	9	3495	NO	NO	NO	NO	YES	NO	NO	NO	NO	NO	9,29	NO	NO	NO	
VAGINAL BIRTH		NO	NO	FEMALE	8	9	2540	NO	NO	NO	NO	YES	NO	NO	NO	NO	NO	9,29	NO	YES	YES
VAGINAL BIRTH		NO	NO	MALE	9	10	3210	NO	NO	NO	NO	YES	NO	NO	NO	NO	NO	9,29	NO	NO	YES
VAGINAL BIRTH		YES	NO	FEMALE	10	10	2500	NO	NO	NO	NO	YES	NO	NO	NO	NO	NO	9,29	NO	NO	YES
C-SECTION	NO	NO	MALE	9	9	3045	NO	NO	NO	NO	YES	NO	NO	NO	NO	NO	9,29	NO	NO	YES	
VAGINAL BIRTH		NO	NO	FEMALE	8	9	2775	NO	NO	NO	NO	YES	NO	NO	NO	NO	NO	9,29	NO	NO	YES
VAGINAL BIRTH		NO	NO	FEMALE	9	9	2930	NO	NO	NO	NO	YES	NO	NO	NO	NO	NO	9,29	NO	NO	YES
VAGINAL BIRTH		YES	NO	FEMALE	9	9	3080	NO	NO	NO	NO	YES	NO	NO	NO	NO	NO	9,29	NO	NO	YES
VAGINAL BIRTH		NO	NO	FEMALE	9	9	3165	NO	NO	NO	NO	YES	NO	NO	NO	NO	NO	9,29	NO	YES	YES
VAGINAL BIRTH		NO	NO	MALE	8	9	3185	NO	NO	NO	NO	YES	NO	NO	NO	NO	NO	9,43	NO	NO	NO
VAGINAL BIRTH		NO	YES	FEMALE	8	9	2825	NO	NO	NO	NO	YES	NO	NO	NO	NO	NO	9,43	NO	NO	NO
VAGINAL BIRTH		YES	NO	FEMALE	9	9	3175	NO	NO	NO	NO	YES	NO	NO	NO	NO	NO	9,43	NO	YES	NO
VAGINAL BIRTH		YES	NO	FEMALE	8	9	3495	NO	NO	NO	NO	YES	NO	NO	NO	NO	NO	9,43	NO	YES	NO
VAGINAL BIRTH		YES	NO	FEMALE	8	9	3315	NO	YES	NO	NO	YES	NO	NO	NO	NO	NO	9,43	NO	NO	NO
VAGINAL BIRTH		NO	NO	MALE	8	10	3060	NO	NO	NO	NO	YES	NO	NO	NO	NO	NO	9,43	NO	NO	NO
VAGINAL BIRTH		NO	NO	FEMALE	7	9	3290	NO	NO	NO	NO	YES	NO	NO	NO	NO	NO	9,43	NO	YES	NO
VAGINAL BIRTH		YES	YES	MALE	0	0	3020		YES	YES	NO	YES	NO	NO	YES	NO	YES	9,43	NO	NO	NO
VAGINAL BIRTH		YES	NO	MALE	9	10	3230	NO	YES	NO	NO	YES	NO	NO	NO	NO	NO	9,43	NO	NO	NO
VAGINAL BIRTH		YES	NO	MALE	0	0	210		NO	NO	NO	YES	NO	NO	YES	NO	NO	9,43	NO	NO	NO
VAGINAL BIRTH		YES	NO	MALE	8	9	2555	NO	NO	NO	NO	YES	NO	NO	NO	NO	NO	9,43	NO	NO	NO
C-SECTION	YES	NO	FEMALE	9	10	3370	NO	YES	YES	NO	YES	NO	NO	NO	NO	NO	9,43	NO	NO	NO	
C-SECTION	NO	NO	FEMALE	8	9	2980	NO	NO	NO	NO	YES	NO	NO	NO	NO	NO	9,43	NO	NO	NO	
C-SECTION	NO	NO	MALE	9	9	3485	NO	NO	NO	NO	YES	NO	NO	NO	NO	NO	9,43	NO	NO	NO	
C-SECTION	YES	NO	MALE	9	10	2195	YES	YES	NO	YES	YES	NO	NO	NO	NO	NO	9,43	NO	NO	NO	
C-SECTION	YES	NO	FEMALE	8	9	1890	YES	YES	NO	YES	YES	NO	NO	NO	NO	NO	9,43	NO	NO	NO	
C-SECTION	YES	NO	FEMALE	7	9	3345	NO	NO	NO	NO	YES	NO	NO	NO	YES	NO	9,43	NO	NO	NO	
C-SECTION	YES	NO	FEMALE	8	10	4345	NO	YES	YES	NO	YES	NO	NO	NO	NO	NO	9,43	NO	NO	NO	
C-SECTION	NO	NO	MALE	8	9	3590	NO	NO	NO	NO	YES	NO	NO	NO	NO	NO	9,43	NO	NO	NO	
VAGINAL BIRTH		YES	NO	MALE	9	10	3140	NO	NO	NO	NO	YES	NO	NO	NO	NO	NO	9,43	NO	NO	YES
VAGINAL BIRTH		NO	NO	FEMALE	8	9	3550	NO	NO	NO	NO	YES	NO	NO	NO	NO	NO	9,43	NO	NO	YES

C-SECTIONYES	NO	MALE	8	9	3005	NO	NO	NO	NO	YES	NO	NO	NO	NO	NO	9,43	NO	NO	YES	
VAGINAL BIRTH	NO	NO	FEMALE	8	9	4040	NO	NO	NO	NO	YES	NO	NO	NO	NO	NO	9,43	NO	NO	YES
VAGINAL BIRTH	NO	NO	FEMALE	8	9	3000	NO	NO	NO	NO	YES	NO	NO	NO	NO	NO	9,43	NO	YES	YES
VAGINAL BIRTH	NO	NO	MALE	9	9	2575	NO	NO	NO	NO	YES	NO	NO	NO	NO	NO	9,43	NO	NO	YES
VAGINAL BIRTH	NO	NO	FEMALE	8	9	2180	NO	NO	NO	NO	YES	NO	NO	NO	NO	NO	9,57	NO	NO	NO
C-SECTIONNO	NO	FEMALE	9	9	3270	NO	NO	NO	NO	YES	NO	NO	NO	NO	NO	9,57	NO	YES	NO	
VAGINAL BIRTH	YES	NO	FEMALE	9	10	1850	NO	NO	YES	NO	YES	NO	NO	NO	NO	NO	9,57	NO	NO	NO
VAGINAL BIRTH	NO	NO	FEMALE	9	10	2850	NO	NO	NO	NO	YES	NO	NO	NO	NO	NO	9,57	NO	NO	NO
VAGINAL BIRTH	YES	NO	FEMALE	8	9	2745	NO	NO	YES	NO	YES	NO	NO	NO	NO	NO	9,57	NO	YES	NO
VAGINAL BIRTH	YES	NO	MALE	9	10	3430	NO	YES	NO	NO	YES	NO	NO	NO	NO	NO	9,57	NO	NO	NO
C-SECTIONYES	NO	MALE	8	7	3285	YES	YES	NO	NO	YES	NO	YES	NO	NO	NO	9,57	NO	NO	NO	
VAGINAL BIRTH	YES	NO	MALE	7	9	3930	NO	NO	NO	NO	YES	NO	NO	NO	NO	NO	9,57	NO	YES	NO
C-SECTIONYES	NO	FEMALE	8	9	2045	NO	NO	NO	NO	YES	NO	NO	NO	NO	NO	9,57	NO	NO	NO	
C-SECTIONYES	NO	MALE	9	9	2165	NO	NO	YES	NO	YES	NO	NO	NO	NO	NO	9,57	NO	NO	NO	
C-SECTIONYES	NO	FEMALE	9	9	2815	NO	NO	YES	NO	YES	NO	NO	NO	NO	NO	9,57	NO	NO	NO	
C-SECTIONYES	NO	MALE	8	9	3040	NO	YES	NO	NO	YES	NO	NO	NO	NO	NO	9,57	NO	NO	NO	
C-SECTIONYES	NO	MALE	8	9	4165	NO	NO	YES	NO	YES	NO	NO	NO	NO	NO	9,57	NO	NO	NO	
C-SECTIONYES	NO	FEMALE	8	10	3305	NO	YES	NO	NO	YES	NO	NO	NO	NO	NO	9,57	NO	NO	NO	
C-SECTIONYES	NO	FEMALE	7	10	720	YES	YES	NO	NO	YES	NO	NO	NO	NO	NO	9,57	NO	NO	NO	
VAGINAL BIRTH	NO	NO	FEMALE	6	9	3400	NO	NO	NO	NO	YES	NO	NO	NO	NO	YES	9,57	NO	NO	YES
VAGINAL BIRTH	NO	NO	FEMALE	9	9	3975	NO	NO	NO	NO	YES	NO	NO	NO	NO	NO	9,57	NO	YES	YES
VAGINAL BIRTH	NO	NO	FEMALE	9	10	3840	NO	NO	NO	NO	YES	NO	NO	NO	NO	NO	9,57	NO	NO	YES
VAGINAL BIRTH	NO	NO	MALE	6	7	2890	YES	NO	NO	NO	YES	NO	NO	NO	NO	YES	9,57	NO	YES	YES
VAGINAL BIRTH	YES	NO	UNDETERMINATED		1	0	515		NO	NO	NO	YES	NO	NO	YES	NO	NO	9,57	NO	NO
YES																				
VAGINAL BIRTH	NO	NO	FEMALE	9	10	3605	NO	NO	NO	NO	YES	NO	NO	NO	NO	NO	9,57	NO	NO	YES
VAGINAL BIRTH	NO	YES	FEMALE	7	8	3375	NO	NO	NO	NO	YES	NO	NO	NO	NO	NO	9,57	NO	NO	YES
VAGINAL BIRTH	NO	NO	MALE	9	9	3095	NO	NO	NO	NO	YES	NO	NO	NO	NO	NO	9,57	NO	NO	YES
VAGINAL BIRTH	YES	NO	MALE	7	9	2660	NO	YES	NO	NO	YES	NO	NO	NO	NO	NO	9,71	NO	NO	NO
C-SECTIONYES	NO	FEMALE	9	10	2110	NO	NO	NO	NO	YES	NO	NO	NO	NO	NO	9,71	NO	NO	NO	
C-SECTIONNO	NO	FEMALE	9	9	3210	NO	NO	NO	NO	YES	NO	NO	NO	NO	NO	9,71	NO	YES	NO	
VAGINAL BIRTH	NO	NO	FEMALE	9	10	2915	NO	NO	NO	NO	YES	NO	NO	NO	NO	NO	9,71	NO	YES	NO
VAGINAL BIRTH	YES	NO	FEMALE	8	9	3290	NO	NO	NO	NO	YES	NO	NO	NO	NO	NO	9,71	NO	YES	NO
C-SECTIONNO	NO	FEMALE	8	9	2965	NO	NO	NO	NO	YES	NO	NO	NO	NO	NO	9,71	NO	NO	NO	
C-SECTIONYES	NO	FEMALE	8	9	2120	NO	YES	YES	NO	YES	NO	NO	NO	NO	NO	9,71	NO	NO	NO	
C-SECTIONNO	NO	FEMALE	9	10	4135	NO	NO	NO	NO	YES	NO	NO	NO	NO	NO	9,71	NO	YES	YES	
VAGINAL BIRTH	YES	NO	FEMALE	1	1	420	NO	NO	NO	NO	YES	NO	NO	YES	NO	NO	9,71	NO	NO	YES
VAGINAL BIRTH	YES	NO	MALE	5	9	3940	NO	NO	YES	NO	YES	NO	NO	NO	NO	YES	9,86	NO	YES	NO
VAGINAL BIRTH	YES	NO	MALE	8	10	3325	NO	NO	NO	NO	YES	NO	NO	NO	NO	NO	9,86	NO	NO	NO
C-SECTIONYES	NO	FEMALE	7	7	2980	NO	YES	NO	NO	YES	NO	NO	NO	NO	NO	9,86	NO	NO	NO	
C-SECTIONYES	NO	MALE	8	9	3225	YES	NO	NO	NO	YES	NO	NO	NO	NO	NO	9,86	NO	NO	NO	
VAGINAL BIRTH	YES	NO	MALE	9	10	3500	NO	YES	NO	NO	YES	NO	NO	NO	NO	NO	9,86	NO	YES	NO
C-SECTIONNO	NO	MALE	9	9	2335	NO	NO	NO	NO	YES	NO	NO	NO	NO	NO	9,86	NO	NO	NO	
C-SECTIONNO	NO	MALE	9	10	3180	NO	NO	NO	NO	YES	NO	YES	NO	NO	NO	9,86	NO	NO	NO	
C-SECTIONNO	NO	MALE	8	9	3530	NO	NO	NO	NO	YES	NO	NO	NO	NO	NO	9,86	NO	NO	NO	
C-SECTIONNO	NO	FEMALE	6	8	3000	NO	NO	NO	NO	YES	NO	NO	NO	NO	YES	9,86	NO	YES	YES	
VAGINAL BIRTH	YES	NO	FEMALE	9	9	3410	NO	YES	NO	NO	YES	NO	NO	NO	NO	NO	9,86	NO	NO	YES
VAGINAL BIRTH	YES	NO	MALE	9	9	3360	NO	NO	NO	NO	YES	NO	NO	NO	NO	NO	9,86	NO	NO	YES
VAGINAL BIRTH	YES	NO	MALE	7	9	3465	YES	NO	YES	NO	YES	NO	NO	NO	NO	NO	10,00	NO	NO	NO
C-SECTIONYES	NO	MALE	9	9	4275	NO	NO	NO	NO	YES	NO	NO	NO	NO	NO	10,00	NO	YES	NO	
VAGINAL BIRTH	NO	NO	FEMALE	8	9	3955	NO	NO	NO	NO	YES	NO	NO	NO	NO	NO	10,00	NO	NO	NO
VAGINAL BIRTH	YES	NO	MALE	0	0	1315		NO	NO	NO	YES	NO	NO	YES	NO	NO	10,00	NO	NO	NO
VAGINAL BIRTH	YES	NO		0	0	1885		NO	NO	NO	YES	NO	NO	YES	NO	NO	10,00	NO	NO	NO

C-SECTION	YES	NO	MALE	4	7	1535	YES	NO	NO	NO	YES	NO	NO	NO	NO	YES	10,00	NO	NO	NO	
C-SECTION	YES	NO	FEMALE	7	9	2005	YES	YES	NO	NO	YES	NO	NO	NO	NO	NO	10,00	NO	NO	NO	
C-SECTION	YES	NO	FEMALE	9	10	2590	NO	NO	NO	NO	YES	NO	NO	NO	NO	NO	10,00	NO	NO	NO	
C-SECTION	YES	NO	MALE	6	10	3420	NO	NO	YES	NO	YES	NO	NO	NO	NO	YES	10,00	NO	NO	NO	
C-SECTION	NO	NO	FEMALE	9	10	2540	NO	NO	NO	NO	YES	NO	NO	NO	NO	NO	10,00	NO	NO	NO	
VAGINAL BIRTH		YES	NO	FEMALE	9	9	2945	NO	YES	NO	NO	YES	NO	NO	NO	NO	NO	10,00	NO	YES	YES
C-SECTION	YES	NO	MALE	9	10	3630	NO	NO	YES	NO	YES	NO	NO	NO	NO	NO	10,00	NO	NO	YES	
VAGINAL BIRTH		NO	NO	FEMALE	9	10	2820	NO	NO	NO	NO	YES	NO	NO	NO	NO	NO	10,00	NO	NO	YES
VAGINAL BIRTH		YES	NO	FEMALE	9	10	2465	NO	YES	NO	NO	YES	NO	NO	NO	NO	NO	10,00	NO	NO	YES
VAGINAL BIRTH		YES	NO	FEMALE	8	9	3420	NO	YES	NO	NO	YES	NO	NO	NO	NO	NO	10,00	NO	NO	YES
C-SECTION	NO	NO	MALE	9	9	3415	NO	NO	NO	NO	YES	NO	NO	NO	NO	NO	10,00	NO	NO	YES	
VAGINAL BIRTH		NO	NO	FEMALE	9	9	2615	NO	NO	NO	NO	YES	NO	NO	NO	NO	NO	10,00	NO	NO	YES
VAGINAL BIRTH		NO	NO	MALE	9	9	3140	NO	NO	NO	NO	YES	NO	NO	NO	NO	NO	10,00	NO	NO	YES
VAGINAL BIRTH		NO	NO	MALE	8	9	3185	NO	NO	NO	NO	YES	NO	NO	NO	NO	NO	10,00	NO	NO	YES
C-SECTION	YES	NO	FEMALE	3	7	1070	YES	NO	NO	NO	YES	NO	NO	YES	NO	YES	10,00	NO	NO	YES	
VAGINAL BIRTH		NO	NO	FEMALE	9	10	3530	NO	NO	NO	NO	YES	NO	NO	NO	NO	NO	10,14	NO	NO	NO
VAGINAL BIRTH		YES	NO	FEMALE	8	9	3015	NO	NO	YES	NO	YES	NO	NO	NO	NO	NO	10,14	NO	YES	NO
C-SECTION	YES	NO	MALE	8	9	1070	YES	YES	NO	NO	YES	NO	NO	NO	YES	NO	10,14	NO	NO	NO	
C-SECTION	YES	NO	MALE	9	9	1290	YES	YES	NO	NO	YES	NO	NO	NO	NO	NO	10,14	NO	NO	NO	
VAGINAL BIRTH		NO	NO	MALE	9	10	3045	NO	NO	NO	NO	YES	NO	NO	NO	NO	NO	10,14	NO	NO	YES
C-SECTION	NO	NO	FEMALE	4	8	2940	NO	NO	NO	NO	YES	NO	NO	NO	NO	YES	10,14	NO	NO	YES	
VAGINAL BIRTH		NO	NO	MALE	9	10	2765	NO	NO	NO	NO	YES	NO	NO	NO	NO	NO	10,14	NO	NO	YES
C-SECTION	NO	NO	MALE	8	9	3635	NO	NO	NO	NO	YES	NO	NO	NO	NO	NO	10,14	NO	NO	YES	
VAGINAL BIRTH		YES	NO	FEMALE	8	9	3225	NO	NO	NO	NO	YES	NO	NO	NO	NO	NO	10,14	NO	NO	YES
C-SECTION	YES	NO	MALE	9	9	2870	NO	YES	NO	NO	YES	NO	NO	NO	NO	NO	10,14	NO	NO	YES	
VAGINAL BIRTH		NO	NO	FEMALE	9	10	2830	NO	NO	NO	NO	YES	NO	NO	NO	NO	NO	10,14	NO	NO	YES
VAGINAL BIRTH		YES	NO	MALE	0	0	650		NO	NO	NO	YES	NO	NO	YES	NO	NO	10,29	NO	NO	NO
C-SECTION	YES	NO	FEMALE	8	9	2325	NO	NO	NO	YES	YES	NO	NO	NO	NO	NO	10,29	NO	NO	NO	
C-SECTION	NO	NO	MALE	8	8	3610	NO	NO	NO	NO	YES	NO	NO	NO	NO	NO	10,29	NO	NO	NO	
C-SECTION	YES	NO	FEMALE	10	10	2895	NO	YES	NO	NO	YES	NO	NO	NO	NO	NO	10,29	NO	NO	NO	
C-SECTION	YES	NO	MALE	9	9	2990	NO	NO	NO	YES	YES	NO	NO	NO	NO	NO	10,29	NO	NO	NO	
C-SECTION	YES	NO	MALE	3	7	920	YES	NO	NO	NO	YES			NO	NO	YES	10,29	NO	NO	NO	
VAGINAL BIRTH		NO	NO	MALE	9	10	3845	NO	NO	NO	NO	YES	NO	NO	NO	NO	NO	10,29	NO	YES	YES
VAGINAL BIRTH		NO	NO	MALE	9	10	2980	NO	NO	NO	NO	YES	NO	NO	NO	NO	NO	10,29	NO	YES	YES
VAGINAL BIRTH		NO	NO	MALE	9	10	3245	NO	NO	NO	NO	YES	NO	NO	NO	NO	NO	10,29	NO	YES	YES
VAGINAL BIRTH		YES	NO	FEMALE	9	9	3060	NO	YES	NO	NO	YES	NO	NO	NO	NO	NO	10,29	NO	NO	YES
VAGINAL BIRTH		NO	NO	MALE	9	10	2720	NO	NO	NO	NO	YES	NO	NO	NO	NO	NO	10,29	NO	NO	YES
C-SECTION	NO	NO	FEMALE	10	10	2765	NO	NO	NO	NO	YES	NO	NO	NO	NO	NO	10,29	NO	NO	YES	
VAGINAL BIRTH		NO	NO	FEMALE	9	9	3570	NO	NO	NO	NO	YES	NO	NO	NO	NO	NO	10,29	NO	NO	YES
C-SECTION	YES	NO	MALE	5	9	910	YES	NO	NO	NO	YES	NO	NO	YES	NO	YES	10,29	NO	NO	YES	
VAGINAL BIRTH		YES	NO	MALE	8	9	3100	YES	NO	NO	NO	YES	YES	YES	NO	YES	NO	10,43	NO	YES	NO
VAGINAL BIRTH		NO	NO	MALE	9	9	2940	NO	NO	NO	NO	YES	NO	NO	NO	NO	NO	10,43	NO	NO	NO
VAGINAL BIRTH		NO	NO	FEMALE	8	9	2715	NO	NO	NO	NO	YES	NO	NO	NO	NO	NO	10,43	NO	NO	NO
C-SECTION	YES	NO	MALE	9	9	2405	NO	YES	YES	NO	YES	NO	NO	NO	NO	NO	10,43	NO	NO	NO	
VAGINAL BIRTH		YES	NO	FEMALE	9	10	3200	NO	YES	NO	NO	YES	NO	NO	NO	NO	NO	10,43	NO	NO	NO
C-SECTION	NO	NO	MALE	8	9	4280	NO	NO	NO	NO	YES			NO	NO	NO	10,43	NO	NO	NO	
VAGINAL BIRTH		YES	NO	FEMALE	7	9	2780	NO	YES	NO	NO	YES	NO	NO	NO	NO	NO	10,43	NO	YES	NO
VAGINAL BIRTH		YES	NO	MALE	9	9	3215	NO	NO	NO	NO	YES	NO	NO	NO	NO	NO	10,43	NO	NO	NO
VAGINAL BIRTH		YES	NO	FEMALE	9	9	2600	NO	NO	NO	NO	YES	NO	NO	NO	NO	NO	10,43	NO	NO	NO
C-SECTION	YES	NO	MALE	6	7	1880	YES	YES	NO	NO	YES	NO	NO	NO	NO	YES	10,43	NO	NO	NO	
C-SECTION	YES	NO	FEMALE	9	9	2815	NO	NO	NO	NO	YES	NO	NO	NO	NO	NO	10,43	NO	NO	NO	
C-SECTION	YES	NO	MALE	9	10	3045	NO	NO	NO	NO	YES	NO	NO	NO	NO	NO	10,43	NO	NO	NO	
C-SECTION	YES	NO	MALE	9	10	3420	NO	YES	NO	NO	YES	NO	NO	NO	NO	NO	10,43	NO	NO	NO	

C-SECTIONYES	NO	FEMALE	3	6	2645	YES	NO	YES	NO	YES	NO	NO	NO	NO	YES	10,43	NO	NO	NO	
VAGINAL BIRTH	YES	NO	FEMALE	9	9	3040	YES	YES	NO	NO	YES	NO	NO	NO	NO	NO	10,43	NO	NO	NO
C-SECTIONYES	NO	FEMALE	8	10	2985	YES	NO	NO	NO	YES	YES	YES	NO	NO	NO	10,43	NO	NO	YES	
VAGINAL BIRTH	YES	NO	MALE	9	10	3190	NO	NO	NO	NO	YES	NO	NO	NO	NO	NO	10,43	NO	NO	YES
VAGINAL BIRTH	YES	NO	MALE	9	10	3020	NO	NO	NO	NO	YES	NO	NO	NO	NO	NO	10,43	NO	NO	YES
VAGINAL BIRTH	NO	NO	MALE	9	10	2900	NO	NO	NO	NO	YES	NO	NO	NO	NO	NO	10,43	NO	NO	YES
VAGINAL BIRTH	NO	YES	FEMALE	8	9	3090	NO	NO	NO	NO	YES	NO	NO	NO	NO	NO	10,43	NO	NO	YES
VAGINAL BIRTH	NO	NO	FEMALE	8	9	2985	NO	NO	NO	NO	YES	NO	NO	NO	NO	NO	10,43	NO	NO	YES
VAGINAL BIRTH	NO	NO	FEMALE	9	10	2985	NO	NO	NO	NO	YES	NO	NO	NO	NO	NO	10,43	NO	NO	YES
VAGINAL BIRTH	NO	NO	FEMALE	9	9	3055	NO	NO	NO	NO	YES	NO	NO	NO	NO	NO	10,43	NO	NO	YES
VAGINAL BIRTH	NO	NO	FEMALE	9	9	2760	NO	NO	NO	NO	YES	NO	NO	NO	NO	NO	10,43	NO	NO	YES
VAGINAL BIRTH	YES	NO	MALE	8	9	2335	NO	YES	NO	NO	YES	NO	NO	NO	NO	NO	10,43	NO	NO	YES
VAGINAL BIRTH	YES	NO	FEMALE	7	9	2105	NO	YES	NO	NO	YES	NO	YES	NO	NO	NO	10,57	NO	YES	NO
VAGINAL BIRTH	YES	NO	MALE	1	9	3240	YES	NO	YES	NO	YES	NO	NO	NO	NO	YES	10,57	NO	YES	NO
VAGINAL BIRTH	YES	YES	MALE	9	9	3410	NO	NO	NO	NO	YES	NO	NO	NO	NO	NO	10,57	NO	YES	NO
VAGINAL BIRTH	YES	NO	FEMALE	9	10	3130	NO	NO	YES	NO	YES	NO	NO	NO	NO	NO	10,57	NO	NO	NO
VAGINAL BIRTH	YES	NO	MALE	9	9	2435	NO	YES	NO	NO	YES	NO	NO	NO	NO	NO	10,57	NO	NO	NO
VAGINAL BIRTH	YES	NO	MALE	8	9	3605	NO	YES	NO	NO	YES	NO	NO	NO	NO	NO	10,57	NO	NO	NO
VAGINAL BIRTH	NO	NO	MALE	9	10	2680	NO	NO	NO	NO	YES	NO	NO	NO	NO	NO	10,57	NO	YES	NO
C-SECTIONNO	NO	MALE	8	9	3585	NO	NO	NO	NO	YES	NO	NO	NO	NO	NO	10,57	NO	NO	NO	
C-SECTIONYES	NO	FEMALE	8	9	975	YES	YES	NO	NO	YES	NO	NO	NO	NO	NO	10,57	NO	NO	NO	
C-SECTIONNO	NO	MALE	9	9	3270	NO	NO	NO	NO	YES	NO	NO	NO	NO	NO	10,57	NO	NO	NO	
C-SECTIONNO	NO	MALE	8	9	4555	NO	NO	NO	NO	YES	NO	NO	NO	NO	NO	10,57	NO	NO	NO	
C-SECTIONNO	NO	FEMALE	8	9	3640	NO	NO	NO	NO	YES	NO	NO	NO	NO	NO	10,57	NO	NO	NO	
C-SECTIONYES	NO	FEMALE	7	9	2515	NO	NO	NO	NO	YES	NO	NO	NO	NO	NO	10,57	NO	NO	NO	
C-SECTIONYES	NO	FEMALE	0	0	1370		YES	YES	NO	YES	NO	NO	YES	NO	NO	10,57	NO	NO	NO	
VAGINAL BIRTH	NO	NO	MALE	7	9	2900	NO	NO	NO	NO	YES	NO	YES	NO	NO	NO	10,57	NO	NO	YES
VAGINAL BIRTH	YES	NO	FEMALE	9	9	2045	YES	NO	NO	NO	YES	NO	NO	NO	NO	NO	10,57	NO	NO	YES
VAGINAL BIRTH	NO	NO	MALE	8	9	2425	NO	NO	NO	NO	YES	NO	NO	NO	NO	NO	10,57	NO	YES	YES
VAGINAL BIRTH	YES	NO	FEMALE	9	10	2800	NO	NO	NO	NO	YES	NO	NO	NO	YES	NO	10,57	NO	NO	YES
VAGINAL BIRTH	NO	NO	MALE	9	10	2945	NO	NO	NO	NO	YES	NO	NO	NO	NO	NO	10,57	NO	NO	YES
VAGINAL BIRTH	NO	NO	MALE	8	9	3415	NO	NO	NO	NO	YES	NO	NO	NO	NO	NO	10,57	NO	YES	YES
VAGINAL BIRTH	NO	NO	FEMALE	8	9	2675	NO	NO	NO	NO	YES	NO	NO	NO	NO	NO	10,57	NO	NO	YES
VAGINAL BIRTH	YES	NO	FEMALE	9	10	3485	NO	NO	NO	NO	YES	NO	NO	NO	NO	NO	10,57	NO	NO	YES
C-SECTIONNO	NO	MALE	9	10	3650	NO	NO	NO	NO	YES	NO	NO	NO	NO	NO	10,57	NO	NO	YES	
VAGINAL BIRTH	NO	NO	FEMALE	9	9	2945	NO	NO	NO	NO	YES	NO	NO	NO	NO	NO	10,57	NO	NO	YES
C-SECTIONYES	NO	MALE	8	9	3235	NO	NO	NO	NO	YES	NO	NO	NO	NO	NO	10,71	NO	NO	NO	
VAGINAL BIRTH	YES	NO	MALE	0	0	1205		NO	NO	NO	YES	NO	NO	NO	NO	NO	10,71	NO	YES	NO
VAGINAL BIRTH	YES	NO	FEMALE	7	8	2775	NO	YES	NO	NO	YES	NO	NO	NO	NO	NO	10,71	NO	NO	NO
VAGINAL BIRTH	NO	NO	MALE	9	10	3830	NO	NO	NO	NO	YES	NO	NO	NO	NO	NO	10,71	NO	NO	NO
C-SECTIONYES	NO	MALE	8	9	3285	NO	NO	YES	NO	YES	NO	NO	NO	NO	NO	10,71	NO	NO	NO	
C-SECTIONYES	NO	MALE	4	6	930	YES	YES	NO	NO	YES	NO	NO	NO	NO	YES	10,71	NO	NO	NO	
C-SECTIONYES	NO	MALE	8	7	2255	YES	NO	NO	NO	YES	YES	NO	NO	NO	NO	10,71	NO	NO	NO	
C-SECTIONNO	NO	MALE	9	9	2480	NO	NO	NO	NO	YES	NO	NO	NO	NO	NO	10,71	NO	NO	NO	
VAGINAL BIRTH	YES	NO	MALE	9	10	2980	YES	YES	NO	NO	YES	NO	NO	NO	NO	NO	10,71	NO	NO	NO
VAGINAL BIRTH	NO	NO	FEMALE	8	9	2940	NO	NO	NO	NO	YES	NO	NO	NO	NO	NO	10,71	NO	NO	YES
VAGINAL BIRTH	YES	NO	MALE	8	9	2705	NO	NO	NO	NO	YES	NO	NO	NO	NO	NO	10,71	NO	YES	YES
VAGINAL BIRTH	NO	NO	MALE	8	9	4070	NO	NO	NO	NO	YES	NO	NO	NO	NO	NO	10,71	NO	YES	YES
VAGINAL BIRTH	NO	YES	MALE	7	9	3115	NO	NO	NO	NO	YES	NO	NO	NO	NO	NO	10,71	NO	NO	YES
VAGINAL BIRTH	NO	NO	FEMALE	7	9	2750	NO	NO	NO	NO	YES	NO	NO	NO	NO	NO	10,71	NO	NO	YES
C-SECTIONNO	NO	MALE	9	9	3320	NO	NO	NO	NO	YES	NO	NO	NO	NO	NO	10,86	NO	NO	NO	
VAGINAL BIRTH	NO	NO	MALE	9	9	2725	NO	NO	NO	NO	YES	NO	NO	NO	NO	NO	10,86	NO	YES	NO
VAGINAL BIRTH	NO	NO	FEMALE	8	9	2210	NO	NO	NO	NO	YES	NO	NO	NO	NO	NO	10,86	NO	NO	NO

VAGINAL BIRTH		YES	NO	MALE	2	6	3190	YES	YES	NO	NO	YES	NO	NO	NO	NO	YES	10,86	NO	YES	NO
C-SECTION	YES	NO	MALE	9	10	3200	NO	NO	NO	YES	YES	NO	NO	NO	NO	NO	10,86	NO	NO	NO	
C-SECTION	YES	NO	MALE	9	10	3300	NO	NO	NO	YES	YES	NO	NO	NO	NO	NO	10,86	NO	NO	NO	
C-SECTION	YES	NO	FEMALE	9	9	3550	NO	NO	NO	NO	YES	NO	NO	NO	NO	NO	10,86	NO	NO	NO	
C-SECTION	NO	NO	FEMALE	8	10	2735	NO	NO	NO	NO	YES	NO	NO	NO	NO	NO	10,86	NO	NO	NO	
C-SECTION	YES	NO	FEMALE	9	9	2585	NO	NO	YES	NO	YES	NO	NO	NO	NO	NO	10,86	NO	NO	NO	
C-SECTION	YES	NO	FEMALE	9	10	2770	NO	NO	YES	NO	YES	NO	NO	NO	NO	NO	10,86	NO	NO	NO	
C-SECTION	YES	NO	MALE	9	10	2700	NO	NO	NO	NO	YES	NO	NO	NO	YES	NO	10,86	NO	NO	NO	
C-SECTION	YES	NO	MALE	9	9	3055	NO	NO	YES	NO	YES	NO	NO	NO	NO	NO	10,86	NO	NO	NO	
C-SECTION	YES	NO	MALE	8	8	3050	YES	YES	YES	YES	YES	NO	NO	NO	NO	NO	10,86	NO	NO	NO	
C-SECTION	YES	NO	FEMALE	8	8	2870	NO	YES	YES	YES	YES	NO	NO	NO	NO	NO	10,86	NO	NO	NO	
VAGINAL BIRTH		YES	NO	FEMALE	9	9	2870	NO	NO	YES	NO	YES	NO	NO	NO	NO	NO	10,86	NO	NO	YES
C-SECTION	YES	NO	FEMALE	8	9	3605	NO	NO	YES	NO	YES	NO	NO	NO	NO	NO	10,86	NO	NO	YES	
VAGINAL BIRTH		YES	NO	MALE	9	9	3260	NO	NO	YES	NO	YES	NO	NO	NO	NO	NO	11,00	NO	NO	YES
VAGINAL BIRTH		YES	NO	FEMALE	9	9	2025	NO	NO	YES	NO	YES	NO	NO	NO	NO	NO	11,00	NO	NO	NO
VAGINAL BIRTH		YES	NO	FEMALE	9	9	3060	YES	NO	YES	NO	YES	NO	NO	NO	NO	NO	11,00	NO	NO	NO
C-SECTION	NO	NO	MALE	5	9	3910	NO	NO	NO	NO	YES	NO	NO	NO	NO	YES	11,00	NO	YES	NO	
VAGINAL BIRTH		NO	NO	MALE	9	10	3320	NO	NO	NO	NO	YES	NO	NO	NO	NO	NO	11,00	NO	NO	NO
C-SECTION	YES	NO	FEMALE	8	9	3345	NO	YES	NO	NO	YES	NO	NO	NO	NO	NO	11,00	NO	NO	NO	
VAGINAL BIRTH		NO	NO	FEMALE	9	9	3400	NO	NO	NO	NO	YES	NO	NO	NO	NO	NO	11,00	NO	NO	NO
C-SECTION	YES	NO	MALE	9	9	3130	NO	NO	NO	NO	YES	NO	NO	NO	NO	NO	11,00	NO	NO	NO	
C-SECTION	YES	NO	FEMALE	9	10	2705	NO	NO	NO	NO	YES	NO	NO	NO	YES	NO	11,00	NO	NO	NO	
VAGINAL BIRTH		NO	NO	MALE	9	10	2405	NO	NO	NO	NO	YES	NO	NO	NO	NO	NO	11,00	NO	NO	YES
VAGINAL BIRTH		YES	NO	FEMALE	9	10	3210	NO	NO	NO	NO	YES	NO	NO	NO	YES	NO	11,00	NO	NO	YES
VAGINAL BIRTH		NO	NO	FEMALE	9	9	2875	NO	NO	NO	NO	YES	NO	NO	NO	NO	NO	11,00	NO	NO	YES
VAGINAL BIRTH		YES	NO	FEMALE	9	9	3140	NO	YES	NO	NO	YES	NO	NO	NO	NO	NO	11,00	NO	YES	YES
VAGINAL BIRTH		NO	NO	MALE	8	9	3470	NO	NO	NO	NO	YES	NO	NO	NO	NO	NO	11,00	NO	NO	YES
VAGINAL BIRTH		YES	NO	MALE	9	10	2805	NO	NO	NO	NO	YES	NO	NO	NO	YES	NO	11,00	NO	NO	YES
VAGINAL BIRTH		NO	NO	FEMALE	9	10	2975	NO	NO	NO	NO	YES	NO	NO	NO	NO	NO	11,00	NO	NO	YES
VAGINAL BIRTH		NO	NO	FEMALE	9	10	3320	NO	NO	NO	NO	YES	NO	NO	NO	NO	NO	11,00	NO	YES	YES
VAGINAL BIRTH		NO	NO	FEMALE	10	10	2860	NO	NO	NO	NO	YES	NO	NO	NO	NO	NO	11,00	NO	NO	YES
VAGINAL BIRTH		YES	YES	FEMALE	8	9	2960	NO	NO	NO	NO	YES	NO	NO	NO	YES	NO	11,14	NO	YES	NO
VAGINAL BIRTH		YES	NO	FEMALE	8	9	3420	NO	NO	NO	NO	YES	NO	NO	NO	NO	NO	11,14	NO	NO	NO
C-SECTION	YES	NO	MALE	9	10	3265	NO	NO	YES	NO	YES	NO	NO	NO	NO	NO	11,14	NO	NO	NO	
VAGINAL BIRTH		NO	NO	MALE	8	9	3575	NO	NO	NO	NO	YES	NO	NO	NO	NO	NO	11,14	NO	YES	NO
C-SECTION	YES	NO	MALE	8	10	1990	NO	NO	NO	NO	YES	NO	NO	NO	NO	NO	11,14	NO	NO	NO	
C-SECTION	YES	NO	FEMALE	9	10	3110	NO	NO	YES	NO	YES	NO	NO	NO	NO	NO	11,14	NO	NO	NO	
C-SECTION	YES	NO	MALE	9	10	4430	NO	YES	NO	NO	YES	NO	NO	NO	NO	NO	11,14	NO	NO	NO	
VAGINAL BIRTH		NO	NO	MALE	10	10	2770	NO	NO	NO	NO	YES	NO	NO	NO	NO	NO	11,14	NO	NO	YES
VAGINAL BIRTH		YES	NO	MALE	9	10	3370	NO	NO	NO	NO	YES	NO	NO	NO	NO	NO	11,14	NO	NO	YES
VAGINAL BIRTH		NO	NO	FEMALE	9	10	3085	NO	NO	NO	NO	YES	NO	NO	NO	NO	NO	11,14	NO	NO	YES
C-SECTION	NO	NO	FEMALE	9	9	3175	NO	NO	NO	NO	YES	NO	NO	NO	NO	NO	11,14	NO	NO	YES	
C-SECTION	NO	NO	MALE	6	9	3145	NO	NO	NO	NO	YES	NO	NO	NO	NO	YES	11,14	NO	NO	YES	
VAGINAL BIRTH		YES	NO	FEMALE	3	7	625	YES	NO	NO	NO	YES	NO	NO	YES	NO	YES	11,14	NO	NO	YES
VAGINAL BIRTH		NO	NO	MALE	6	8	3415	NO	NO	NO	NO	YES	NO	NO	NO	NO	NO	11,14	NO	NO	YES
VAGINAL BIRTH		NO	NO	FEMALE	9	10	3330	NO	NO	NO	NO	YES	NO	NO	NO	NO	NO	11,14	NO	NO	YES
VAGINAL BIRTH		NO	NO	MALE	9	10	3335	NO	NO	NO	NO	YES	NO	NO	NO	NO	NO	11,14	NO	NO	YES
VAGINAL BIRTH		YES	NO	MALE	9	10	3165	NO	NO	NO	NO	YES	NO	NO	NO	YES	NO	11,14	NO	NO	YES
VAGINAL BIRTH		YES	NO	MALE	9	10	3150	NO	NO	NO	NO	YES	NO	NO	NO	NO	NO	11,29	NO	NO	NO
VAGINAL BIRTH		YES	NO	FEMALE	9	9	3400	NO	YES	NO	NO	YES	NO	NO	NO	NO	NO	11,29	NO	YES	NO
VAGINAL BIRTH		YES	NO	MALE	9	10	2725	NO	YES	NO	NO	YES	NO	NO	NO	NO	NO	11,29	NO	NO	NO
VAGINAL BIRTH		YES	NO	FEMALE	8	9	3725	NO	NO	NO	NO	YES	NO	NO	NO	YES	NO	11,29	NO	NO	NO
C-SECTION	NO	NO	FEMALE	8	8	3475	NO	NO	NO	NO	YES	NO	NO	NO	NO	NO	11,29	NO	NO	NO	

C-SECTION	NO	NO	MALE	8	9	3490	NO	NO	NO	NO	YES	NO	NO	NO	NO	NO	11,29	NO	NO	NO	
VAGINAL BIRTH		NO	NO	MALE	8	9	4185	NO	NO	NO	NO	YES	NO	NO	NO	NO	NO	11,29	NO	YES	NO
C-SECTION	YES	NO	FEMALE	8	9	1540	NO	YES	NO	NO	YES	NO	NO	NO	NO	NO	11,29	NO	NO	NO	
C-SECTION	YES	NO	MALE	8	9	3080	YES	NO	YES	NO	YES	NO	NO	NO	NO	NO	11,29	NO	NO	NO	
VAGINAL BIRTH		YES	NO	MALE	8	7	1610	YES	NO	NO	NO	YES	NO	NO	NO	NO	NO	11,29	NO	NO	YES
C-SECTION	YES	NO	MALE	7	8	2815	YES	NO	YES	NO	YES	NO	NO	NO	NO	NO	11,29	NO	NO	YES	
VAGINAL BIRTH		YES	NO	FEMALE	9	10	2805	NO	NO	NO	NO	YES	NO	NO	NO	NO	NO	11,29	NO	YES	YES
VAGINAL BIRTH		NO	NO	MALE	3	7	4090	YES	NO	NO	NO	YES	NO	NO	NO	NO	YES	11,29	NO	NO	YES
VAGINAL BIRTH		NO	NO	FEMALE	8	9	3250	NO	NO	NO	NO	YES	NO	NO	NO	NO	NO	11,29	NO	NO	YES
VAGINAL BIRTH		NO	NO	FEMALE	8	9	3255	NO	NO	NO	NO	YES	NO	NO	NO	NO	NO	11,29	NO	NO	YES
VAGINAL BIRTH		NO	NO	MALE	9	9	3150	NO	NO	NO	NO	YES	NO	NO	NO	NO	NO	11,29	NO	NO	YES
VAGINAL BIRTH		YES	NO				310		YES	NO	NO	YES	NO	NO	NO	NO	NO	11,43	NO	NO	YES
VAGINAL BIRTH		YES	NO	MALE	0	0	740		NO	YES	NO	YES	NO	NO	YES	NO	NO	11,43	NO	NO	NO
VAGINAL BIRTH		YES	NO	MALE	9	10	2350	NO	NO	NO	NO	YES	NO	NO	NO	NO	NO	11,43	NO	NO	NO
VAGINAL BIRTH		YES	NO	MALE	8	9	3245	NO	NO	YES	NO	YES	NO	NO	NO	NO	NO	11,43	NO	NO	NO
VAGINAL BIRTH		YES	NO	FEMALE	9	9	2855	NO	YES	NO	NO	YES	NO	NO	NO	NO	NO	11,43	NO	NO	NO
VAGINAL BIRTH		YES	NO	FEMALE	9	10	2760	NO	NO	NO	NO	YES	NO	NO	NO	NO	NO	11,43	NO	YES	NO
VAGINAL BIRTH		NO	NO	MALE	9	10	3180	NO	NO	NO	NO	YES	NO	NO	NO	NO	NO	11,43	NO	YES	NO
C-SECTION	NO	NO	MALE	9	9	4160	NO	NO	NO	NO	YES	NO	NO	NO	NO	NO	11,43	NO	YES	NO	
VAGINAL BIRTH		NO	NO	MALE	7	9	3245	NO	NO	NO	NO	YES	NO	NO	NO	NO	NO	11,43	NO	NO	NO
VAGINAL BIRTH		YES	NO	FEMALE	8	10	2630	NO	YES	NO	NO	YES	NO	NO	NO	NO	NO	11,43	NO	NO	NO
C-SECTION	NO	NO	MALE	8	9	3665	NO	NO	NO	NO	YES	NO	NO	NO	NO	NO	11,43	NO	NO	NO	
C-SECTION	YES	NO	MALE	9	9	2665	NO	NO	NO	NO	YES	NO	NO	NO	NO	NO	11,43	NO	NO	NO	
C-SECTION	YES	NO	MALE	9	9	3180	NO	NO	NO	NO	YES	NO	NO	NO	NO	NO	11,43	NO	NO	NO	
C-SECTION	YES	NO	MALE	7	9	2665	NO	NO	NO	NO	YES	NO	NO	NO	NO	NO	11,43	NO	NO	NO	
C-SECTION	NO	NO	FEMALE	8	9	3475	NO	NO	NO	NO	YES	NO	NO	NO	NO	NO	11,43	NO	NO	NO	
C-SECTION	YES	NO	MALE	8	10	2780	YES	NO	NO	NO	YES	NO	NO	NO	NO	NO	11,43	NO	NO	NO	
C-SECTION	YES	NO	MALE	8	9	4615	NO	YES	NO	NO	YES	NO	NO	NO	NO	NO	11,43	NO	NO	NO	
C-SECTION	NO	NO	FEMALE	8	9	3395	NO	NO	NO	NO	YES	NO	NO	NO	NO	NO	11,43	NO	NO	NO	
C-SECTION	NO	NO	MALE	9	10	3950	NO	NO	NO	NO	YES	NO	NO	NO	NO	NO	11,43	NO	NO	NO	
C-SECTION	YES	NO	FEMALE	6	9	1190	YES	YES	NO	NO	YES	NO	NO	NO	NO	YES	11,43	NO	NO	NO	
C-SECTION	NO	NO	MALE	10	10	4070	NO	NO	NO	NO	YES	NO	YES	NO	NO	NO	11,43	NO	YES	YES	
VAGINAL BIRTH		NO	NO	FEMALE	8	9	3390	NO	NO	NO	NO	YES	NO	NO	NO	NO	NO	11,43	NO	NO	YES
VAGINAL BIRTH		NO	NO	FEMALE	9	10	3065	NO	NO	NO	NO	YES	NO	NO	NO	NO	NO	11,43	NO	NO	YES
C-SECTION	NO	NO	FEMALE	6	9	3145	NO	NO	NO	NO	YES	NO	NO	NO	NO	YES	11,43	NO	NO	YES	
C-SECTION	YES	NO	FEMALE	8	9	1750	YES	YES	NO	NO	YES	NO	NO	NO	YES	NO	11,43	NO	NO	YES	
VAGINAL BIRTH		NO	NO	MALE	7	9	2270	NO	NO	NO	NO	YES	NO	NO	NO	NO	NO	11,43	NO	NO	YES
C-SECTION	YES	NO	MALE	8	8	2540	YES	NO	NO	YES	YES	NO	NO	NO	NO	NO	11,43	NO	YES	YES	
C-SECTION	YES	NO	FEMALE	9	9	2595	NO	NO	NO	YES	YES	NO	NO	NO	NO	NO	11,43	NO	YES	YES	
VAGINAL BIRTH		NO	NO	FEMALE	9	9	3170	YES	NO	NO	NO	YES	NO	NO	NO	NO	NO	11,43	NO	YES	YES
VAGINAL BIRTH		YES	NO	MALE	8	9	2605	NO	YES	NO	NO	YES	NO	NO	NO	NO	NO	11,43	NO	YES	YES
C-SECTION	YES	NO	FEMALE	8	9	2605	NO	YES	NO	NO	YES	NO	NO	NO	NO	NO	11,57	NO	NO	NO	
C-SECTION	NO	NO	MALE	7	9	3820	NO	NO	NO	NO	YES	NO	NO	NO	NO	NO	11,57	NO	NO	NO	
C-SECTION	YES	NO	FEMALE	6	8	3200	NO	YES	NO	NO	YES	NO	NO	NO	NO	YES	11,57	NO	NO	NO	
C-SECTION	NO	NO	FEMALE	8	9	3180	NO	NO	NO	NO	YES	NO	NO	NO	NO	NO	11,57	NO	NO	NO	
VAGINAL BIRTH		YES	NO	MALE	9	10	3610	NO	NO	NO	NO	YES	NO	NO	NO	NO	NO	11,57	NO	NO	NO
C-SECTION	YES	NO	MALE	9	9	2145	NO	NO	NO	NO	YES	NO	NO	NO	YES	NO	11,57	NO	NO	NO	
C-SECTION	NO	NO	FEMALE	9	10	3045	NO	NO	NO	NO	YES	NO	NO	NO	NO	NO	11,57	NO	NO	NO	
C-SECTION	NO	NO	MALE	9	9	3510	NO	NO	NO	NO	YES	NO	NO	NO	NO	NO	11,57	NO	NO	NO	
C-SECTION	YES	NO	MALE	8	9	2505	YES	NO	NO	NO	YES	NO	NO	NO	NO	NO	11,57	NO	NO	NO	
C-SECTION	YES	NO	FEMALE	9	10	3255	NO	YES	NO	NO	YES	NO	NO	NO	NO	NO	11,57	NO	NO	NO	
C-SECTION	YES	NO	FEMALE	9	10	3160	NO	YES	NO	NO	YES	NO	NO	NO	NO	NO	11,57	NO	NO	NO	
C-SECTION	NO	NO	MALE	6	9	2865	NO	NO	NO	NO	YES	NO	NO	NO	NO	YES	11,57	NO	NO	NO	

C-SECTIONNO	NO	FEMALE	8	9	3600	NO	NO	NO	NO	YES	NO	NO	NO	NO	NO	11,57	NO	NO	NO	
VAGINAL BIRTH	YES	NO	FEMALE	9	9	2560	NO	YES	NO	NO	YES	NO	NO	NO	NO	NO	11,57	NO	NO	YES
VAGINAL BIRTH	NO	NO	MALE	8	9	2840	NO	NO	NO	NO	YES	NO	NO	NO	NO	NO	11,57	NO	NO	YES
VAGINAL BIRTH	NO	NO	FEMALE	9	9	2855	NO	NO	NO	NO	YES	NO	NO	NO	NO	NO	11,57	NO	YES	YES
VAGINAL BIRTH	NO	NO	MALE	6	8	3275	NO	NO	NO	NO	YES	NO	NO	NO	NO	YES	11,57	NO	NO	YES
VAGINAL BIRTH	NO	YES	MALE	6	8	2910	NO	NO	NO	NO	YES	NO	NO	NO	NO	NO	11,57	NO	NO	YES
VAGINAL BIRTH	NO	NO	MALE	8	9	2975	NO	NO	NO	NO	YES	NO	NO	NO	NO	NO	11,57	NO	NO	YES
VAGINAL BIRTH	NO	NO	FEMALE	8	9	3740	NO	NO	NO	NO	YES	NO	NO	NO	NO	NO	11,57	NO	NO	YES
VAGINAL BIRTH	NO	NO	FEMALE	9	10	3130	NO	NO	NO	NO	YES	NO	NO	NO	NO	NO	11,57	NO	NO	YES
VAGINAL BIRTH	NO	NO	MALE	9	9	2905	NO	NO	NO	NO	YES	NO	NO	NO	NO	NO	11,57	NO	NO	YES
VAGINAL BIRTH	YES	NO	FEMALE	7	9	1825	YES	YES	NO	NO	YES	NO	NO	NO	YES	NO	11,71	NO	NO	NO
VAGINAL BIRTH	NO	NO	FEMALE	9	9	3610	NO	NO	NO	NO	YES	NO	NO	NO	NO	NO	11,71	NO	NO	NO
VAGINAL BIRTH	YES	NO	MALE	10	10	2980	NO	YES	NO	NO	YES	NO	NO	NO	NO	NO	11,71	NO	YES	NO
VAGINAL BIRTH	YES	NO	MALE	6	9	2680	NO	NO	NO	NO	YES	NO	NO	NO	NO	YES	11,71	NO	NO	NO
C-SECTIONYES	NO	MALE	8	9	1975	NO	YES	NO	NO	YES	NO	NO	NO	NO	NO	11,71	NO	YES	NO	
VAGINAL BIRTH	YES	NO	FEMALE	9	10	2680	NO	NO	NO	NO	YES	NO	NO	NO	NO	NO	11,71	NO	YES	NO
VAGINAL BIRTH	NO	NO	MALE	9	10	3330	NO	NO	NO	NO	YES	NO	NO	NO	NO	NO	11,71	NO	YES	NO
VAGINAL BIRTH	YES	NO	FEMALE	2	9	2810	NO	YES	NO	YES	YES	YES	NO	NO	NO	YES	11,71	NO	YES	NO
VAGINAL BIRTH	YES	NO	FEMALE	9	9	2600	YES	YES	NO	YES	YES	YES	NO	NO	NO	NO	11,71	NO	YES	NO
C-SECTIONNO	NO	MALE	8	9	2580	NO	NO	NO	NO	YES	NO	NO	NO	NO	NO	11,71	NO	NO	NO	
C-SECTIONYES	NO	FEMALE	9	9	2630	NO	NO	NO	NO	YES	NO	NO	NO	NO	NO	11,71	NO	NO	NO	
C-SECTIONNO	NO	FEMALE	9	10	3500	NO	NO	NO	NO	YES	NO	NO	NO	NO	NO	11,71	NO	NO	NO	
C-SECTIONYES	NO	MALE	8	9	3955	YES	YES	NO	NO	YES	NO	NO	NO	NO	NO	11,71	NO	NO	NO	
C-SECTIONNO	NO	FEMALE	8	9	3235	NO	NO	NO	NO	YES	NO	NO	NO	NO	NO	11,71	NO	NO	NO	
C-SECTIONNO	NO	MALE	8	9	2415	NO	NO	NO	NO	YES	NO	NO	NO	NO	NO	11,71	NO	NO	YES	
VAGINAL BIRTH	NO	NO	FEMALE	3	7	3110	NO	NO	NO	NO	YES	NO	NO	NO	NO	YES	11,71	NO	YES	YES
C-SECTIONNO	NO	FEMALE	8	9	3990	NO	NO	NO	NO	YES	NO	NO	NO	NO	NO	11,71	NO	NO	YES	
VAGINAL BIRTH	NO	NO	MALE	9	10	3560	NO	NO	NO	NO	YES	NO	NO	NO	NO	NO	11,71	NO	NO	YES
VAGINAL BIRTH	NO	NO	MALE	8	9	2785	NO	NO	NO	NO	YES	NO	NO	NO	NO	NO	11,71	NO	NO	YES
C-SECTIONYES	NO	MALE	9	9	3485	NO	YES	NO	NO	YES	NO	NO	NO	NO	NO	11,86	NO	NO	NO	
VAGINAL BIRTH	YES	NO	FEMALE	8	9	3360	NO	NO	NO	NO	YES	NO	NO	NO	NO	NO	11,86	NO	YES	NO
C-SECTIONYES	NO	FEMALE	7	8	3645	NO	YES	NO	NO	YES	NO	NO	NO	NO	NO	11,86	NO	NO	NO	
VAGINAL BIRTH	NO	NO	FEMALE	8	9	2905	NO	NO	NO	NO	YES	NO	NO	NO	NO	NO	11,86	NO	YES	NO
VAGINAL BIRTH	NO	NO	MALE	9	10	3005	NO	NO	NO	NO	YES	NO	NO	NO	NO	NO	11,86	NO	NO	NO
VAGINAL BIRTH	YES	NO	MALE	8	9	2680	NO	NO	NO	NO	YES	NO	NO	NO	NO	NO	11,86	NO	NO	NO
VAGINAL BIRTH	YES	NO	MALE	9	10	2090	YES	NO	NO	NO	YES	NO	NO	NO	YES	NO	11,86	NO	NO	NO
C-SECTIONNO	NO	FEMALE	9	9	3160	NO	NO	NO	NO	YES	NO	NO	NO	NO	NO	11,86	NO	NO	NO	
C-SECTIONYES	NO	FEMALE	8	9	2725	NO	YES	NO	NO	YES	NO	NO	NO	NO	NO	11,86	NO	NO	NO	
C-SECTIONYES	NO	FEMALE	3	8	2890	NO	NO	NO	NO	YES	NO	NO	NO	NO	YES	11,86	NO	NO	NO	
VAGINAL BIRTH	YES	NO	FEMALE	9	9	3450	NO	NO	NO	NO	YES	NO	NO	NO	NO	NO	11,86	NO	NO	YES
VAGINAL BIRTH	NO	NO	FEMALE	9	10	3385	NO	NO	NO	NO	YES	NO	NO	NO	NO	NO	11,86	NO	YES	YES
C-SECTIONNO	NO	FEMALE	9	9	3150	NO	NO	NO	NO	YES	NO	NO	NO	NO	NO	11,86	NO	NO	YES	
VAGINAL BIRTH	NO	NO	MALE	10	10	2860	NO	NO	NO	NO	YES	NO	NO	NO	NO	NO	11,86	NO	YES	YES
VAGINAL BIRTH	NO	NO	MALE	8	9	3415	NO	NO	NO	NO	YES	NO	NO	NO	NO	NO	11,86	NO	YES	YES
VAGINAL BIRTH	NO	NO	MALE	9	9	3430	NO	NO	NO	NO	YES	NO	NO	NO	NO	NO	11,86	NO	YES	YES
VAGINAL BIRTH	NO	NO	FEMALE	8	9	3145	NO	NO	NO	NO	YES	NO	NO	NO	NO	NO	11,86	NO	NO	YES
VAGINAL BIRTH	NO	YES	MALE	9	10	3000	NO	NO	NO	NO	YES	NO	NO	NO	NO	NO	11,86	NO	NO	YES
VAGINAL BIRTH	NO	NO	MALE	9	10	3430	NO	NO	NO	NO	YES	NO	NO	NO	NO	NO	11,86	NO	NO	YES
VAGINAL BIRTH	NO	NO	MALE	9	10	3070	NO	NO	NO	NO	YES	NO	NO	NO	NO	NO	11,86	NO	NO	YES
VAGINAL BIRTH	NO	NO	FEMALE	9	10	2815	NO	NO	NO	NO	YES	NO	NO	NO	NO	NO	11,86	NO	NO	YES
VAGINAL BIRTH	YES	NO	MALE	6	9	1955	YES	NO	NO	NO	YES	NO	NO	NO	YES	YES	12,00	NO	NO	NO
VAGINAL BIRTH	YES	NO	FEMALE			2519		NO	NO	NO	YES	NO	NO	YES	NO	NO	12,00	NO	NO	NO
C-SECTIONNO	NO	MALE	7	10	2885	NO	NO	NO	NO	YES	NO	NO	NO	NO	NO	12,00	NO	NO	NO	

C-SECTION	NO	NO	FEMALE	8	9	3175	NO	NO	NO	NO	YES	NO	NO	NO	NO	NO	12,00	NO	NO	NO	
VAGINAL BIRTH		NO	NO	MALE	8	9	2985	NO	NO	NO	NO	YES	NO	NO	NO	NO	NO	12,00	NO	YES	NO
C-SECTION	NO	NO	FEMALE	8	9	3815	NO	NO	NO	NO	YES	NO	NO	NO	NO	NO	12,00	NO	YES	NO	
C-SECTION	NO	NO	MALE	9	10	3460	NO	NO	NO	NO	YES	NO	NO	NO	NO	NO	12,00	NO	YES	NO	
VAGINAL BIRTH		NO	NO	FEMALE	8	9	2640	NO	NO	NO	NO	YES	NO	NO	NO	NO	NO	12,00	NO	NO	NO
VAGINAL BIRTH		NO	NO	FEMALE	9	10	3020	NO	NO	NO	NO	YES	NO	NO	NO	NO	NO	12,00	NO	NO	NO
VAGINAL BIRTH		NO	NO	FEMALE	9	9	3380	NO	NO	NO	NO	YES	NO	NO	NO	NO	NO	12,00	NO	NO	NO
C-SECTION	YES	NO	MALE	8	9	1680	YES	NO	NO	YES	YES	NO	NO	NO	NO	NO	12,00	NO	NO	NO	
C-SECTION	YES	NO	FEMALE	8	9	1755	YES	NO	NO	YES	YES	NO	NO	NO	NO	NO	12,00	NO	NO	NO	
C-SECTION	YES	NO	MALE	8	9	2335	YES	NO	NO	YES	YES	NO	NO	NO	NO	NO	12,00	NO	NO	NO	
C-SECTION	YES	NO	MALE	8	10	3265	NO	YES	NO	NO	YES	NO	NO	NO	NO	NO	12,00	NO	NO	NO	
C-SECTION	NO	NO	MALE	9	9	2715	NO	NO	NO	NO	YES	NO	NO	NO	NO	NO	12,00	NO	NO	NO	
C-SECTION	YES	NO	FEMALE	8	9	3070	NO	YES	NO	NO	YES	NO	NO	NO	NO	NO	12,00	NO	NO	NO	
C-SECTION	YES	NO	FEMALE	8	9	2660	NO	NO	NO	NO	YES	NO	NO	NO	NO	NO	12,00	NO	NO	NO	
C-SECTION	YES	NO	MALE	9	10	1795	YES	NO	NO	NO	YES	NO	NO	NO	YES	NO	12,00	NO	NO	NO	
C-SECTION	YES	NO	FEMALE	9	9	3245	NO	YES	NO	NO	YES	NO	NO	NO	NO	NO	12,00	NO	NO	NO	
C-SECTION	YES	NO	FEMALE	8	10	2835	NO	NO	NO	NO	YES	NO	NO	NO	NO	NO	12,00	NO	NO	NO	
C-SECTION	YES	NO	MALE	8	9	3475	NO	NO	YES	NO	YES	NO	NO	NO	YES	NO	12,00	NO	NO	NO	
C-SECTION	YES	NO	MALE	9	9	3665	NO	YES	NO	NO	YES	NO	NO	NO	NO	NO	12,00	NO	NO	NO	
C-SECTION	NO	NO	MALE	9	10	3305	NO	NO	NO	NO	YES	NO	NO	NO	NO	NO	12,00	NO	NO	NO	
C-SECTION	NO	NO	FEMALE	9	10	3335	NO	NO	NO	NO	YES	NO	NO	NO	NO	NO	12,00	NO	NO	NO	
VAGINAL BIRTH		YES	NO	FEMALE	9	9	2410	YES	NO	NO	NO	YES	NO	NO	NO	NO	NO	12,00	NO	NO	YES
VAGINAL BIRTH		NO	NO	FEMALE	9	9	2325	NO	NO	NO	NO	YES	NO	NO	NO	NO	NO	12,00	NO	NO	YES
VAGINAL BIRTH		NO	NO	FEMALE	9	9	2300	NO	NO	NO	NO	YES	NO	NO	NO	NO	NO	12,00	NO	NO	YES
VAGINAL BIRTH		NO	NO	FEMALE	9	10	3000	NO	NO	NO	NO	YES	NO	NO	NO	NO	NO	12,00	NO	NO	YES
VAGINAL BIRTH		NO	NO	MALE	9	10	3490	NO	NO	NO	NO	YES	NO	NO	NO	NO	NO	12,00	NO	NO	YES
VAGINAL BIRTH		YES	NO	FEMALE	9	10	2880	NO	NO	NO	NO	YES	NO	NO	NO	NO	NO	12,00	NO	NO	YES
VAGINAL BIRTH		YES	NO	MALE	9	9	3655	NO	NO	NO	NO	YES	NO	NO	NO	NO	NO	12,00	NO	YES	YES
VAGINAL BIRTH		NO	NO	MALE	9	10	2745	NO	NO	NO	NO	YES	NO	NO	NO	NO	NO	12,00	NO	NO	YES
VAGINAL BIRTH		NO	NO	MALE	9	9	3060	NO	NO	NO	NO	YES	NO	NO	NO	NO	NO	12,00	NO	NO	YES
C-SECTION	NO	NO	FEMALE	9	10	3560	NO	NO	NO	NO	YES	NO	NO	NO	NO	NO	12,00	NO	YES	YES	
VAGINAL BIRTH		NO	NO	MALE	9	10	3650	NO	NO	NO	NO	YES	NO	NO	NO	NO	NO	12,00	NO	NO	YES
VAGINAL BIRTH		YES	NO	MALE	8	9	2755	NO	YES	NO	NO	YES	NO	NO	NO	NO	NO	12,00	NO	YES	YES
VAGINAL BIRTH		YES	NO	MALE	8	9	2640	NO	YES	NO	NO	YES	NO	NO	NO	YES	NO	12,14	NO	YES	NO
VAGINAL BIRTH		YES	NO	MALE	8	9	3225	NO	NO	YES	NO	YES			NO	NO	NO	12,14	NO	NO	NO
VAGINAL BIRTH		NO	NO	MALE	9	9	2885	NO	NO	NO	NO	YES	NO	NO	NO	NO	NO	12,14	NO	NO	NO
VAGINAL BIRTH		NO	NO	MALE	9	9	3135	NO	NO	NO	NO	YES	NO	NO	NO	NO	NO	12,14	NO	NO	NO
VAGINAL BIRTH		YES	NO	FEMALE	9	9	3230	NO	YES	NO	NO	YES	NO	NO	NO	NO	NO	12,14	NO	YES	NO
C-SECTION	YES	NO	MALE	4	9	3365	NO	NO	NO	NO	YES	NO	NO	NO	NO	YES	12,14	NO	NO	NO	
C-SECTION	NO	NO	FEMALE	8	9	2945	NO	NO	NO	NO	YES	NO	NO	NO	NO	NO	12,14	NO	NO	NO	
C-SECTION	NO	NO	MALE	8	9	3005	NO	NO	NO	NO	YES	NO	NO	NO	NO	NO	12,14	NO	NO	NO	
C-SECTION	YES	NO	FEMALE	8	9	2750	NO	NO	NO	NO	YES	NO	NO	NO	NO	NO	12,14	NO	NO	NO	
C-SECTION	YES	NO	MALE	7	9	3935	NO	YES	NO	NO	YES	NO	NO	NO	NO	NO	12,14	NO	NO	NO	
C-SECTION	YES	NO	MALE	5	9	2110	NO	NO	NO	YES	YES	NO	NO	NO	NO	YES	12,14	NO	NO	YES	
C-SECTION	YES	NO	MALE	6	9	1995	NO	NO	NO	YES	YES	NO	NO	NO	NO	YES	12,14	NO	NO	YES	
VAGINAL BIRTH		NO	NO	FEMALE	9	9	3245	NO	NO	NO	NO	YES	NO	NO	NO	NO	NO	12,14	NO	NO	YES
VAGINAL BIRTH		NO	YES	FEMALE	8	10	3445	NO	NO	NO	NO	YES	NO	NO	NO	NO	NO	12,14	NO	YES	YES
VAGINAL BIRTH		NO	NO	FEMALE	9	10	2400	NO	NO	NO	NO	YES	NO	NO	NO	NO	NO	12,14	NO	NO	YES
VAGINAL BIRTH		YES	YES	MALE	8	9	3595	NO	YES	NO	NO	YES	NO	NO	NO	NO	NO	12,14	NO	YES	YES
VAGINAL BIRTH		YES	NO	MALE	8	9	2845	NO	YES	NO	NO	YES	NO	NO	NO	NO	NO	12,14	NO	NO	YES
C-SECTION	NO	NO	MALE	8	9	3240	NO	NO	NO	NO	YES	NO	NO	NO	NO	NO	12,14	NO	NO	YES	
VAGINAL BIRTH		NO	NO	FEMALE	10	10	3275	NO	NO	NO	NO	YES	NO	NO	NO	NO	NO	12,14	NO	NO	YES
VAGINAL BIRTH		NO	NO	FEMALE	8	9	3185	NO	NO	NO	NO	YES	NO	NO	NO	NO	NO	12,14	NO	NO	YES

VAGINAL BIRTH	YES	YES	FEMALE	9	9	2905	NO	NO	NO	NO	YES	NO	NO	NO	NO	NO	12,14	NO	YES	YES
VAGINAL BIRTH	NO	NO	FEMALE	9	9	3180	NO	NO	NO	NO	YES	NO	NO	NO	NO	NO	12,14	NO	NO	YES
C-SECTIONYES	NO	MALE	9	10	2755	NO	YES	NO	NO	YES	NO	YES	NO	NO	NO	12,29	NO	NO	NO	
C-SECTIONYES	NO	FEMALE	8	9	2985	NO	NO	NO	NO	YES	NO	NO	NO	NO	NO	12,29	NO	NO	NO	
C-SECTIONYES	NO	MALE	9	10	2980	NO	YES	NO	NO	YES	NO	NO	NO	NO	NO	12,29	NO	NO	NO	
VAGINAL BIRTH	NO	NO	FEMALE	8	9	4000	NO	NO	NO	NO	YES	NO	NO	NO	NO	NO	12,29	NO	YES	NO
VAGINAL BIRTH	NO	NO	FEMALE	9	10	3445	NO	NO	NO	NO	YES	NO	NO	NO	NO	NO	12,29	NO	NO	NO
VAGINAL BIRTH	NO	NO	FEMALE	9	10	2895	NO	NO	NO	NO	YES	NO	NO	NO	NO	NO	12,29	NO	NO	NO
C-SECTIONNO	NO	MALE	7	9	3795	NO	NO	NO	NO	YES	NO	NO	NO	NO	NO	12,29	NO	NO	NO	
C-SECTIONNO	NO	MALE	9	10	4645	NO	NO	NO	NO	YES	NO	NO	NO	NO	NO	12,29	NO	NO	NO	
C-SECTIONYES	NO	MALE	7	9	3990	YES	NO	NO	NO	YES	NO	NO	NO	NO	YES	12,29	NO	NO	NO	
C-SECTIONNO	NO	MALE	9	10	3490	NO	NO	NO	NO	YES	NO	NO	NO	NO	NO	12,29	NO	NO	NO	
C-SECTIONNO	NO	FEMALE	9	9	3540	NO	NO	NO	NO	YES	NO	NO	NO	NO	NO	12,29	NO	NO	NO	
VAGINAL BIRTH	NO	NO	FEMALE	9	9	2725	NO	NO	NO	NO	YES	NO	NO	NO	NO	NO	12,29	NO	NO	YES
VAGINAL BIRTH	YES	NO	FEMALE	8	10	2785	NO	NO	NO	NO	YES	NO	NO	NO	YES	NO	12,29	NO	NO	YES
C-SECTIONYES	NO	MALE	9	10	2355	NO	YES	NO	NO	YES	NO	NO	NO	NO	NO	12,29	NO	NO	YES	
VAGINAL BIRTH	YES	NO	FEMALE	9	10	3170	NO	NO	NO	NO	YES	NO	NO	NO	NO	NO	12,29	NO	NO	YES
VAGINAL BIRTH	NO	NO	MALE	9	10	3310	NO	NO	NO	NO	YES	NO	NO	NO	NO	NO	12,29	NO	YES	YES
VAGINAL BIRTH	NO	NO	FEMALE	9	10	2670	NO	NO	NO	NO	YES	NO	NO	NO	NO	NO	12,29	NO	NO	YES
VAGINAL BIRTH	NO	NO	FEMALE	9	9	2780	NO	NO	NO	NO	YES	NO	NO	NO	NO	NO	12,29	NO	NO	YES
VAGINAL BIRTH	NO	NO	FEMALE	8	9	3740	NO	NO	NO	NO	YES	NO	NO	NO	NO	NO	12,29	NO	NO	YES
VAGINAL BIRTH	NO	NO	FEMALE	8	9	2720	NO	NO	NO	NO	YES	NO	NO	NO	NO	NO	12,43	NO	YES	NO
C-SECTIONYES	NO	MALE	8	9	3790	NO	NO	YES	NO	YES	NO	NO	NO	NO	NO	12,43	NO	NO	NO	
VAGINAL BIRTH	YES	NO	FEMALE	9	10	3100	NO	NO	NO	NO	YES	NO	NO	NO	YES	NO	12,43	NO	YES	NO
VAGINAL BIRTH	NO	NO	MALE	8	9	3760	NO	NO	NO	NO	YES	NO	NO	NO	NO	NO	12,43	NO	NO	NO
C-SECTIONYES	NO	MALE	8	10	3735	NO	YES	NO	NO	YES	NO	NO	NO	NO	NO	12,43	NO	YES	NO	
VAGINAL BIRTH	NO	NO	FEMALE	5	9	2795	NO	NO	NO	NO	YES	NO	NO	NO	NO	YES	12,43	NO	NO	NO
C-SECTIONNO	NO	FEMALE	9	9	2450	NO	NO	NO	NO	YES	NO	NO	NO	NO	NO	12,43	NO	NO	NO	
C-SECTIONNO	NO	MALE	7	9	3760	NO	NO	NO	NO	YES	NO	NO	NO	NO	NO	12,43	NO	NO	NO	
C-SECTIONNO	NO	MALE	10	10	3035	NO	NO	NO	NO	YES	NO	NO	NO	NO	NO	12,43	NO	NO	NO	
C-SECTIONYES	NO	UNDETERMINATED		8	9	2500	NO	NO	NO	NO	YES	YES	YES	NO	NO	NO	12,43	NO	NO	NO
VAGINAL BIRTH	NO	NO	FEMALE	9	9	3090	NO	NO	NO	NO	YES	NO	NO	NO	NO	NO	12,43	NO	NO	YES
C-SECTIONNO	NO	MALE	8	10	2955	NO	NO	NO	NO	YES	NO	NO	NO	NO	NO	12,43	NO	NO	YES	
C-SECTIONNO	NO	MALE	10	10	4035	NO	NO	NO	NO	YES	NO	NO	NO	NO	NO	12,43	NO	YES	YES	
C-SECTIONNO	NO	MALE	8	10	3205	NO	NO	NO	NO	YES	NO	NO	NO	NO	NO	12,43	NO	NO	YES	
VAGINAL BIRTH	NO	NO	MALE	9	10	2965	NO	NO	NO	NO	YES	NO	NO	NO	NO	NO	12,43	NO	YES	YES
C-SECTIONYES	NO	FEMALE	7	9	1750	YES	NO	YES	NO	YES	NO	NO	NO	NO	NO	12,43	NO	NO	YES	
VAGINAL BIRTH	YES	NO	FEMALE	9	10	2890	NO	NO	NO	NO	YES	NO	NO	NO	NO	NO	12,43	NO	NO	YES
VAGINAL BIRTH	NO	NO	FEMALE	9	9	2820	NO	NO	NO	NO	YES	NO	NO	NO	NO	NO	12,57	NO	NO	NO
VAGINAL BIRTH	YES	NO	FEMALE	9	10	3535	NO	YES	NO	NO	YES	NO	NO	NO	NO	NO	12,57	NO	YES	NO
VAGINAL BIRTH	NO	NO	FEMALE	9	9	2860	NO	NO	NO	NO	YES	NO	NO	NO	NO	NO	12,57	NO	YES	NO
VAGINAL BIRTH	NO	NO	MALE	6	8	2530	NO	NO	NO	NO	YES	NO	NO	NO	NO	YES	12,57	NO	YES	NO
C-SECTIONYES	NO	MALE	2	4	840	YES	NO	YES	NO	YES	NO	NO	NO	NO	YES	12,57	NO	NO	NO	
C-SECTIONYES	NO	FEMALE	4	8	710	YES	YES	NO	NO	YES	NO	YES	NO	NO	YES	12,57	NO	NO	NO	
VAGINAL BIRTH	YES	NO	FEMALE	8	9	2590	NO	YES	NO	NO	YES	NO	NO	NO	NO	NO	12,57	NO	YES	NO
C-SECTIONYES	NO	MALE	7	9	2990	NO	YES	NO	NO	YES	NO	NO	NO	NO	NO	12,57	NO	NO	NO	
C-SECTIONNO	NO	FEMALE	8	8	3210	NO	NO	NO	NO	YES	NO	NO	NO	NO	NO	12,57	NO	NO	NO	
VAGINAL BIRTH	NO	NO	MALE	2	9	3960	NO	NO	NO	NO	YES	NO	NO	NO	NO	YES	12,57	NO	YES	NO
C-SECTIONNO	NO	FEMALE	9	10	3205	NO	NO	NO	NO	YES	NO	NO	NO	NO	NO	12,57	NO	NO	NO	
VAGINAL BIRTH	NO	NO	MALE	9	10	3075	NO	NO	NO	NO	YES	NO	NO	NO	NO	NO	12,57	NO	NO	YES
VAGINAL BIRTH	NO	NO	MALE	9	10	2800	NO	NO	NO	NO	YES	NO	NO	NO	NO	NO	12,57	NO	NO	YES
VAGINAL BIRTH	NO	NO	MALE	9	9	3530	NO	NO	NO	NO	YES	NO	NO	NO	NO	NO	12,57	NO	YES	YES
VAGINAL BIRTH	NO	NO	FEMALE	10	10	2905	NO	NO	NO	NO	YES	NO	NO	NO	NO	NO	12,57	NO	YES	YES

VAGINAL BIRTH	NO	NO	MALE	8	9	3860	NO	NO	NO	NO	YES	NO	NO	NO	NO	NO	12,57	NO	NO	YES
VAGINAL BIRTH	YES	NO	MALE	9	10	3680	NO	NO	YES	NO	YES	NO	NO	NO	NO	NO	12,57	NO	NO	YES
C-SECTIONNO	NO	FEMALE	9	10	3475	NO	NO	NO	NO	YES	NO	NO	NO	NO	NO	12,57	NO	NO	YES	
VAGINAL BIRTH	NO	NO	MALE	8	9	3140	NO	NO	NO	NO	YES	NO	NO	NO	NO	NO	12,57	NO	NO	YES
VAGINAL BIRTH	NO	NO	MALE	3	8	3965	YES	NO	NO	NO	YES	NO	NO	NO	NO	YES	12,71	NO	YES	NO
VAGINAL BIRTH	YES	NO	FEMALE	9	10	2530	NO	NO	YES	NO	YES	NO	NO	NO	NO	NO	12,71	NO	NO	NO
C-SECTIONYES	NO	MALE	9	9	2875	NO	NO	NO	YES	YES	NO	NO	NO	NO	NO	12,71	NO	NO	NO	
C-SECTIONYES	NO	FEMALE	9	10	2505	NO	NO	NO	YES	YES	NO	NO	NO	NO	NO	12,71	NO	NO	NO	
C-SECTIONYES	NO	MALE	8	8	1780	YES	YES	NO	NO	YES	NO	NO	NO	NO	NO	12,71	NO	NO	NO	
C-SECTIONYES	NO	MALE	9	10	3310	NO	YES	NO	NO	YES	NO	NO	NO	NO	NO	12,71	NO	NO	NO	
C-SECTIONYES	NO	MALE	5	9	3385	NO	NO	NO	NO	YES	NO	NO	NO	NO	NO	12,71	NO	NO	NO	
C-SECTIONNO	NO	FEMALE	9	9	2810	NO	NO	NO	NO	YES	NO	NO	NO	NO	NO	12,71	NO	NO	NO	
VAGINAL BIRTH	NO	NO	MALE	8	9	2885	NO	NO	NO	NO	YES	NO	NO	NO	NO	NO	12,71	NO	NO	YES
VAGINAL BIRTH	NO	NO	FEMALE	9	9	2970	NO	NO	NO	NO	YES	NO	NO	NO	NO	NO	12,71	NO	NO	YES
VAGINAL BIRTH	YES	NO	FEMALE	7	9	3585	NO	NO	NO	NO	YES	NO	NO	NO	YES	NO	12,71	NO	YES	YES
VAGINAL BIRTH	YES	NO	FEMALE	8	9	3560	NO	NO	NO	NO	YES	NO	NO	NO	NO	NO	12,71	NO	NO	YES
VAGINAL BIRTH	YES	NO	MALE	8	8	3915	NO	YES	NO	NO	YES	NO	NO	NO	NO	NO	12,71	NO	NO	YES
VAGINAL BIRTH	NO	NO	MALE	8	9	3150	NO	NO	NO	NO	YES	NO	NO	NO	NO	NO	12,71	NO	NO	YES
VAGINAL BIRTH	NO	NO	FEMALE	7	9	3850	NO	NO	NO	NO	YES	NO	NO	NO	NO	NO	12,71	NO	NO	YES
VAGINAL BIRTH	YES	NO	FEMALE	8	9	2495	NO	NO	NO	NO	YES	NO	NO	NO	NO	NO	12,71	NO	NO	YES
VAGINAL BIRTH	NO	NO	MALE	8	9	2950	NO	NO	NO	NO	YES	NO	NO	NO	NO	NO	12,71	NO	NO	YES
VAGINAL BIRTH	YES	NO	MALE	10	10	3070	NO	YES	NO	NO	YES	NO	NO	NO	NO	NO	12,71	NO	NO	YES
C-SECTIONNO	NO	MALE	2	2	3115	YES	NO	NO	NO	YES	NO	NO	YES	NO	YES	12,71	NO	NO	YES	
VAGINAL BIRTH	YES	NO	FEMALE	9	10	3240	NO	NO	YES	NO	YES	NO	NO	NO	NO	NO	12,86	NO	YES	NO
VAGINAL BIRTH	YES	NO	MALE	8	9	2810	NO	YES	NO	NO	YES	NO	NO	NO	NO	NO	12,86	NO	NO	NO
VAGINAL BIRTH	YES	NO	MALE	9	10	3815	NO	YES	NO	NO	YES	NO	NO	NO	NO	NO	12,86	NO	NO	NO
VAGINAL BIRTH	NO	NO	FEMALE	8	9	3385	NO	NO	NO	NO	YES	NO	NO	NO	NO	NO	12,86	NO	NO	NO
VAGINAL BIRTH	YES	NO	MALE	5	9	2475	YES	NO	NO	NO	YES	NO	NO	NO	NO	YES	12,86	NO	NO	NO
C-SECTIONYES	NO	MALE	7	8	3360	NO	YES	YES	NO	YES	NO	NO	NO	NO	NO	12,86	NO	NO	NO	
C-SECTIONYES	NO	MALE	2	5	1975	YES	NO	NO	NO	YES	NO	NO	NO	NO	NO	12,86	NO	NO	NO	
C-SECTIONYES	NO	MALE	8	9	1375	YES	YES	NO	NO	YES	NO	NO	NO	NO	NO	12,86	NO	NO	NO	
C-SECTIONYES	NO	MALE	9	9	1780	YES	YES	NO	NO	YES	NO	NO	NO	NO	NO	12,86	NO	NO	NO	
C-SECTIONYES	NO	FEMALE	9	9	2530	NO	NO	YES	NO	YES	NO	NO	NO	NO	NO	12,86	NO	NO	NO	
C-SECTIONYES	NO	FEMALE	9	9	3370	YES	NO	NO	NO	YES	YES	NO	NO	NO	NO	12,86	NO	NO	NO	
C-SECTIONNO	NO	FEMALE	8	9	3020	YES	NO	NO	NO	YES	NO	NO	NO	NO	NO	12,86	NO	YES	NO	
C-SECTIONNO	NO	MALE	9	9	3505	NO	NO	NO	NO	YES	NO	NO	NO	NO	NO	12,86	NO	NO	NO	
C-SECTIONNO	NO	MALE	9	9	3615	NO	NO	NO	NO	YES	NO	NO	NO	NO	NO	12,86	NO	NO	NO	
VAGINAL BIRTH	YES	NO	FEMALE	2	2	3770		NO	NO	NO	YES	YES	YES	NO	NO	NO	12,86	NO	YES	YES
C-SECTIONYES	NO	FEMALE	9	9	1550	YES	NO	NO	NO	YES	NO	NO	NO	NO	NO	12,86	NO	NO	YES	
VAGINAL BIRTH	NO	NO	MALE	9	9	3485	NO	NO	NO	NO	YES	NO	NO	NO	NO	NO	12,86	NO	NO	YES
VAGINAL BIRTH	NO	NO	FEMALE	8	9	2790	NO	NO	NO	NO	YES	NO	NO	NO	NO	NO	12,86	NO	NO	YES
C-SECTIONNO	NO	FEMALE	8	10	3200	NO	NO	NO	NO	YES	NO	NO	NO	NO	NO	12,86	NO	NO	YES	
VAGINAL BIRTH	NO	NO	MALE	9	9	3005	NO	NO	NO	NO	YES	NO	NO	NO	NO	NO	12,86	NO	YES	YES
VAGINAL BIRTH	NO	NO	MALE	7	9	3650	NO	NO	NO	NO	YES	NO	NO	NO	NO	NO	12,86	NO	NO	YES
VAGINAL BIRTH	NO	NO	FEMALE	9	9	3175	NO	NO	NO	NO	YES	NO	NO	NO	NO	NO	12,86	NO	NO	YES
VAGINAL BIRTH	NO	NO	FEMALE	9	9	3630	NO	NO	NO	NO	YES	NO	NO	NO	NO	NO	12,86	NO	NO	YES
VAGINAL BIRTH	NO	NO	FEMALE	9	9	3545	NO	NO	NO	NO	YES	NO	NO	NO	NO	NO	12,86	NO	NO	YES
VAGINAL BIRTH	NO	NO	FEMALE	9	10	2725	NO	NO	NO	NO	YES	NO	NO	NO	NO	NO	12,86	NO	NO	YES
C-SECTIONYES	NO	MALE	8	9	2920	NO	YES	NO	NO	YES	NO	YES	NO	NO	NO	13,00	NO	YES	NO	
VAGINAL BIRTH	NO	NO	FEMALE	9	10	3345	YES	NO	NO	NO	YES	NO	NO	NO	NO	NO	13,00	NO	NO	NO
VAGINAL BIRTH	YES	NO	MALE	9	10	2375	NO	NO	NO	NO	YES	NO	NO	NO	NO	NO	13,00	NO	NO	NO
C-SECTIONNO	NO	FEMALE	7	9	3355	NO	NO	NO	NO	YES	NO	NO	NO	NO	NO	13,00	NO	YES	NO	
VAGINAL BIRTH	YES	NO	MALE	8	9	3055	NO	NO	NO	NO	YES	NO	NO	NO	YES	NO	13,00	NO	NO	NO

VAGINAL BIRTH	YES	NO	FEMALE	9	10	3000	NO	YES	NO	NO	YES	NO	NO	NO	NO	NO	13,00	NO	NO	NO
VAGINAL BIRTH	NO	NO	MALE	9	10	3860	NO	NO	NO	NO	YES	NO	NO	NO	NO	NO	13,00	NO	NO	NO
VAGINAL BIRTH	NO	NO	MALE	8	9	2905	NO	NO	NO	NO	YES	NO	NO	NO	NO	NO	13,00	NO	NO	NO
VAGINAL BIRTH	YES	NO	MALE	9	10	2630	NO	YES	NO	NO	YES	NO	NO	NO	NO	NO	13,00	NO	NO	NO
C-SECTIONNO	NO	MALE	9	10	2935	NO	NO	NO	NO	YES	NO	NO	NO	NO	NO	13,00	NO	YES	NO	
VAGINAL BIRTH	YES	NO	MALE	8	10	2825	NO	YES	NO	NO	YES	NO	NO	NO	NO	NO	13,00	NO	YES	NO
C-SECTIONYES	NO	FEMALE	9	9	2010	YES	YES	YES	NO	YES	NO	NO	NO	NO	NO	13,00	NO	NO	NO	
C-SECTIONYES	NO	MALE	9	10	1730	YES	NO	NO	NO	YES	NO	NO	NO	YES	NO	13,00	NO	NO	NO	
C-SECTIONYES	NO	MALE	9	9	3185	NO	NO	NO	NO	YES	NO	NO	NO	NO	NO	13,00	NO	NO	NO	
C-SECTIONYES	NO	FEMALE	9	9	772	YES	YES	NO	NO	YES	NO	NO	YES	NO	NO	13,00	NO	NO	NO	
C-SECTIONNO	NO	FEMALE	9	10	3060	NO	NO	NO	NO	YES	NO	NO	NO	NO	NO	13,00	NO	NO	NO	
C-SECTIONYES	NO	MALE	8	9	3310	NO	NO	NO	NO	YES	NO	NO	NO	YES	NO	13,00	NO	NO	NO	
C-SECTIONNO	NO	FEMALE	8	10	3910	NO	NO	NO	NO	YES	NO	NO	NO	NO	NO	13,00	NO	NO	NO	
VAGINAL BIRTH	NO	YES	FEMALE	9	10	2500	NO	NO	NO	NO	YES	NO	YES	NO	NO	NO	13,00	NO	NO	YES
VAGINAL BIRTH	NO	NO	FEMALE	8	9	2895	NO	NO	NO	NO	YES	NO	NO	NO	NO	NO	13,00	NO	YES	YES
VAGINAL BIRTH	NO	YES	FEMALE	8	9	3765	NO	NO	NO	NO	YES	NO	NO	NO	NO	NO	13,00	NO	YES	YES
VAGINAL BIRTH	YES	YES	MALE	9	9	2930	NO	NO	NO	NO	YES	NO	NO	NO	NO	NO	13,00	NO	YES	YES
VAGINAL BIRTH	YES	NO	MALE	9	9	3285	NO	NO	NO	NO	YES	NO	NO	NO	NO	NO	13,00	NO	NO	YES
VAGINAL BIRTH	NO	NO	FEMALE	9	10	2860	NO	NO	NO	NO	YES	NO	NO	NO	NO	NO	13,00	NO	NO	YES
VAGINAL BIRTH	NO	NO	MALE	10	10	3205	NO	NO	NO	NO	YES	NO	NO	NO	NO	NO	13,00	NO	NO	YES
VAGINAL BIRTH	YES	NO	MALE	9	10	3260	NO	NO	NO	NO	YES	NO	NO	NO	NO	NO	13,00	NO	NO	YES
VAGINAL BIRTH	NO	NO	FEMALE	8	9	2920	NO	NO	NO	NO	YES	NO	NO	NO	NO	NO	13,00	NO	NO	YES
VAGINAL BIRTH	YES	NO	FEMALE	9	9	3250	NO	NO	NO	NO	YES	NO	NO	NO	NO	NO	13,00	NO	NO	YES
VAGINAL BIRTH	YES	NO	MALE	9	10	2470	NO	NO	NO	NO	YES	NO	NO	NO	YES	NO	13,14	NO	NO	NO
VAGINAL BIRTH	YES	NO	MALE	9	10	3505	YES	NO	NO	NO	YES	NO	NO	NO	NO	NO	13,14	NO	NO	NO
VAGINAL BIRTH	NO	NO	FEMALE	2	9	2295	NO	NO	NO	NO	YES	NO	NO	NO	NO	YES	13,14	NO	YES	NO
VAGINAL BIRTH	NO	NO	FEMALE	9	10	3455	NO	NO	NO	NO	YES	NO	NO	NO	NO	NO	13,14	NO	YES	NO
VAGINAL BIRTH	YES	NO	FEMALE	8	8	2505	NO	YES	NO	NO	YES	NO	NO	NO	NO	NO	13,14	NO	NO	NO
VAGINAL BIRTH	YES	NO	FEMALE	9	9	3185	NO	NO	NO	NO	YES	NO	NO	NO	NO	NO	13,14	NO	NO	NO
C-SECTIONYES	NO	FEMALE	9	10	2395	NO	YES	YES	NO	YES	NO	NO	NO	NO	NO	13,14	NO	NO	NO	
C-SECTIONYES	NO	FEMALE	8	9	3350	NO	NO	YES	NO	YES	NO	NO	NO	NO	NO	13,14	NO	NO	NO	
C-SECTIONNO	NO	MALE	6	9	3290	NO	NO	NO	NO	YES	NO	NO	NO	NO	YES	13,14	NO	NO	NO	
C-SECTIONYES	NO	FEMALE	2	4	3300	NO	YES	NO	NO	YES	NO	NO	NO	NO	YES	13,14	NO	NO	YES	
VAGINAL BIRTH	NO	NO	FEMALE	9	10	3160	NO	NO	NO	NO	YES	NO	NO	NO	NO	NO	13,14	NO	NO	YES
VAGINAL BIRTH	NO	NO	MALE	9	10	3195	NO	NO	NO	NO	YES	NO	NO	NO	NO	NO	13,14	NO	NO	YES
C-SECTIONNO	NO	FEMALE	9	10	2990	NO	NO	NO	NO	YES	NO	NO	NO	NO	NO	13,14	NO	NO	YES	
VAGINAL BIRTH	NO	NO	FEMALE	8	9	3245	NO	NO	NO	NO	YES	NO	NO	NO	NO	NO	13,14	NO	NO	YES
VAGINAL BIRTH	YES	NO	MALE	8	9	3285	NO	NO	YES	NO	YES	NO	NO	NO	NO	NO	13,14	NO	NO	YES
VAGINAL BIRTH	NO	NO	MALE	9	9	3225	NO	NO	NO	NO	YES	NO	NO	NO	NO	NO	13,14	NO	NO	YES
VAGINAL BIRTH	NO	NO	MALE	9	10	3375	NO	NO	NO	NO	YES	NO	NO	NO	NO	NO	13,29	NO	NO	NO
VAGINAL BIRTH	YES	NO	MALE	9	9	3630	NO	YES	NO	NO	YES	NO	NO	NO	NO	NO	13,29	NO	YES	NO
VAGINAL BIRTH	YES	NO	FEMALE	5	8	3750	NO	NO	YES	NO	YES	NO	NO	NO	NO	YES	13,29	NO	NO	NO
VAGINAL BIRTH	YES	NO	FEMALE	8	9	4045	YES	YES	YES	NO	YES	NO	NO	NO	NO	NO	13,29	NO	NO	NO
C-SECTIONYES	NO	FEMALE	9	9	3430	YES	YES	NO	NO	YES	YES	NO	NO	NO	NO	13,29	NO	NO	NO	
C-SECTIONYES	NO	MALE	9	9	3910	NO	NO	NO	NO	YES	NO	NO	NO	NO	NO	13,29	NO	NO	NO	
C-SECTIONYES	NO	MALE	9	10	3290	NO	YES	NO	NO	YES	NO	NO	NO	NO	NO	13,29	NO	NO	NO	
C-SECTIONYES	NO	MALE	9	9	3250	NO	NO	NO	NO	YES	NO	NO	NO	NO	NO	13,29	NO	NO	NO	
C-SECTIONYES	NO	FEMALE	9	10	2785	NO	NO	NO	NO	YES	NO	NO	NO	NO	NO	13,29	NO	NO	NO	
VAGINAL BIRTH	NO	NO	MALE	9	9	3410	NO	NO	NO	NO	YES	NO	NO	NO	NO	NO	13,29	NO	YES	YES
VAGINAL BIRTH	NO	NO	FEMALE	9	10	2810	NO	NO	NO	NO	YES	NO	NO	NO	NO	NO	13,29	NO	NO	YES
VAGINAL BIRTH	YES	NO	MALE			350		NO	NO	NO	YES	NO	NO	YES	NO	NO	13,29	NO	NO	YES
VAGINAL BIRTH	YES	NO	FEMALE	8	9	2405	NO	NO	NO	NO	YES	NO	NO	NO	YES	NO	13,29	NO	NO	YES
VAGINAL BIRTH	NO	NO	FEMALE	9	10	2750	NO	NO	NO	NO	YES	NO	NO	NO	NO	NO	13,29	NO	NO	YES

VAGINAL BIRTH	NO	NO	FEMALE	6	7	2580	NO	NO	NO	NO	YES	NO	NO	NO	NO	YES	13,29	NO	NO	YES
VAGINAL BIRTH	NO	NO	MALE	1	6	3195	YES	NO	NO	NO	YES	NO	NO	NO	NO	YES	13,29	NO	NO	YES
C-SECTIONNO	NO	MALE	2	7	3895	YES	NO	NO	NO	YES	YES	NO	NO	NO	YES	13,43	NO	NO	NO	
C-SECTIONYES	NO	FEMALE	9	10	2935	NO	YES	NO	NO	YES	NO	NO	NO	NO	NO	13,43	NO	NO	NO	
C-SECTIONNO	NO	FEMALE	6	8	4700	NO	NO	NO	NO	YES	NO	NO	NO	NO	YES	13,43	NO	NO	NO	
VAGINAL BIRTH	YES	NO	MALE	4	8	3120	YES	YES	NO	NO	YES	NO	NO	NO	NO	YES	13,43	NO	YES	NO
VAGINAL BIRTH	YES	NO	FEMALE	8	9	3615	NO	YES	NO	NO	YES	NO	NO	NO	NO	NO	13,43	NO	YES	NO
VAGINAL BIRTH	YES	NO	MALE	9	9	2725	NO	YES	YES	NO	YES	NO	NO	NO	NO	NO	13,43	NO	YES	NO
C-SECTIONYES	NO	MALE	4	9	2230	YES	NO	NO	YES	YES	NO	NO	NO	NO	YES	13,43	NO	NO	NO	
C-SECTIONYES	NO	MALE	8	9	2720	YES	NO	NO	YES	YES	NO	NO	NO	NO	NO	13,43	NO	NO	NO	
C-SECTIONYES	NO	MALE	8	9	4180	NO	NO	YES	NO	YES	NO	NO	NO	NO	NO	13,43	NO	NO	NO	
C-SECTIONNO	NO	FEMALE	9	10	3555	NO	NO	NO	NO	YES	NO	NO	NO	NO	NO	13,43	NO	NO	NO	
C-SECTIONYES	NO	MALE	9	9	3565	NO	NO	YES	NO	YES	NO	NO	NO	NO	NO	13,43	NO	NO	NO	
C-SECTIONNO	NO	FEMALE	8	9	2840	NO	NO	NO	NO	YES	NO	NO	NO	NO	NO	13,43	NO	NO	NO	
C-SECTIONYES	NO	FEMALE	9	10	2575	NO	NO	YES	NO	YES	NO	NO	NO	NO	NO	13,43	NO	NO	NO	
C-SECTIONYES	NO	FEMALE	8	9	2795	NO	NO	NO	NO	YES	NO	NO	NO	NO	NO	13,43	NO	NO	NO	
C-SECTIONYES	NO	MALE	8	9	3485	NO	YES	YES	NO	YES	NO	NO	NO	NO	NO	13,43	NO	NO	NO	
C-SECTIONYES	NO	FEMALE	9	9	3235	NO	NO	NO	NO	YES	NO	NO	NO	NO	NO	13,43	NO	NO	NO	
VAGINAL BIRTH	NO	NO	MALE	9	10	3085	NO	NO	NO	NO	YES	NO	NO	NO	NO	NO	13,43	NO	NO	YES
VAGINAL BIRTH	NO	NO	MALE	9	9	2685	NO	NO	NO	NO	YES	NO	NO	NO	NO	NO	13,43	NO	NO	YES
VAGINAL BIRTH	NO	NO	MALE	9	10	3465	NO	NO	NO	NO	YES	NO	NO	NO	NO	NO	13,43	NO	NO	YES
VAGINAL BIRTH	YES	NO	MALE	4	7	3170	YES	NO	NO	NO	YES	NO	NO	NO	NO	YES	13,43	NO	YES	YES
VAGINAL BIRTH	YES	NO	FEMALE	8	9	2650	NO	NO	NO	NO	YES	NO	NO	NO	NO	NO	13,43	NO	NO	YES
VAGINAL BIRTH	NO	NO	FEMALE	9	9	3420	NO	NO	NO	NO	YES	NO	NO	NO	NO	NO	13,43	NO	NO	YES
VAGINAL BIRTH	NO	NO	MALE	9	9	3105	NO	NO	NO	NO	YES	NO	NO	NO	NO	NO	13,43	NO	NO	YES
VAGINAL BIRTH	NO	NO	MALE	7	9	3480	NO	NO	NO	NO	YES	NO	NO	NO	NO	NO	13,43	NO	NO	YES
VAGINAL BIRTH	NO	NO	MALE	7	9	2540	NO	NO	NO	NO	YES	NO	NO	NO	NO	NO	13,43	NO	NO	YES
VAGINAL BIRTH	NO	NO	FEMALE	7	9	3690	NO	NO	NO	NO	YES	NO	NO	NO	NO	NO	13,43	NO	NO	YES
VAGINAL BIRTH	NO	NO	MALE	9	10	3000	NO	NO	NO	NO	YES	NO	NO	NO	NO	NO	13,43	NO	NO	YES
VAGINAL BIRTH	NO	NO	MALE	8	9	2690	YES	NO	NO	NO	YES	NO	NO	NO	NO	NO	13,43	NO	NO	YES
VAGINAL BIRTH	YES	NO	MALE	8	9	3350	NO	NO	NO	NO	YES	NO	NO	NO	NO	NO	13,43	NO	NO	YES
VAGINAL BIRTH	NO	NO	FEMALE	8	10	2995	NO	NO	NO	NO	YES	NO	NO	NO	NO	NO	13,43	NO	NO	YES
VAGINAL BIRTH	YES	NO	FEMALE	9	10	2360	NO	NO	NO	NO	YES	NO	NO	NO	NO	NO	13,57	NO	NO	NO
VAGINAL BIRTH	NO	NO	FEMALE	7	9	3250	NO	NO	NO	NO	YES	NO	NO	NO	NO	NO	13,57	NO	NO	NO
C-SECTIONYES	NO	MALE	9	9	2600	NO	NO	NO	NO	YES	NO	NO	NO	NO	NO	13,57	NO	NO	NO	
C-SECTIONNO	NO	MALE	9	9	2935	NO	NO	NO	NO	YES	NO	NO	NO	NO	NO	13,57	NO	NO	NO	
VAGINAL BIRTH	NO	NO	MALE	8	9	3120	NO	NO	NO	NO	YES	NO	NO	NO	NO	NO	13,57	NO	NO	YES
VAGINAL BIRTH	NO	NO	FEMALE	8	9	2850	NO	NO	NO	NO	YES	NO	NO	NO	NO	NO	13,57	NO	NO	YES
VAGINAL BIRTH	NO	NO	FEMALE	9	9	3120	NO	NO	NO	NO	YES	NO	NO	NO	NO	NO	13,57	NO	NO	YES
C-SECTIONNO	NO	FEMALE	9	10	3725	NO	NO	NO	NO	YES	NO	NO	NO	NO	NO	13,57	NO	NO	YES	
VAGINAL BIRTH	NO	NO	FEMALE	7	8	3195	NO	NO	NO	NO	YES	NO	NO	NO	NO	NO	13,57	NO	NO	YES
C-SECTIONNO	NO	MALE	7	9	3255	NO	NO	NO	NO	YES	NO	NO	NO	NO	NO	13,57	NO	NO	YES	
VAGINAL BIRTH	YES	NO	MALE	8	9	3575	NO	YES	NO	NO	YES	NO	NO	NO	NO	NO	13,57	NO	YES	YES
C-SECTIONNO	NO	MALE	9	10	3415	NO	NO	NO	NO	YES	NO	NO	NO	NO	NO	13,57	NO	NO	YES	
C-SECTIONNO	NO	FEMALE	8	9	2890	YES	NO	NO	NO	YES	NO	NO	NO	NO	NO	13,57	NO	NO	YES	
C-SECTIONNO	NO	FEMALE	9	9	2520	NO	NO	NO	NO	YES	NO	NO	NO	NO	NO	13,57	NO	YES	YES	
VAGINAL BIRTH	NO	NO	MALE	8	9	3160	NO	NO	NO	NO	YES	NO	NO	NO	NO	NO	13,57	NO	NO	YES
VAGINAL BIRTH	NO	NO	MALE	8	9	3080	NO	NO	NO	NO	YES	NO	NO	NO	NO	NO	13,57	NO	NO	YES
VAGINAL BIRTH	NO	NO	MALE	9	10	3480	NO	NO	NO	NO	YES	NO	NO	NO	NO	NO	13,57	NO	NO	YES
C-SECTIONYES	NO	FEMALE	9	10	2930	NO	NO	NO	NO	YES	NO	NO	NO	YES	NO	13,71	NO	NO	NO	
VAGINAL BIRTH	YES	NO	MALE	2	6	540	YES	NO	NO	NO	YES	NO	NO	YES	YES	YES	13,71	NO	NO	NO
VAGINAL BIRTH	NO	NO	FEMALE	8	10	3200	NO	NO	NO	NO	YES	NO	NO	NO	NO	NO	13,71	NO	NO	YES
C-SECTIONNO	NO	FEMALE	8	10	3360	NO	NO	NO	NO	YES	NO	NO	NO	NO	NO	13,71	NO	NO	YES	

VAGINAL BIRTH	NO	NO	FEMALE	8	10	2710	NO	NO	NO	NO	YES	NO	NO	NO	NO	NO	13,71	NO	NO	YES
VAGINAL BIRTH	NO	NO	FEMALE	9	10	3290	NO	NO	NO	NO	YES	NO	NO	NO	NO	NO	13,71	NO	NO	YES
C-SECTIONNO	NO	FEMALE	9	10	2850	NO	NO	NO	NO	YES	NO	NO	NO	NO	NO	13,71	NO	NO	YES	
VAGINAL BIRTH	NO	NO	FEMALE	9	10	3535	NO	NO	NO	NO	YES	NO	NO	NO	NO	NO	13,71	NO	YES	YES
VAGINAL BIRTH	YES	NO	MALE	9	9	3465	NO	NO	NO	NO	YES	NO	NO	NO	NO	NO	13,71	NO	NO	YES
VAGINAL BIRTH	NO	NO	MALE	8	9	3190	NO	NO	NO	NO	YES	NO	NO	NO	NO	NO	13,71	NO	NO	YES
VAGINAL BIRTH	YES	NO	MALE	8	9	3170	NO	NO	YES	NO	YES	NO	NO	NO	NO	NO	13,71	NO	YES	YES
VAGINAL BIRTH	YES	NO	MALE	8	8	2610	YES	NO	NO	NO	YES	NO	NO	NO	NO	NO	13,71	NO	NO	YES
VAGINAL BIRTH	NO	NO	MALE	8	9	3800	NO	NO	NO	NO	YES	NO	NO	NO	NO	NO	13,71	NO	YES	YES
VAGINAL BIRTH	YES	NO	FEMALE	10	10	3405	NO	YES	NO	NO	YES	NO	NO	NO	NO	NO	13,71	NO	NO	YES
VAGINAL BIRTH	YES	NO	MALE	6	7	3630	NO	YES	NO	NO	YES	NO	NO	NO	NO	YES	13,86	NO	YES	NO
VAGINAL BIRTH	NO	NO	FEMALE	9	9	2585	NO	NO	NO	NO	YES	NO	NO	NO	NO	NO	13,86	NO	NO	NO
C-SECTIONNO	NO	FEMALE	8	9	2700	NO	NO	NO	NO	YES	NO	NO	NO	NO	NO	13,86	NO	NO	NO	
C-SECTIONYES	NO	MALE	9	10	3425	NO	NO	NO	NO	YES	NO	NO	NO	YES	NO	13,86	NO	NO	NO	
C-SECTIONNO	NO	MALE	9	10	3655	NO	NO	NO	NO	YES	NO	NO	NO	NO	NO	13,86	NO	NO	NO	
VAGINAL BIRTH	YES	NO	MALE	9	10	3260	NO	NO	NO	NO	YES	NO	NO	NO	NO	NO	13,86	NO	YES	YES
VAGINAL BIRTH	NO	NO	FEMALE	8	10	2750	NO	NO	NO	NO	YES	NO	NO	NO	NO	NO	13,86	NO	NO	YES
VAGINAL BIRTH	NO	NO	MALE	9	9	3320	NO	NO	NO	NO	YES	NO	NO	NO	NO	NO	13,86	NO	NO	YES
VAGINAL BIRTH	NO	NO	MALE	7	9	3885	NO	NO	NO	NO	YES	NO	NO	NO	NO	NO	13,86	NO	NO	YES
VAGINAL BIRTH	NO	NO	MALE	9	10	2900	NO	NO	NO	NO	YES	NO	NO	NO	NO	NO	13,86	NO	NO	YES
VAGINAL BIRTH	NO	NO	MALE	9	9	3280	NO	NO	NO	NO	YES	NO	NO	NO	NO	NO	13,86	NO	YES	YES
VAGINAL BIRTH	YES	NO	MALE	7	8	1040	YES	NO	NO	YES	YES	NO	NO	NO	NO	NO	13,86	NO	NO	YES
VAGINAL BIRTH	YES	NO	FEMALE	9	9	990	YES	NO	NO	YES	YES	NO	NO	NO	NO	NO	13,86	NO	NO	YES
VAGINAL BIRTH	NO	NO	FEMALE	8	9	2850	NO	NO	NO	NO	YES	NO	NO	NO	NO	NO	13,86	NO	NO	YES
VAGINAL BIRTH	YES	NO	FEMALE	9	9	3065	NO	NO	YES	NO	YES	NO	NO	NO	NO	NO	13,86	NO	NO	YES
VAGINAL BIRTH	NO	NO	MALE	9	10	3260	NO	NO	NO	NO	YES	NO	NO	NO	NO	NO	13,86	NO	YES	YES
VAGINAL BIRTH	NO	NO	FEMALE	6	8	2910	NO	NO	NO	NO	YES	NO	NO	NO	NO	YES	13,86	NO	NO	YES
VAGINAL BIRTH	NO	NO	MALE	8	9	3305	NO	NO	NO	NO	YES	NO	NO	NO	NO	NO	13,86	NO	NO	YES
C-SECTIONNO	NO	FEMALE	9	10	3350	NO	NO	NO	NO	YES	NO	NO	NO	NO	NO	13,86	NO	NO	YES	
VAGINAL BIRTH	YES	NO	MALE	9	9	3020	NO	NO	NO	NO	YES	NO	NO	NO	NO	NO	13,86	NO	NO	YES
VAGINAL BIRTH	NO	NO	FEMALE	8	9	3160	NO	NO	NO	NO	YES	NO	NO	NO	NO	NO	13,86	NO	NO	YES
VAGINAL BIRTH	NO	NO	FEMALE	9	9	3130	NO	NO	NO	NO	YES	NO	NO	NO	NO	NO	13,86	NO	NO	YES
VAGINAL BIRTH	NO	NO	FEMALE	8	9	2970	NO	NO	NO	NO	YES	NO	NO	NO	NO	NO	13,86	NO	NO	YES
VAGINAL BIRTH	NO	NO	MALE	9	10	3530	NO	NO	NO	NO	YES	NO	NO	NO	NO	NO	13,86	NO	YES	YES
C-SECTIONYES	NO	MALE	7	9	2710	NO	NO	NO	NO	YES	NO	NO	NO	NO	NO	14,00	YES	NO	NO	
VAGINAL BIRTH	YES	NO	FEMALE	0	0	515		YES	NO	NO	YES	NO	NO	YES	NO	NO	14,43	YES	NO	NO
C-SECTIONYES	NO	FEMALE	9	10	1915	YES	NO	NO	NO	YES	NO	NO	NO	NO	NO	14,57	YES	NO	NO	
C-SECTIONYES	NO	FEMALE	9	9	2915	YES	NO	NO	NO	YES	NO	NO	YES	NO	NO	14,71	YES	NO	NO	
C-SECTIONYES	NO	FEMALE	9	10	3000	YES	YES	NO	NO	YES	NO	NO	NO	NO	NO	14,71	YES	NO	NO	
C-SECTIONYES	NO	MALE	3	9	3160	YES	YES	NO	NO	YES	NO	NO	NO	NO	YES	14,86	YES	NO	NO	
VAGINAL BIRTH	YES	NO	MALE	8	9	2905	NO	NO	NO	NO	YES	NO	NO	NO	NO	NO	14,86	YES	YES	YES
VAGINAL BIRTH	YES	NO	MALE	8	9	2430	NO	NO	NO	NO	YES	NO	NO	NO	NO	NO	15,14	YES	NO	NO
VAGINAL BIRTH	YES	NO	MALE	8	9	3345	NO	NO	NO	NO	YES	NO	NO	NO	NO	NO	15,14	YES	NO	NO
C-SECTIONYES	NO	FEMALE	3	8	2950	YES	NO	NO	NO	YES	NO	NO	YES	NO	YES	15,14	YES	NO	YES	
C-SECTIONYES	NO	FEMALE	7	9	4000	YES	NO	NO	NO	YES	NO	NO	NO	NO	NO	15,14	YES	NO	YES	
C-SECTIONYES	NO	FEMALE	2	4	2525	YES	NO	NO	NO	YES	NO	NO	YES	NO	YES	15,29	YES	NO	NO	
C-SECTIONYES	NO	FEMALE	9	10	2840	YES	NO	NO	NO	YES	NO	NO	NO	NO	NO	15,43	YES	NO	NO	
VAGINAL BIRTH	YES	NO	MALE	2	8	1905	YES	NO	NO	NO	YES	NO	NO	NO	NO	YES	15,43	YES	YES	YES
VAGINAL BIRTH	YES	NO	MALE	0	0	2595		NO	NO	NO	YES	NO	NO	YES	NO	NO	16,00	YES	NO	NO
VAGINAL BIRTH	YES	NO	MALE	8	8	4140	NO	NO	NO	NO	YES	NO	NO	NO	NO	NO	16,57	YES	YES	YES
C-SECTIONYES	NO	MALE	8	8	2660	YES	NO	NO	NO	YES	NO	NO	NO	NO	NO	17,57	YES	NO	NO	
VAGINAL BIRTH	YES	NO	MALE	0	0	475		NO	NO	NO	YES	NO	NO	YES	NO	NO	17,86	YES	NO	NO
VAGINAL BIRTH	YES	NO	MALE	9	10	3735	NO	NO	NO	NO	YES	NO	NO	NO	NO	NO	18,00	YES	NO	NO

VAGINAL BIRTH	YES	NO	MALE	9	10	2750	NO	NO	NO	NO	YES	NO	NO	NO	NO	NO	18,14	YES	NO	NO
VAGINAL BIRTH	YES	NO	MALE	0	0	1340		NO	NO	NO	YES	NO	NO	NO	NO	NO	18,14	YES	YES	YES
VAGINAL BIRTH	YES	NO	MALE	9	10	2700	NO	NO	NO	NO	YES	NO	NO	NO	NO	NO	18,14	YES	NO	YES
C-SECTIONYES	NO	FEMALE	8	9	3000	YES	NO	YES	NO	YES	NO	NO	NO	NO	NO	18,57	YES	NO	YES	
VAGINAL BIRTH	YES	NO	FEMALE	1	1	530	NO	NO	NO	NO	YES	NO	NO	YES	NO	NO	19,43	YES	NO	NO
VAGINAL BIRTH	YES	NO	FEMALE	8	8	2195	YES	NO	NO	NO	YES			NO	NO	NO	19,43	YES	NO	YES
VAGINAL BIRTH	YES	NO	FEMALE	1	1	245	NO	NO	NO	NO	YES	NO	NO	YES	NO	NO	19,71	YES	NO	NO
VAGINAL BIRTH	YES	NO	FEMALE	8	8	2525	NO	NO	NO	NO	YES	NO	NO	NO	NO	NO		YES	NO	YES
C-SECTIONYES	NO	MALE	7	8	4055	YES	NO	NO	NO	YES	NO	NO	NO	NO	NO		YES	NO	NO	
VAGINAL BIRTH	YES	NO	MALE			630		NO	NO	NO	YES	NO	NO	YES	NO	NO		YES	NO	NO
VAGINAL BIRTH	YES	NO	FEMALE	7	9	1670	YES	NO	NO	NO	YES	NO	NO	YES	NO	NO		YES	NO	YES
C-SECTIONYES	NO	MALE	8	9	2615	YES	NO	NO	NO	YES	NO	NO	NO	NO	NO		YES	NO	NO	
VAGINAL BIRTH	YES	NO	FEMALE	3	6	3220	YES	NO	NO	NO	YES	NO	NO	NO	YES	YES		YES	NO	NO
VAGINAL BIRTH	YES	NO	MALE	2	6	3275	YES	YES	NO	NO	YES	NO	NO	NO	NO	YES		YES	YES	NO
VAGINAL BIRTH	YES	YES	MALE	0	0	3130		NO	NO	NO	YES	NO	NO	YES	NO	NO		YES	NO	NO
VAGINAL BIRTH	YES	NO	MALE	9	10	3200	NO	NO	NO	NO	YES	NO	NO	NO	NO	NO		YES	NO	NO
VAGINAL BIRTH	YES	NO	FEMALE	8	10	3345	NO	NO	NO	NO	YES	NO	NO	NO	NO	NO		YES	NO	NO
VAGINAL BIRTH	YES	NO	MALE	0	0			NO	NO	NO	YES	NO		YES	NO	NO		YES	NO	NO
VAGINAL BIRTH	YES	NO	MALE	0	0	765		NO	NO	NO	YES	NO	NO	YES	NO	NO		YES	YES	NO
VAGINAL BIRTH	YES	NO	MALE	9	9	3540	NO	NO	NO	NO	YES	NO	NO	NO	NO	NO		YES	NO	NO
C-SECTIONYES	NO	MALE	8	9	3070	NO	YES	NO	NO	YES	NO	NO	NO	NO	NO		YES	NO	NO	
VAGINAL BIRTH	YES	NO	MALE	7	9	4570	NO	NO	NO	NO	YES	NO	NO	NO	NO	NO		YES	YES	NO
VAGINAL BIRTH	YES	NO	FEMALE	8	9	3180	NO	NO	NO	NO	YES	NO	NO	NO	NO	NO		YES	YES	NO
VAGINAL BIRTH	YES	NO	FEMALE	0	0	580		NO	NO	NO	YES	NO	NO	YES	YES	NO		YES	NO	NO
VAGINAL BIRTH	YES	NO	FEMALE	9	10	3030	NO	NO	NO	NO	YES	NO	NO	NO	NO	NO		YES	YES	NO
C-SECTIONYES	NO	MALE	3	8	3100	NO	NO	NO	NO	YES	NO	NO	NO	NO	YES		YES	YES	NO	
C-SECTIONYES	NO	FEMALE	7	8	3560	NO	NO	NO	NO	YES	NO	YES	NO	NO	NO		YES	NO	NO	
C-SECTIONYES	NO	FEMALE	3	9	2165	YES	NO	NO	NO	YES	NO	NO	NO	NO	YES		YES	NO	NO	
C-SECTIONYES	NO	MALE	8	8	3350	YES	NO	NO	NO	YES	NO	NO	NO	NO	NO		YES	NO	NO	
C-SECTIONYES	NO	MALE	9	9	3175	YES	NO	NO	NO	YES	NO	NO	NO	NO	NO		YES	NO	NO	
C-SECTIONYES	NO	FEMALE	8	9	3400	YES	NO	NO	NO	YES	NO	NO	NO	NO	NO		YES	NO	NO	
C-SECTIONYES	NO	MALE	7	9	2995	YES	NO	NO	NO	YES	NO	NO	NO	NO	NO		YES	NO	NO	
C-SECTIONYES	NO	MALE	5	7	2375	YES	NO	NO	NO	YES	NO	NO	YES	NO	YES		YES	NO	NO	
C-SECTIONYES	NO	MALE			815		NO	NO	NO	YES	NO	NO	YES	NO	NO		YES	NO	NO	
C-SECTIONYES	NO	FEMALE	1	8	2455	YES	NO	NO	NO	YES	NO	NO	NO	NO	YES		YES	NO	YES	
VAGINAL BIRTH	YES	NO	FEMALE	8	9	2890	NO	NO	NO	NO	YES	NO	NO	NO	NO	NO		YES	YES	YES
VAGINAL BIRTH	YES	NO	MALE	0	0	1185		NO	NO	NO	YES	NO	NO	YES	NO	NO		YES	NO	YES
VAGINAL BIRTH	YES	NO	MALE	1	1	1820	NO	NO	NO	NO	YES	NO	NO	YES	NO	NO		YES	NO	YES
C-SECTIONYES	NO	MALE	8	9	1720	YES	NO	NO	NO	NO	NO	NO	NO	NO	NO		YES	NO	YES	
VAGINAL BIRTH	YES	NO	FEMALE	9	10	2845	NO	NO	NO	NO	YES	NO	NO	NO	NO	NO		YES	NO	YES
VAGINAL BIRTH	YES	NO	FEMALE	8	8	3115	NO	NO	NO	NO	NO	NO	NO	NO	NO	NO		YES	NO	YES
VAGINAL BIRTH	YES	NO	MALE	7	8	3110	YES	NO	NO	NO	YES	NO	NO	NO	YES	NO		YES	NO	YES
VAGINAL BIRTH	YES	NO	FEMALE	9	10	2875	NO	NO	NO	NO	YES	NO	NO	NO	NO	NO		YES	NO	YES
C-SECTIONYES	NO	FEMALE	8	9	3625	NO	NO	NO	NO	YES	NO	NO	NO	NO	NO		YES	YES	YES	
VAGINAL BIRTH	YES	NO	FEMALE	3	7	1000	YES	NO	NO	NO	YES	NO	NO	YES	NO	YES		YES	NO	YES
VAGINAL BIRTH	YES	NO	MALE	8	8	3305	YES	NO	NO	NO	YES	NO	NO	NO	NO	NO		YES	NO	YES
VAGINAL BIRTH	YES	NO		0	0	532		NO	NO	NO	YES	NO	NO	NO	NO	NO	20,00	YES	NO	NO
C-SECTIONYES	NO	FEMALE	9	10	2800	YES	NO	NO	NO	YES	NO	NO	NO	NO	NO	20,00	YES	NO	NO	
C-SECTIONYES	NO	MALE	4	8	1800	NO	NO	NO	NO	YES	NO	NO	NO	NO	YES	20,57	YES	NO	YES	
VAGINAL BIRTH	YES	NO	FEMALE	9	10	3285	NO	NO	NO	NO	YES	NO	NO	NO	NO	NO	21,00	YES	NO	YES
C-SECTIONYES	NO	MALE	9	10	2110	YES	NO	YES	YES	YES	NO	NO	NO	NO	NO	21,14	YES	NO	NO	
C-SECTIONYES	NO	MALE	9	10	2880	NO	NO	YES	YES	YES	NO	NO	NO	NO	NO	21,14	YES	NO	NO	
VAGINAL BIRTH	YES	NO	MALE	1	0	2535	NO	NO	NO	NO	YES	NO	NO	NO	NO	YES	21,29	YES	NO	YES

C-SECTIONYES	NO	FEMALE	9	10	2815	YES	NO	NO	NO	YES	NO	NO	NO	NO	NO	21,43	YES	NO	NO	
VAGINAL BIRTH	YES	NO	FEMALE	0	0	1415		NO	NO	NO	YES	NO	NO	YES	NO	NO	21,86	YES	NO	NO
C-SECTIONYES	NO	MALE	9	9	3270	NO	NO	NO	NO	YES	NO	NO	NO	NO	NO	22,00	YES	NO	NO	
VAGINAL BIRTH	YES	NO	MALE	8	9	3085	YES	NO	NO	NO	YES	NO	NO	YES	NO	NO	22,14	YES	NO	YES
VAGINAL BIRTH	YES	NO	MALE	0	0	405		NO	NO	NO	YES	NO	NO	YES	NO	NO	22,29	YES	NO	NO
VAGINAL BIRTH	YES	NO	MALE	8	9	3085	NO	NO	NO	NO	YES	NO	NO	NO	NO	NO	22,57	YES	NO	YES
VAGINAL BIRTH	YES	NO	MALE	0	0	2265		NO	NO	NO	YES	NO	NO	YES	NO	NO	22,86	YES	NO	NO
VAGINAL BIRTH	YES	NO	MALE	0	0	1355		NO	NO	NO	YES	NO	NO	YES	NO	NO	23,00	YES	NO	NO
C-SECTIONYES	NO	MALE	8	9	2785	NO	NO	NO	NO	YES	NO	NO	NO	NO	NO	23,00	YES	NO	NO	
C-SECTIONYES	NO	MALE	4	9	1540	YES	NO	NO	NO	YES	NO	NO	NO	NO	YES	23,71	YES	NO	YES	
VAGINAL BIRTH	YES	NO	FEMALE	8	9	3690	YES	NO	NO	NO	YES	NO	NO	NO	NO	NO	24,29	YES	NO	NO
C-SECTIONYES	NO	MALE	2	8	2740	YES	NO	NO	NO	YES	NO	NO	YES	NO	YES	24,29	YES	NO	YES	
C-SECTIONYES	NO	FEMALE	9	9	2700	YES	NO	NO	NO	YES	NO	NO	NO	NO	NO	24,43	YES	NO	NO	
VAGINAL BIRTH	YES	NO	FEMALE	1	1	1765	NO	NO	NO	NO	YES	NO	NO	YES	NO	NO	24,43	YES	NO	YES
C-SECTIONYES	NO	FEMALE	9	10	3780	NO	NO	NO	NO	YES	NO	NO	NO	NO	NO	27,14	YES	NO	NO	
VAGINAL BIRTH	YES	NO	FEMALE	7	8	1705	YES	NO	NO	NO	YES	NO	NO	YES	NO	NO	27,43	YES	NO	YES
C-SECTIONYES	NO	FEMALE	8	9	4040	YES	NO	NO	NO	YES	NO	NO	NO	NO	NO	31,29	YES	NO	YES	
C-SECTIONYES	NO	MALE	8	9	3485	YES	NO	NO	NO	YES	NO	NO	NO	NO	NO	33,00	YES	NO	YES	
VAGINAL BIRTH	YES	NO	FEMALE	8	9	2290	YES	YES	NO	NO	YES	NO	NO	NO	NO	NO	33,43	YES	NO	YES
VAGINAL BIRTH	YES	NO	FEMALE	9	9	3955	NO	NO	YES	NO	YES	NO	NO	NO	NO	NO	39,00	YES	NO	NO
C-SECTIONYES	NO	MALE	9	9	3580	YES	YES	YES	NO	YES	NO	NO	NO	NO	NO	5,00	YES	NO	NO	
VAGINAL BIRTH	YES	NO	MALE	9	10	3940	NO	NO	NO	NO	YES	NO	NO	NO	NO	NO	6,00	YES	NO	NO
C-SECTIONYES	NO	FEMALE	7	9	3070	YES	NO	YES	NO	YES	NO	NO	NO	NO	NO	6,00	YES	NO	NO	
C-SECTIONYES	NO	FEMALE	9	9	3160	NO	NO	NO	NO	YES	NO	NO	NO	NO	NO	6,00	YES	NO	YES	
VAGINAL BIRTH	YES	NO	FEMALE	0	2	1705	YES	NO	YES	NO	YES	NO	NO	YES	NO	YES	6,14	YES	NO	NO
VAGINAL BIRTH	YES	NO	MALE	0	0	1085		NO	YES	NO	YES	NO	NO	YES	NO	NO	6,29	YES	NO	NO
VAGINAL BIRTH	YES	NO	MALE	8	9	2550	YES	YES	YES	NO	YES	NO	NO	NO	NO	NO	6,29	YES	NO	YES
VAGINAL BIRTH	YES	NO	MALE	0	0	430		NO	NO	NO	YES	NO	NO	YES	NO	NO	6,43	YES	NO	NO
VAGINAL BIRTH	YES	NO	MALE	7	9	2105	NO	NO	NO	NO	YES	NO	NO	NO	NO	NO	6,43	YES	NO	YES
C-SECTIONYES	NO	MALE	3	7	2755	YES	YES	NO	NO	YES	NO	NO	NO	NO	YES	6,57	YES	NO	NO	
VAGINAL BIRTH	YES	NO	MALE	8	9	2850	YES	YES	YES	NO	YES	NO	NO	NO	NO	NO	6,57	YES	YES	NO
C-SECTIONYES	NO	FEMALE	2	7	2060	YES	NO	NO	NO	YES	NO	NO	YES	NO	YES	6,86	YES	NO	NO	
C-SECTIONYES	NO	MALE	1	1	1092	NO	YES	NO	NO	YES	NO	NO	YES	NO	NO	7,00	YES	NO	NO	
C-SECTIONYES	NO	MALE	8	8	1975	YES	NO	NO	NO	YES	NO	NO	NO	NO	NO	7,00	YES	NO	NO	
C-SECTIONYES	NO	MALE	9	10	2925	YES	NO	NO	NO	YES	NO	NO	NO	NO	NO	7,00	YES	NO	NO	
VAGINAL BIRTH	YES	NO	MALE	10	10	2885	NO	NO	YES	NO	YES	NO	NO	NO	NO	NO	7,14	YES	YES	NO
VAGINAL BIRTH	YES	NO	MALE	0	0	2670		NO	NO	NO	YES	NO	NO	YES	NO	NO	7,14	YES	NO	NO
C-SECTIONYES	NO	FEMALE	8	8	2500	NO	NO	NO	NO	YES	NO	NO	NO	NO	NO	7,14	YES	NO	YES	
C-SECTIONYES	NO	MALE	4	9	2910	YES	YES	NO	NO	YES	NO	NO	NO	NO	YES	7,29	YES	NO	NO	
VAGINAL BIRTH	YES	NO	FEMALE	8	9	2305	NO	YES	NO	NO	YES	NO	NO	NO	NO	NO	7,43	YES	YES	NO
C-SECTIONYES	NO	MALE	9	9	3180	YES	NO	NO	NO	YES	NO	NO	NO	NO	NO	7,43	YES	NO	NO	
VAGINAL BIRTH	YES	NO	MALE	8	10	2950	NO	NO	NO	NO	YES	NO	NO	NO	NO	NO	7,43	YES	YES	YES
VAGINAL BIRTH	YES	NO	FEMALE	6	8	2155	YES	NO	NO	NO	YES	NO	NO	NO	NO	YES	7,57	YES	NO	NO
VAGINAL BIRTH	YES	YES	MALE	3	8	1760	YES	NO	NO	NO	YES	NO	NO	YES	NO	YES	7,57	YES	NO	YES
C-SECTIONYES	NO	MALE	9	9	1680	YES	NO	YES	NO	YES	NO	NO	NO	NO	NO	7,71	YES	NO	NO	
C-SECTIONYES	NO	MALE	3	8	3125	YES	NO	NO	NO	YES	NO	NO	NO	NO	YES	7,86	YES	NO	NO	
C-SECTIONYES	NO	FEMALE	5	9	2985	YES	NO	NO	NO	YES	NO	NO	NO	NO	YES	7,86	YES	NO	NO	
C-SECTIONYES	NO	MALE	7	9	3090	YES	NO	YES	NO	YES	NO	NO	NO	NO	NO	7,86	YES	NO	NO	
VAGINAL BIRTH	YES	NO	FEMALE	0	0	565		NO	NO	NO	YES	NO	NO	YES	NO	NO	8,00	YES	NO	NO
VAGINAL BIRTH	YES	NO	MALE	8	10	3085	YES	YES	YES	NO	YES	NO	NO	NO	YES	NO	8,00	YES	NO	NO
VAGINAL BIRTH	YES	NO	FEMALE	0	0	315		NO	NO	NO	YES	NO	NO	YES	NO	NO	8,14	YES	NO	NO
C-SECTIONYES	NO	MALE	9	9	2710	YES	NO	NO	NO	YES	NO	NO	NO	NO	NO	8,14	YES	NO	YES	
VAGINAL BIRTH	YES	NO	MALE	1	1	2815	NO	NO	NO	NO	YES	NO	NO	YES	NO	NO	8,14	YES	NO	YES

C-SECTIONYES	NO	FEMALE	4	8	3980	YES	NO	NO	NO	YES	NO	YES	NO	NO	YES	8,29	YES	NO	NO	
VAGINAL BIRTH	YES	NO	FEMALE	8	9	2830	NO	NO	NO	NO	YES	NO	NO	NO	NO	NO	8,29	YES	YES	YES
C-SECTIONYES	NO	FEMALE	7	9	3250	NO	YES	NO	NO	YES	NO	NO	NO	NO	NO	8,57	YES	NO	NO	
C-SECTIONYES	NO	MALE	9	9	3485	YES	NO	NO	NO	YES	NO	NO	NO	NO	NO	8,71	YES	NO	NO	
C-SECTIONYES	NO	MALE	8	9	2690	YES	NO	NO	NO	YES	NO	NO	NO	NO	NO	8,71	YES	NO	NO	
C-SECTIONYES	NO	FEMALE	4	7	4035	YES	NO	NO	NO	YES	NO	NO	NO	NO	YES	8,71	YES	NO	NO	
VAGINAL BIRTH	YES	NO	MALE	9	9	3805	NO	NO	NO	NO	YES	NO	NO	NO	NO	NO	8,71	YES	NO	YES
C-SECTIONYES	NO	FEMALE	4	8	3240	YES	NO	NO	NO	YES	NO	NO	NO	NO	YES	8,71	YES	NO	YES	
C-SECTIONYES	NO	FEMALE	7	8	3205	YES	NO	NO	NO	YES	NO	NO	NO	NO	NO	8,86	YES	NO	NO	
C-SECTIONYES	NO	UNDETERMINATED		3	5	2800	YES	NO	NO	NO	YES	NO	NO	YES	NO	YES	8,86	YES	NO	NO
VAGINAL BIRTH	YES	NO	MALE	4	2	2260	YES	YES	NO	NO	YES	NO	NO	YES	NO	YES	9,00	YES	YES	NO
VAGINAL BIRTH	YES	NO	FEMALE	1	1	1000	NO	NO	NO	NO	YES	NO	NO	YES	NO	NO	9,14	YES	NO	NO
VAGINAL BIRTH	YES	NO	MALE	6	8	1490	YES	NO	NO	NO	YES	NO	NO	NO	NO	YES	9,14	YES	NO	YES
VAGINAL BIRTH	YES	NO	MALE	8	9	2065	YES	NO	NO	NO	YES	NO	NO	NO	NO	NO	9,29	YES	NO	NO
C-SECTIONYES	NO	FEMALE	6	8	3340	YES	NO	NO	NO	YES	NO	NO	NO	NO	YES	9,29	YES	NO	NO	
VAGINAL BIRTH	YES	NO	FEMALE	2	9	3110	YES	NO	NO	NO	YES	NO	NO	NO	NO	YES	9,29	YES	NO	YES
C-SECTIONYES	NO	FEMALE	8	9	1970	YES	NO	NO	NO	YES	NO	NO	YES	NO	NO	9,43	YES	NO	NO	
C-SECTIONYES	NO	FEMALE	9	10	2675	YES	NO	YES	NO	YES	NO	NO	NO	NO	NO	9,43	YES	NO	NO	
VAGINAL BIRTH	YES	NO	FEMALE	7	7	1190	NO	NO	YES	NO	YES	NO	NO	YES	NO	NO	9,43	YES	NO	NO
C-SECTIONYES	NO	FEMALE	8	9	3505	NO	NO	NO	NO	YES	NO	NO	NO	NO	NO	9,43	YES	NO	NO	
C-SECTIONYES	NO	FEMALE	8	9	3340	YES	NO	NO	NO	YES	NO	NO	NO	NO	NO	9,43	YES	NO	NO	
VAGINAL BIRTH	YES	NO	MALE	1	1	1915	NO	NO	NO	NO	YES	NO	NO	YES	NO	YES	9,43	YES	NO	YES
VAGINAL BIRTH	YES	NO	FEMALE	0	0	1105		NO	NO	NO	YES	NO	NO	NO	NO	NO	9,57	YES	NO	NO
VAGINAL BIRTH	YES	NO	FEMALE	4	9	3305	NO	NO	NO	NO	YES	NO	NO	NO	NO	YES	9,57	YES	YES	NO
VAGINAL BIRTH	YES	NO	FEMALE	7	10	2230	YES	NO	NO	NO	YES	NO	NO	NO	NO	NO	9,57	YES	NO	NO
C-SECTIONYES	NO	MALE	8	9	2970	NO	NO	NO	NO	YES	NO	NO	NO	NO	NO	9,57	YES	NO	NO	
C-SECTIONYES	NO	MALE	9	10	3085	YES	NO	NO	NO	YES	NO	NO	NO	NO	NO	9,57	YES	NO	NO	
VAGINAL BIRTH	YES	YES	FEMALE	6	8	2795	YES	NO	NO	NO	YES	NO	NO	NO	NO	YES	9,57	YES	YES	YES
VAGINAL BIRTH	YES	NO	FEMALE	8	9	3345	YES	NO	NO	NO	YES	NO	NO	NO	NO	NO	9,57	YES	YES	YES
VAGINAL BIRTH	YES	NO	FEMALE	0	0	1270		NO	NO	NO	YES	NO	NO	YES	NO	NO	9,57	YES	NO	YES
VAGINAL BIRTH	YES	NO	FEMALE	9	10	1815	YES	NO	NO	NO	YES	NO	NO	NO	NO	NO	9,71	YES	NO	YES
VAGINAL BIRTH	YES	NO	FEMALE	0	0	1110		YES	NO	NO	YES	NO	NO	YES	NO	NO	10,00	YES	NO	NO
VAGINAL BIRTH	YES	NO	MALE	0	0	880		NO	NO	NO	YES	NO	NO	YES	NO	NO	10,00	YES	NO	NO
VAGINAL BIRTH	YES	NO	FEMALE	7	8	2460	NO	NO	NO	NO	YES	NO	NO	NO	NO	NO	10,00	YES	NO	NO
VAGINAL BIRTH	YES	NO	FEMALE	6	9	2510	YES	NO	NO	NO	YES	NO	NO	NO	NO	YES	10,00	YES	YES	YES
VAGINAL BIRTH	YES	NO	MALE	2	9	3375	NO	YES	NO	NO	YES	NO	NO	NO	NO	YES	10,00	YES	NO	YES
C-SECTIONYES	NO	MALE	2	9	2095	YES	NO	NO	NO	YES	NO	NO	NO	NO	YES	10,00	YES	NO	YES	
C-SECTIONYES	NO	MALE	8	10	3340	YES	YES	NO	NO	YES	NO	NO	NO	NO	NO	10,14	YES	NO	NO	
VAGINAL BIRTH	YES	NO	FEMALE	3	1	2310	NO	NO	NO	NO	YES	NO	NO	YES	NO	NO	10,14	YES	YES	YES
C-SECTIONYES	NO	MALE	7	8	1810	YES	NO	NO	NO	YES	NO	NO	YES	NO	NO	10,29	YES	NO	NO	
VAGINAL BIRTH	YES	NO	UNDETERMINATED		0	0	280		NO	NO	NO	YES	NO	NO	NO	NO	NO	10,29	YES	NO
YES																				
VAGINAL BIRTH	YES	NO	FEMALE	9	10	2660	NO	NO	NO	NO	YES	NO	NO	NO	NO	NO	10,29	YES	NO	YES
VAGINAL BIRTH	YES	NO	MALE	4	8	2725	YES	NO	NO	NO	YES	NO	NO	YES	NO	YES	10,43	YES	NO	NO
C-SECTIONYES	NO	MALE	8	9	3840	NO	NO	NO	NO	YES	NO	NO	NO	NO	NO	10,43	YES	NO	NO	
VAGINAL BIRTH	YES	NO	FEMALE	9	10	3380	NO	NO	NO	NO	YES	NO	NO	NO	NO	NO	10,43	YES	NO	YES
VAGINAL BIRTH	YES	NO	MALE	9	10	3385	NO	NO	NO	NO	YES	NO	NO	NO	NO	NO	10,57	YES	NO	NO
VAGINAL BIRTH	YES	NO	MALE	7	9	2860	NO	NO	NO	NO	YES	NO	NO	NO	NO	NO	10,57	YES	NO	NO
VAGINAL BIRTH	YES	NO	MALE			2012		NO	NO	NO	YES	NO	NO	YES	NO	NO	10,57	YES	YES	YES
VAGINAL BIRTH	YES	NO	MALE	8	9	3740	NO	YES	NO	NO	YES	NO	NO	NO	NO	NO	11,00	YES	NO	YES
C-SECTIONYES	NO	FEMALE	8	9	2650	YES	NO	NO	NO	YES	NO	NO	NO	NO	NO	11,14	YES	NO	NO	
C-SECTIONYES	NO	FEMALE	9	9	1710	NO	NO	NO	NO	YES	NO	NO	NO	NO	NO	11,14	YES	NO	NO	
VAGINAL BIRTH	YES	NO	FEMALE	3	5	3100	YES	NO	NO	NO	YES	NO	NO	YES	NO	YES	11,14	YES	NO	YES

C-SECTIONYES	NO	MALE	9	10	3110	NO	NO	NO	NO	YES	NO	NO	NO	YES	NO	11,14	YES	NO	YES	
C-SECTIONYES	NO	MALE	9	10	4060	NO	NO	NO	NO	YES	NO	NO	NO	NO	NO	11,14	YES	NO	YES	
VAGINAL BIRTH	YES	NO	FEMALE			1635		NO	NO	NO	YES	NO	NO	YES	NO	NO	11,29	YES	YES	NO
VAGINAL BIRTH	YES	NO	FEMALE	8	9	1840	YES	NO	NO	NO	YES	NO	NO	NO	NO	NO	11,29	YES	NO	NO
VAGINAL BIRTH	YES	NO	FEMALE	1	4	2580	NO	NO	NO	NO	YES	NO	NO	YES	NO	YES	11,29	YES	NO	NO
C-SECTIONYES	NO	FEMALE	5	9	2850	YES	NO	NO	NO	YES	NO	NO	NO	NO	YES	11,29	YES	NO	NO	
VAGINAL BIRTH	YES	NO	MALE	9	9	3570	NO	NO	NO	NO	YES	NO	NO	NO	NO	NO	11,43	YES	YES	YES
C-SECTIONYES	NO	FEMALE	8	9	2305	YES	NO	YES	NO	YES	NO	NO	YES	NO	NO	11,57	YES	NO	NO	
VAGINAL BIRTH	YES	NO	FEMALE	8	9	3150	YES	NO	NO	NO	YES	NO	NO	NO	NO	NO	11,86	YES	NO	YES
VAGINAL BIRTH	YES	NO	MALE	9	10	3700	NO	NO	NO	NO	YES	NO	NO	NO	NO	NO	11,86	YES	NO	YES
C-SECTIONYES	NO	FEMALE	7	9	2420	YES	NO	NO	NO	YES	NO	NO	YES	NO	NO	12,00	YES	NO	NO	
C-SECTIONYES	NO	FEMALE	9	9	2650	YES	NO	NO	NO	YES	NO	NO	NO	NO	NO	12,00	YES	NO	NO	
C-SECTIONYES	NO	MALE	9	9	2455	YES	NO	NO	NO	YES	NO	NO	NO	NO	NO	12,00	YES	NO	NO	
VAGINAL BIRTH	YES	YES	MALE	2	6	2960	YES	NO	NO	NO	YES	NO	NO	YES	NO	YES	12,00	YES	YES	YES
VAGINAL BIRTH	YES	NO	FEMALE	2	5	1115	YES	NO	NO	NO	YES	NO	NO	YES	NO	YES	12,00	YES	NO	YES
C-SECTIONYES	NO	FEMALE	0	0	4190		NO	YES	NO	YES	NO	NO	YES	NO	YES	12,00	YES	NO	YES	
VAGINAL BIRTH	YES	NO	FEMALE	9	10	2520	YES	NO	NO	NO	YES	NO	NO	NO	NO	NO	12,00	YES	NO	YES
VAGINAL BIRTH	YES	NO	MALE	8	8	3420	NO	NO	NO	NO	YES	NO	NO	NO	NO	NO	12,14	YES	NO	NO
C-SECTIONYES	NO	MALE	8	9	3100	YES	NO	NO	NO	YES	NO	NO	NO	NO	NO	12,14	YES	NO	NO	
C-SECTIONYES	NO	MALE	3	8	3515	YES	NO	NO	NO	YES	NO	NO	NO	NO	YES	12,14	YES	NO	NO	
VAGINAL BIRTH	YES	NO	FEMALE	0	2	1405	NO	NO	NO	NO	YES	NO	NO	YES	NO	NO	12,14	YES	NO	YES
C-SECTIONYES	NO	MALE	7	8	2110	YES	NO	NO	NO	YES	NO	NO	NO	NO	NO	12,29	YES	NO	NO	
C-SECTIONYES	NO	MALE	2	1	1815	NO	NO	NO	NO	YES	NO	NO	YES	NO	YES	12,29	YES	NO	NO	
C-SECTIONYES	NO	MALE	9	9	3940	NO	NO	NO	NO	YES	NO	NO	NO	NO	NO	12,29	YES	NO	NO	
VAGINAL BIRTH	YES	NO	MALE	7	8	3635	YES	NO	NO	NO	YES	NO	NO	NO	YES	NO	12,29	YES	NO	YES
VAGINAL BIRTH	YES	NO	FEMALE	9	9	1810	YES	NO	NO	NO	YES	NO	NO	NO	NO	NO	12,43	YES	NO	YES
VAGINAL BIRTH	YES	NO	MALE	8	9	3520	NO	NO	NO	NO	YES	NO	NO	NO	NO	NO	12,43	YES	NO	YES
C-SECTIONYES	NO	MALE	6	9	1900	YES	NO	NO	NO	YES	NO	NO	YES	NO	YES	12,43	YES	NO	YES	
VAGINAL BIRTH	YES	NO	FEMALE	1	1	1128	NO	NO	NO	NO	YES	NO	NO	NO	NO	NO	12,43	YES	NO	YES
C-SECTIONYES	NO	FEMALE	4	8	1734	YES	NO	NO	NO	YES	NO	NO	NO	NO	YES	12,57	YES	NO	NO	
VAGINAL BIRTH	YES	NO	MALE	8	8	3100	NO	NO	NO	NO	YES	NO	NO	NO	NO	NO	12,71	YES	NO	NO
VAGINAL BIRTH	YES	NO	MALE	8	9	1925	YES	NO	NO	NO	YES	NO	NO	NO	NO	NO	12,86	YES	NO	NO
C-SECTIONYES	NO	MALE	8	8	2630	YES	NO	NO	NO	YES	NO	NO	NO	NO	NO	12,86	YES	NO	NO	
C-SECTIONYES	NO	FEMALE	0	0	3000	NO	NO	NO	NO	YES	NO	NO	YES	NO	NO	12,86	YES	NO	NO	
VAGINAL BIRTH	YES	NO	FEMALE	8	9	3555	NO	NO	NO	NO	YES	NO	NO	NO	NO	NO	13,00	YES	NO	NO
C-SECTIONYES	NO	MALE	7	8	3385	YES	YES	YES	NO	YES	NO	NO	NO	NO	NO	13,00	YES	NO	NO	
C-SECTIONYES	NO	MALE	8	9	3290	NO	YES	YES	NO	YES	NO	NO	NO	NO	NO	13,00	YES	NO	NO	
VAGINAL BIRTH	YES	NO	FEMALE	8	9	3595	NO	NO	NO	NO	YES	NO	NO	NO	NO	NO	13,14	YES	NO	NO
C-SECTIONYES	NO	MALE	9	9	2655	NO	NO	NO	NO	YES	NO	NO	NO	NO	NO	13,14	YES	NO	NO	
VAGINAL BIRTH	YES	NO	FEMALE	8	9	3230	NO	NO	NO	NO	YES	NO	NO	NO	YES	NO	13,29	YES	YES	NO
VAGINAL BIRTH	YES	NO	MALE	9	10	2025	NO	NO	NO	NO	YES	NO	NO	NO	NO	NO	13,43	YES	YES	NO
VAGINAL BIRTH	YES	NO	MALE	9	10	2580	YES	NO	NO	NO	YES	NO	NO	NO	NO	NO	13,43	YES	NO	NO
C-SECTIONYES	NO	FEMALE	7	10	2100	YES	NO	NO	NO	YES	NO	NO	NO	NO	NO	13,43	YES	NO	NO	
C-SECTIONYES	NO	FEMALE	7	9	2510	YES	NO	NO	NO	YES	NO	NO	NO	NO	NO	13,43	YES	NO	NO	
VAGINAL BIRTH	YES	NO	MALE	9	10	3190	NO	NO	NO	NO	YES	NO	NO	NO	NO	NO	13,43	YES	NO	YES
C-SECTIONYES	NO	MALE	8	8	2185	YES	NO	NO	NO	YES	NO	NO	NO	NO	NO	13,57	YES	NO	NO	
C-SECTIONYES	NO	FEMALE	8	9	3585	NO	NO	NO	NO	YES			NO	NO	NO	13,57	YES	NO	NO	
VAGINAL BIRTH	YES	NO	FEMALE	0	0	1660		NO	NO	NO	YES	NO	NO	YES	NO	NO	13,71	YES	YES	NO
VAGINAL BIRTH	YES	NO	UNDETERMINATED		0	0	930		NO	NO	NO	YES	NO	NO	YES	NO	NO	13,71	YES	NO
NO																				
C-SECTIONYES	NO	FEMALE	8	8	2875	NO	NO	NO	NO	YES	NO	NO	NO	NO	NO	13,71	YES	NO	NO	
C-SECTIONYES	NO	MALE	8	9	3455	YES	NO	NO	NO	YES	NO	NO	YES	NO	NO	13,86	YES	NO	NO	
